# Photophysical and Photochemical Properties of a Curcumins Family: A Combined Computational and Experimental Investigation

**DOI:** 10.1021/acsomega.5c10926

**Published:** 2026-02-04

**Authors:** Ali Ghiami-Shomami, Silvia Ruggieri, Silvia Mizzoni, Fabio Piccinelli, Francesca Terenziani, Riccardo Pettinari, Noemi Pagliaricci, Sara Pagliaricci, Andrea Melchior

**Affiliations:** † Polytechnic Department of Engineering, Chemical Technologies Laboratories, University of Udine, Via Cotonificio 108, 33100 Udine, Italy; ‡ Luminescent Materials Laboratory DB, University of Verona, and INSTM, UdR Verona, Strada Le Grazie 15, 37134 Verona, Italy; § Department of Chemistry, Life Sciences and Environmental Sustainability, University of Parma, Parco Area delle Scienze 17/a, 43124 Parma, Italy; ∥ School of Pharmacy, 18959 University of Camerino, via Madonna delle Carceri, 62032 Camerino, MC, Italy

## Abstract

In the present study, photophysical and photochemical properties of curcumin (1,7-bis­(4-hydroxy-3-methoxyphenyl)-1,6-heptadiene-3,5-dione) and its seven derivatives, encompassing esterified curcumins and their bisdemethoxy conjugates have been studied computationally and experimentally to explore their suitability as photosensitizers for photodynamic therapy. We found out that enol forms of curcumins are more stable than keto ones. We observed that the main electronic levels of the curcumin derivatives well agree with the observed spectroscopic features (*i.e*. absorption, fluorescence and phosphorescence spectra). Based on the spin–orbit coupling matrix elements and the associated energy gaps, we suggested that the most plausible mechanism involves excitation from S_0_ to S_1_, followed by intersystem crossing from S_1_ to T_3_. Subsequent internal conversion occurs from T_3_ to T_2_ and T_1_, culminating in phosphorescence from T_1_ to S_0_. The computed vertical phosphorescence energy of the first triplet state (T_1_) for the studied curcumin derivatives exceed both the computed first excited-state energy of O_2_—1.06 eV in vacuum and 1.05 eV in water—and the measured value of 0.98 eV in vacuum. These findings indicate that the studied curcumin derivatives are theoretically capable of photosensitizing the production of ^1^O_2_. In this context, curcumin **HL4a** exhibits the best yield for singlet oxygen production (∼15%) and the esterification and the presence of methoxy groups poorly affect both photophysical and photochemical properties.

## Graphical abstract

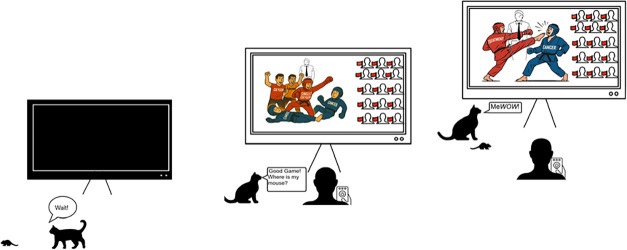

## 1 Introduction

Curcumin (1,7-bis­(4-hydroxy-3-methoxyphenyl)-1,6-heptadiene-3,5-dione) is the main natural polyphenol found in turmeric^1^ and has antioxidant,^2–4^ anti-inflammatory,^4,5^ antimutagenic,^6^ and anticancer properties.^7,1,8^ It has been shown that curcumin has direct anticancer properties as it can act as cell growth inhibitor, apoptosis inducer, and preventing metastasis.^9,10^ Indirectly, it is also used in combination with other therapies like chemotherapy,^11^ radiotherapy,^12^ immunotherapy,^13^ and photodynamic therapy (PDT).^7,14,15^ In PDT, photosensitizers are activated by light at a particular wavelength and react with oxygen in the surrounding tissue, producing reactive oxygen species that cause cell destruction in the treated area.^14,15^

Curcumin has been used as a photosensitizer in PDT to treat various cancers, including liver, oral, skin, colon, kidney, prostate and bladder, breast and cervical cancers.^7,16,17^ This photodynamic activity relies on curcumin’s ability to generate singlet oxygen (^1^O_2_) through intersystem crossing (ISC), a crucial factor in assessing its photosensitizing efficiency.^17^ Despite the promising potential, *in vitro* and *in vivo* applications, several limitations must be considered, such as poor water solubility,^18^ low bioavailability,^19^ rapid metabolism,^20^ photoinstability,^21^ chemical instability,^20^ and poor membrane permeability.^22^ To enhance solubility, stability, bioavailability and efficacy, strategies like chemical modifications,^23^ nanoformulations,^24^ water-soluble derivatives,^25^ and cyclodextrin complexes have been used.^26^ Photoinstability is managed with stabilizers, and rapid metabolism is addressed via piperine coadministration^27^ or slow-release systems such as biodegradable polymers.^28^ Also, the use of metal complexes with curcumin, including transition metal ions^29–31^ and lanthanide ions,^32–37^ can enhance stability, solubility and therapeutic efficacy.

Curcumin has a broad absorption spectrum with maximum at ∼420 nm in polar solvents, which has been assigned to a π → π* transition.^38^ The curcumin emission spectrum exhibits a Stokes shift of 2000–6000 cm^–1^ depending on the nature of the solvent.^38,39^ Various research groups have investigated the structural and photophysical properties of curcumin and its derivatives using DFT and TD-DFT. For example, Ji et al. employed DFT with the polarized continuum model (PCM).^40^ In nonpolar solvents, keto–enol tautomerism occurs such that both tautomeric forms are present. Their results indicated that the enol form is more stable than the keto form by 7.75 kcal·mol^–1^, making it the predominant species in solution. Supporting this conclusion, TD-DFT calculations revealed that the absorption maximum of the enol form (419 nm) closely matches the experimental values for curcumin (417 nm in benzene and 419 nm in chloroform). Furthermore, the high oscillator strength (*f* = 1.53) of the enol form aligns with the experimentally observed strong absorption spectrum of curcumin.^40^

Kolev et al.,^41^ by means of DFT calculations and vibrational spectroscopy, showed that curcumin predominantly adopts a stable planar enol form, both in solid state and in solution, stabilized by strong intramolecular hydrogen bonding. The less stable diketo form appears only minimally in nonpolar environments. In another study, Shen et al. investigated the triplet-state properties of curcumin in vacuum, benzene, and DMSO by means of TD-DFT calculations.^42^ Their findings showed that, in benzene and DMSO, excited curcumin can interact with O_2_ to produce ^1^O_2_ and superoxide (O_2_^–^) through energy transfer and electron transfer mechanisms. This insight provided an explanation for the experimentally observed photosensitizing properties of curcumin. In addition, they realized that the lowest T_1_ transition energy is only slightly influenced by the medium, the difference being <0.05 eV.^42^

Previous studies mentioned above have primarily focused on “native” curcumin and its natural degradation products, investigating the photosensitization mechanism mainly as a chemical phenomenon—how curcumin reacts to light. These works relied heavily on TD-DFT calculations to predict energy levels and highlighted how structural changes, such as degradation or pH variations, can drastically alter curcumin’s photophysical behavior. ^1^O_2_ generation was considered only theoretically based on energy gaps, and the triplet state was discussed in general solvent environments without detailed experimental validation.

In contrast, in this study, we expanded the scope of curcumin research by investigating parent curcumin alongside a diverse set of derivatives, including esterified curcumins and bisdemethoxy conjugates. Our primary objective was to bridge the gap between theoretical photophysics and medicinal application, specifically evaluating these compounds for their efficacy in PDT. To achieve a comprehensive understanding of their performance, we utilized a dual-methodology approach. Through computational analysis, we employed TD-DFT calculations to map vertical phosphorescence energies against the required energy threshold for oxygen activation. This was complemented by experimental validation, where we performed detailed spectroscopy to measure absorption, fluorescence, and phosphorescence, allowing us to validate the electronic states predicted by our models. The core of our investigation focused on how specific structural modifications—specifically the removal of methoxy groups and the addition of ester groups—influence the compounds’ photophysical properties and their subsequent ^1^O_2_ production yields.

## 2 Materials and Methods

### 2.1 Experimental Section

Curcumin (**HL1a**) and bisdemethoxycurcumin (**HL1b**) were purchased from TCI Europe and were used as received. All other materials were obtained from commercial sources and were used as received. IR spectra were recorded from 4000 to 600 cm^–1^ with a PerkinElmer Spectrum 100 FT-IR instrument. FT-IR spectra are presented in SI as Figures S1–S6. ^1^H, ^13^C NMR, {^1^H–^1^H}-COSY NMR, {^1^H–^13^C}-HSQC and {^1^H–^13^C}- HMBC spectra were recorded on a 500 Bruker Ascend (500.1 MHz for ^1^H and 100 MHz for ^13^C) and a 400 Mercury Plus Varian instrument (400 MHz for ^1^H and 100 MHz for ^13^C). Referencing is relative to TMS (^1^H). Coupling constants are given in Hz. Positive and negative ion electrospray ionization mass spectra (ESI-MS) were obtained on a Series 1100 MSI detector HP spectrometer using methanol or acetonitrile as the mobile phase. Solutions for analysis (3 mg mL^–1^) were prepared using reagent-grade methanol and acetonitrile. Masses and intensities were compared to those calculated using IsoPro Isotopic Abundance Simulator, version 2.1.28. Melting points were recorded on an STMP3 Stuart scientific instrument and a capillary apparatus. Samples for microanalysis were dried in vacuo to constant weight (20 °C, ca. 0.1 Torr) and analyzed with a Fisons Instruments 1108 CHNS-O elemental analyzer. UV-stability studies have been conducted with a Varian Cary spectrometer.

Absorption spectra were collected with a PerkinElmer Lambda 650 UV–vis spectrophotometer. Room-temperature luminescence was measured with a Fluorolog 3 (Horiba-Jobin Yvon) spectrofluorometer, equipped with a Xe lamp, an excitation double monochromator, a single-emission monochromator (mod. HR320), and a photomultiplier in photon counting mode for the detection of the emitted signal. All of the spectra were corrected for the spectral distortions of the setup.

The fluorescence overall quantum yield (ϕ_ovl_) has been determined by means of the secondary method in dichloromethane (DCM) solution,^43^ using the eq 1

1 $$\frac{\phi_{\mathrm{ovl}\left(x\right)}}{\phi_{\mathrm{ovl}\left(r\right)}}=\left[\frac{A_{r}\left(\lambda \right)}{A_{x}\left(\lambda \right)}\right]\times \left[\frac{n_{x}^{2}}{n_{r}^{2}}\right]\times \left[\frac{D_{x}}{D_{r}}\right]$$

where: the *x* subscript refers to sample and *r* to the standard and other symbols have the following meanings: Φ_ovl_ is quantum yield, *A* is the absorbance at the excitation wavelength, *D* is the integrated emission area across the band and *n*’s are the refractive indexes of the solvent containing the sample (*x*) and the standard (*r*), respectively, at the sodium *D* line and at the temperature of the emission measurement. Quinine sulfate (1*N* aqueous solution of sulfuric acid; λ_exc_ = 345 nm; λ_em_ = 360–640 nm) was employed as the reference. A linear relationship between the integrated emission area and the optical density has been observed for all the investigated curcumins in DCM (see Figures S7–S9).

The yield of singlet oxygen production (φ_*s*_) of the compounds was estimated by adopting the relative method, exploiting the near-IR luminescence of ^1^O_2_ (peaked at λ ≈ 1270 nm). Erythrosin B in ethanol was used as the standard. Measurements were performed on air-equilibrated solutions. ^1^O_2_ emission spectra were collected and corrected for the excitation intensity and the detector sensitivity by means of an Edinburgh Instruments FLS1000 spectrofluorometer equipped with a Xe excitation source and a near-IR PMT detector in liquid nitrogen cooled housing. Emission lifetimes were obtained with the multichannel scaling (MCS) technique, following the decay at 1270 nm after photoexcitation with a microsecond flashlamp. Lifetimes were extracted by reconvolution fit or tail fit of the experimental decay traces and the goodness of the fit was judged by the chi-squared test. Φ_*s*_ has been estimated by eq 2

2 $$\Phi_{s}=\Phi_{R}\left(\frac{I_{s}}{I_{R}}\right)\left[\frac{1-10^{\left(A_{R}\right)}}{1-10^{\left(A_{S}\right)}}\right]{\left(\frac{n_{s}}{n_{R}}\right)}^{2}\left(\frac{\tau_{R}}{\tau_{s}}\right)$$

Where Φ_*R*_ is the yield of singlet oxygen production of erythrosine B, *I* is the integrated emission intensities under the band peaked at ∼1270 nm, *A* is the absorbance of the solution, *n* is the refractive index of the solvent and τ is the observed lifetime of singlet oxygen emission. The subscripts *S* and *R* refer to the sample and the reference standard, respectively.

O_2_ emission spectra were obtained upon excitation in the Vis of the different curcumins in DCM (λ_ex_ = 402 nm) and of Erythrosin B in Ethanol (λ_ex_ = 535 nm), under the same experimental conditions (*A* ∼ 0.3–0.5).

### 2.2 Computational Details

The most stable structures of enol and keto forms of curcumin were derived from the recent work by Madinah et al.^44^ They investigated various conformers of curcumin using the DFT approach in the gas phase at APFD^76^/6–311++G­(d,p) level of theory,^45^ which provided a rationale for selecting the most stable conformer of curcumin as the starting structure in the present study. We assume that the changes in the structures of curcumin derivatives are in line with the parent molecule. Based on these structures, ground-state geometry optimizations were carried out at DFT level, employing the B3LYP^46–49^ functional in combination with 6–31++G­(d,p) basis set,^50^ including the empirical dispersion correction GD3BJ.^51^ The B3LYP functional was chosen for two main reasons: first, it is a standard method for predicting the structures and energies of organic compounds such as curcumins; second, it allows for meaningful comparison with the CAM-B3LYP functional, which will be used to model excited-state geometries and excitation energies. As a test calculation, we also optimized the ground-state geometry of curcumin, using the CAM-B3LYP functional to assess the extent to which geometry optimization, B3LYP compared to CAM-B3LYP, influences the excitation energies. We optimized geometry of curcumin at CAM-B3LYP/6–31++G­(d,p) level of theory and performed a single point calculation at TD-DFT-CAM-B3LYP/6–31++G­(d,p) level of theory, Approach II, in solvent to compare to the first three singlet and triplet excitation energies at B3LYP/6–31++G­(d,p)//TD-DFT (CAM-B3LYP/6–31++G­(d,p), Approach I).

Where needed, solvent effects were considered using the IEFPCM (Integral Equation Formalism Polarizable Continuum Model).^52^ The ground-state optimized structures of the keto and enol forms of all the studied curcumins are provided in Table S1 of the Supporting Information (SI). Excited-state properties were computed using the linear-response formalism (the default implementation in Gaussian). As a test calculation, state-specific solvation effects were additionally treated within the nonlinear-response framework to evaluate their impact on the predicted excitation energies of curcumin.

To reproduce the electronic absorption and emission spectra, single-point energy calculations were performed using the TD-DFT approach at the same level of theory applied to the ground state. These calculations were conducted on the ground-state and excited-state optimized geometries, respectively. For the excited-states calculations, CAM-B3LYP^53^ was adopted in combination with 6–31++G­(d,p) basis set, considering 10 excited-state roots, since it is well-known that it is a suitable functional for excited systems with charge transfer features.^54^ As a test calculation, another member of the range-separated density functionals, ωB97X-D,^55^ was employed to predict the excitation energies of curcumin.

In order to investigate the nature of electronic transitions, natural transition orbital (NTO)^56^ analyses were performed for the first three singlet and triplet excited states of the studied curcumins. These analyses simplify the interpretation of electron density redistribution during excitation, providing insights into the electronic structure and transition characteristics. All NTO analyses were based on vertical electronic transitions computed at the CAM-B3LYP/6–31++G­(d,p) level of theory in DCM solvent on the minimum energy isomers calculated with the protocol described above. To simulate the absorption spectrum with realistic peak broadening, a Gaussian function with a fwhm of 0.3 eV was applied, consistent with values used for the enol conformer in previous studies.^57,58^

In addition to singlet-state calculations, triplet states were also investigated, as they play a key role in assessing the ability of a potential photosensitizer to generate singlet oxygen species.^59^ All triplet-state calculations were initially carried out in DCM, the solvent used in the experimental studies. However, considering that the intended application of these curcumins is within the human body, where water is the primary environment, we examined the solvent dependence of the first triplet state by performing the same calculations in methanol (MeOH) and methylcyclohexane (MeCY), alongside DCM, focusing specifically on **HL3a**. Methanol was chosen as a polar protic solvent with hydrogen-bonding ability, making it a close analogue to water. **HL3a** selection is also justified by the highly similar structural, and thus photophysical features, as well as the close alignment of computed triplet energies among **HL3a**, **HL3b**, **HL4a**, and **HL4b**, enabling us to confidently generalize the results obtained for **HL3a** to the other curcumins. MeOH, a polar protic solvent, and MeCY, a nonpolar solvent, provide higher and lower dielectric constants compared to DCM, offering a broader perspective on solvent dependency. Furthermore, MeOH is a relevant choice for PDT applications due to its dielectric constant, although the ideal solvent would be water. However, water is not considered here due to the limited solubility of the studied compounds. In addition, spin–orbit coupling (SOC) matrix elements were computed to assess the efficiency of possible intersystem crossing pathways.

All DFT and TD-DFT calculations were performed using Gaussian 16 Rev. A.03.^60^ SOC calculations were performed using ORCA Version 6.0.^61^

## 3 Results and Discussion

### 3.1 Synthesis and Characterization

The curcuminoid ligands **HL2a**, **HL2b**, **HL3a**, **HL3b**, **HL4a**, and **HL4b** shown in Figure 1, were synthesized starting from commercially available curcumin (**HL1a**) and bisdesmethoxycurcumin (**HL1b**). Ligands **HL2a** and **HL2b** were obtained following a modified procedure based on previously reported methods.^36,62^ The ligands **HL3a** and **HL4a** were previously reported in the literature;^63^ however, in this work, the synthetic procedures and product work-ups were optimized, resulting in significantly improved reaction yields. These improvements were inspired by the synthetic route developed for **HL3b** and **HL4b**.^64^

**Figure 1:**
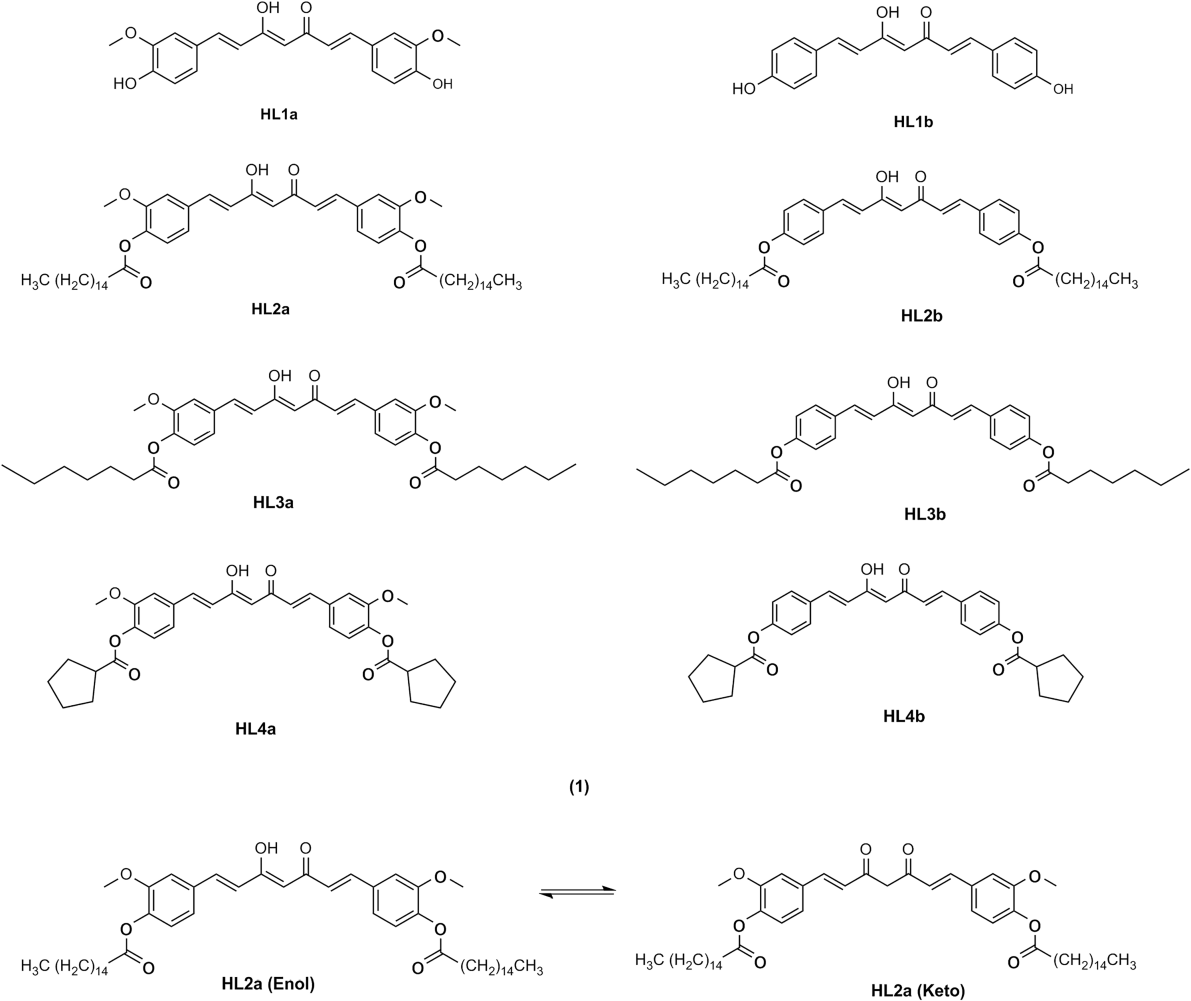
Curcumins studied in this work (1) and keto–enol equilibrium of **HL2a**, as an example (2).

In the ^1^H NMR spectra recorded in deuterated chloroform, the presence of a singlet around 5.9 ppm is attributable to a single proton of the enol form bound to an oxygen atom by means of an intramolecular hydrogen bond.

### 3.2 Structural and Electronic Properties

#### 3.2.1 Stabilities of Tautomers

The coordinates of the optimized structures, relative electronic energies, zero point vibrational energy corrected electronic energies, and Gibbs free energies (all in kcal mol^–1^) of the tautomers (k: keto and e: enol) of the studied curcumins are presented in Figures S10 and S11 in SI. The equilibrium of keto–enol tautomerism in curcumin is influenced by the solvent, pH, and temperature.^65^ In nonpolar solvents, curcumin predominantly adopts the enol form, stabilized by intramolecular hydrogen bonding, whereas in polar solvents, a partial shift to the keto form occurs.^66,67^

In previous studies, DFT calculations indicated that the enol tautomer is more stable and prevails in the gas phase and organic solvents.^42,68^ In contrast, the keto form is favored in aqueous solutions due to its stabilization through interactions with water molecules.^69^ The stabilities of the keto and enol tautomers of the studied curcumins in both the gas phase and DCM are presented in Figure 2. The curcumins **HL2a** and **HL2b** have not been studied for computational convenience, while the results are expected to be similar to **HL3a** and **HL3b**.

**Figure 2:**
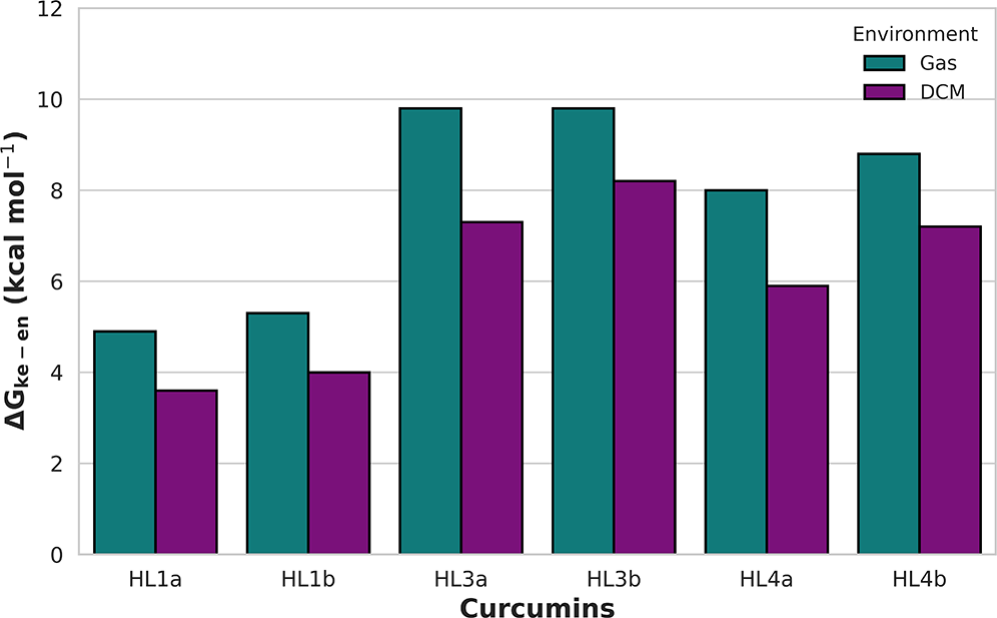
Relative stabilities of enol and keto tautomers (Δ*G*_ke‑en_ = *G*_ke_ – *G*_en_, kcal mol^–1^) of the studied curcumins in the gas phase and DCM.

In all cases, the enol tautomer is more stable than the keto form, with differences ranging from 4.9 kcal mol^–1^ (**HL1a**) to 9.8 kcal mol^–1^ (**HL3b**) in the gas phase, and from 3.6 kcal mol^–1^ (**HL1a**) to 8.4 kcal mol^–1^ (**HL3b**) in DCM. According to the Boltzmann distribution, at 300 K and with a Δ*G* of 3.6 kcal mol^–1^, the solution in DCM predominantly consists of enol tautomers (98.14%). The energy difference between the keto and enol tautomers decreases when moving from the gas phase to DCM. This indicates that, although the keto tautomer is more stabilized by the solvent than the enol form, the large stability gap established in the gas phase cannot be fully overcome by solvation effects. Furthermore, when comparing curcumins with their bisdemethoxy counterparts, it emerges that the energy gap between the keto and enol forms increases for some bisdemethoxy derivatives relative to the parent curcumins, with the differences calculated as 0.4 kcal mol^–1^ for **HL1a** and **HL1b** and 0.8 kcal mol^–1^ for **HL4a** and **HL4b**.

#### 3.2.2 Photophysical Characterization of Curcumins

##### 3.2.2.1 UV–Vis Absorption and Luminescence Spectra

From this point forward, the enol tautomer, which is largely predominant in solution, is considered for further investigation. The measured and calculated UV–vis absorption and emission spectra of curcumins **HL3a**, **HL3b**, **HL4a**, and **HL4b** in DCM are shown in Figure 3. Different overlay of measured and computed UV–vis absorption and emission spectra of the curcumins (**HL3a** and **HL3b**, **HL4a** and **HL4b**) in DCM are presented as Figure S12. The computed spectra for **HL1a** and **HL1b**, along with the measured spectra for **HL2a** and **HL2b** in DCM, are provided in Figure S13 of the SI.

**Figure 3:**
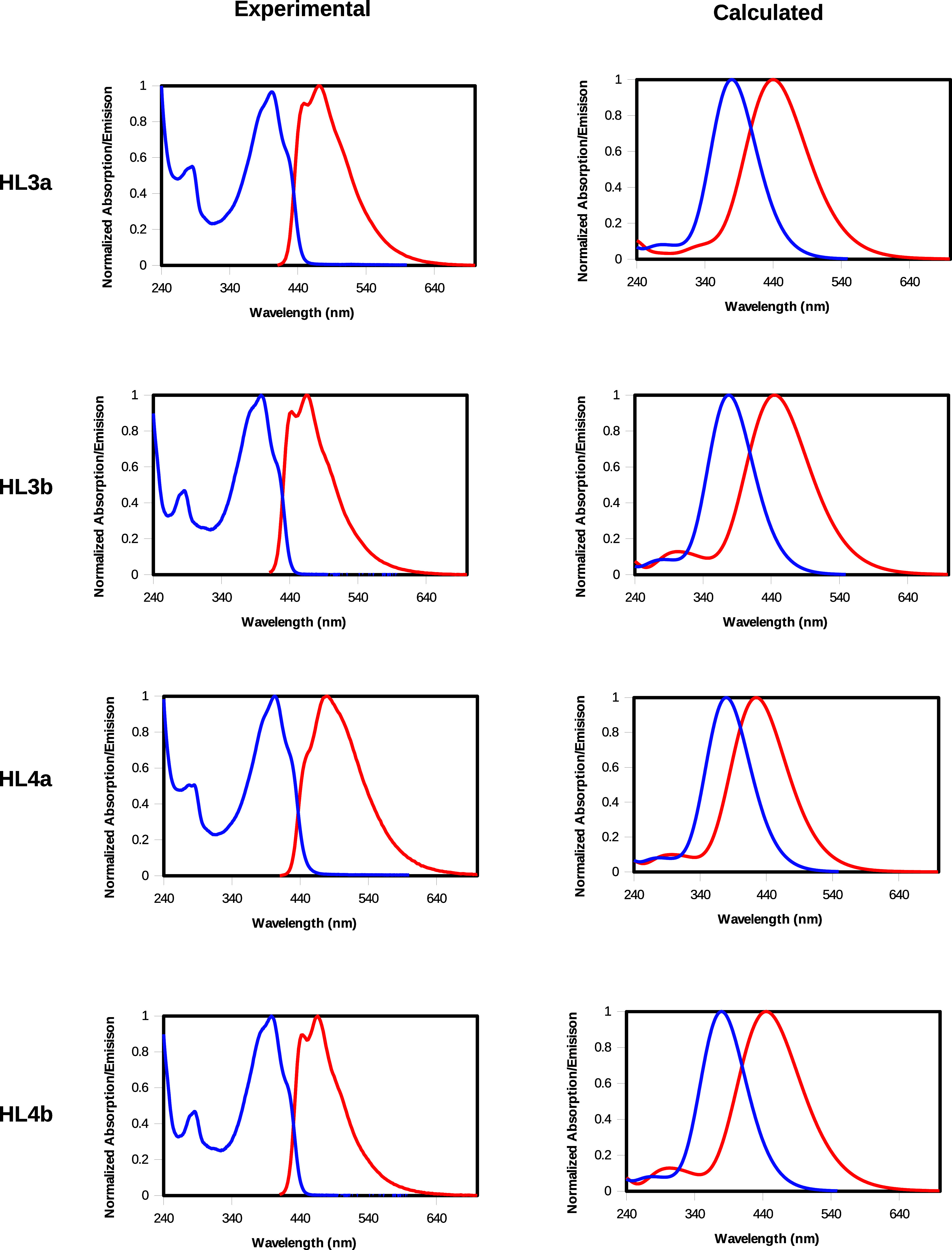
Measured and computed UV–vis absorption and emission spectra of the curcumins (**HL3a** and **HL3b**, **HL4a** and **HL4b**) in DCM while that blue and red lines are absorption and emission spectra, respectively. The corresponding computed spectra for **HL1a** and **HL1b** together with measured spectra **HL2a** and **HL2b**, are presented as Figure S7 in the SI.

The agreement between the measured and calculated absorption spectra is satisfactory (Table 1) demonstrating the suitability of the TD-DFT approach for reproducing the experimental results. In particular, vertical excitation energies to the first singlet state (VEE­(S_1_)) for the investigated curcumins in DCM (enol form) are compared with the experimental transition energies: the differences range from 0.16 to 0.19 eV, consistently with the well-known overestimation of excitation energies from CAM-B3LYP.^70^ The results also show that for parent curcumin, compared to other derivatives, the −OCH_3_ substituent has only a minor effect on the vertical excitation energy. In contrast, replacing −OH groups with ester functionalities leads to a noticeable blue shift of the VEE in the 0.06–0.16 eV range.

**Table 1:** Vertical Excitation Energies (VEE­(S1)) in eV [Corresponding Wavelengths in nm], Experimental Maximum Absorption Wavelengths (λ_max,ab_) in nm [Corresponding Transition Energies in eV], and Their Differences for the Investigated Curcumins in DCM (Enol Form)

	VEE(S_1_) (eV) [λ (nm)]	λ_max,ab_ (nm) [Δ*E*_max,ab_ (eV)]	VEE(S_1_) – Δ*E*_max,ab_ (eV)
**HL1a**	3.12 [397]	419^a^ [2.96]	0.16
**HL1b**	3.18 [389]	411^b^ [3.02]	0.16
**HL3a**	3.27 [379]	402 [3.08]	0.19
**HL3b**	3.28 [378]	398 [3.12]	0.16
**HL4a**	3.27 [380]	403 [3.08]	0.19
**HL4b**	3.28 [378]	398 [3.12]	0.16

a The data were taken from the work by Patra et. al.^71^

b To the best of our knowledge, no λ_max_ value for bisdemethoxycurcumin in DCM has been reported in the literature. Therefore, we used the value obtained in chloroform, as reported in the work Nardo et al.^72^ The other experimental data are from the present study.

##### 3.2.2.2 NTO Analysis of the Electronic Transitions of the Studied Curcumins

NTO analysis was employed to clarify the nature of the excited states. For conciseness, VEEs, oscillator strengths (OS), transition character (π → π*, *n* → π*, or mixed), contribution coefficient, and the corresponding NTO pairs (occupied and virtual) of curcumin **HL4a** are summarized in Table 2. The analogous data for the remaining curcumin derivatives are provided in Tables S2–S6 in the SI. As seen in Table 2, the first and third singlet excited states primarily exhibit a π → π* character, while the second singlet excited state is characterized by an *n* → π* transition. This assignment is further corroborated by the oscillator strength value, that is close to zero, consistently with the typical forbidden nature of *n* → π* transitions. The assigned nature of each transition listed in Table 2 is consistent with the corresponding transition characters reported in Tables S2–S6 for the parent compound and its derivatives. Replacing the hydroxy groups with ester functionalities affects the charge transfer character and, consequently, the vertical VEE. On the other hand, when comparing enol with keto forms, the substitution of specific hydrogen atoms with methoxy groups has only a minor impact on the natural transition orbitals (NTOs), which is reflected in the relatively unchanged VEEs.

**Table 2:**
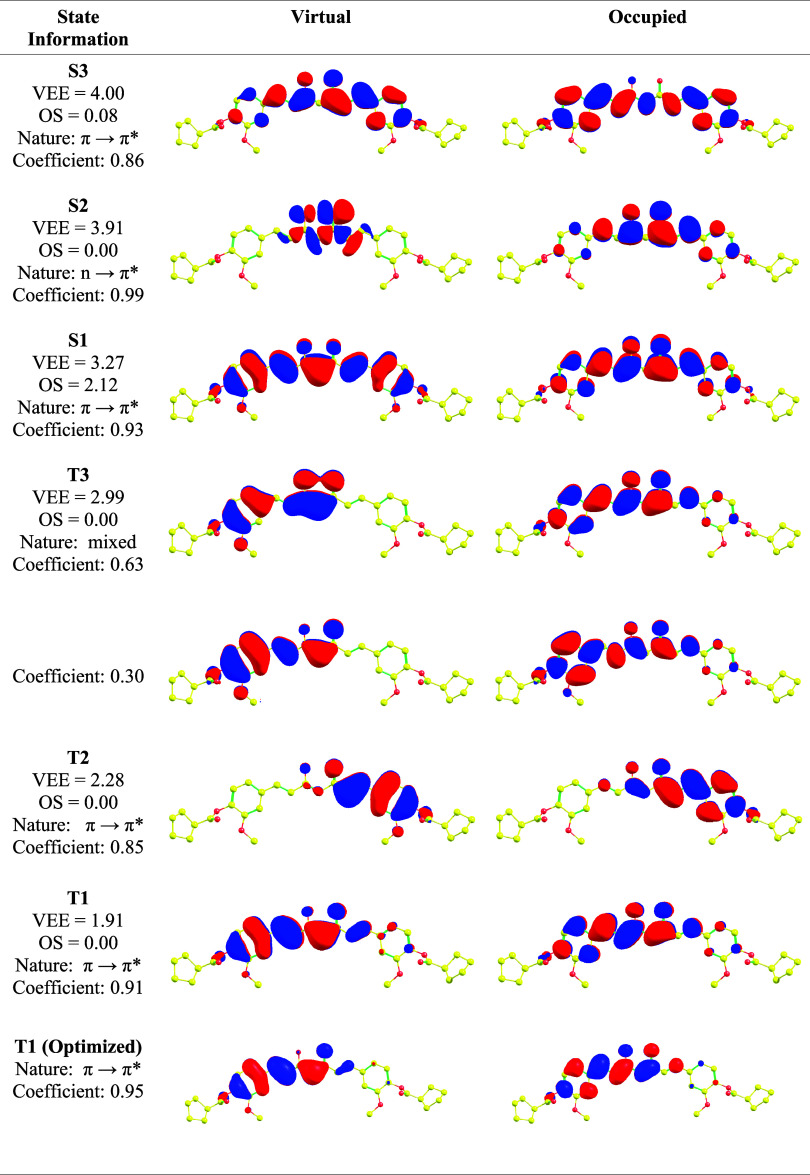
VEEs (in eV), OSs, NTOs and Natures of Transitions of HL4a for the First Singlet (S1–S3) and Triplet (T1–T3) States in DCM^a^

a Color code: Carbon (yellow), Oxygen (red. Hydrogen atoms are removed for the sake of clarity.

To evaluate the robustness of the computed excitation energies, we examined three factors: (i) ground-state geometry, (ii) choice of density functional, and (iii) solvation treatment. The influence of ground-state geometry was assessed by comparing CAM-B3LYP/6–31++G­(d,p) optimized geometries with B3LYP/6–31++G­(d,p) geometries, both followed by TD-DFT single-point calculations in solvent (Approaches II and I, respectively; Table S7). Differences in singlet and triplet excitation energies are small, averaging 0.08 and 0.10 eV, respectively, while state ordering and electronic character remain unchanged, confirming the reliability of B3LYP-optimized geometries. To test the effect of the functional, triplet excitation and phosphorescence energies were computed with ωB97X-D and compared to CAM-B3LYP (Table S8). Vertical triplet excitation energies at the ωB97X-D level are ∼0.1 eV higher, and first vertical phosphorescence energies, VPE­(T1), differ by at most 0.09 eV using single-point calculations, or 0.07 eV when the T_1_ state is fully optimized. These small differences indicate that both functionals provide consistent descriptions of triplet energies and confirm curcumin’s ability to produce singlet oxygen. Finally, the effect of state-specific (nonlinear-response) solvation on triplet energies was tested (Table S9), showing no significant deviation from the linear-response results. Given the structural similarity among the curcumins, these findings can be generalized to the entire series, confirming the overall reliability of the computed excitation energies.

A similar trend is also observed for the differences between vertical emission energies (VEM­(S1), excited-state geometry) and experimental values (Table 3), though with larger discrepancies ranging from 0.12 to 0.34 eV. These greater deviations likely stem from the lower accuracy of excited-state geometries, as indicated by Dorbeej et al.^73^ Finally, observed Stokes shift (in the 0.36–0.49 eV range) for the studied curcumins, is in good agreement with the calculated one (0.30–0.50 eV range).

**Table 3:** Vertical Emission Energies (VEM­(S1)) in eV [Corresponding Wavelengths in nm], Experimental Maximum Emission Wavelengths (λ_max,em_) in nm [Corresponding Transition Energies in eV], and Their Differences for the Investigated Curcumins in DCM (Enol Form)

curcumin	VEM(S_1_) (eV) [λ (nm)]	λ_max,em_ (nm) [Δ*E*_max,em_ (eV)]	VEM(S_1_) – Δ*E*_max,em_ (eV)
**HL1a**	2.82 [440]	477^a^ [2.60]	0.22
**HL1b**	2.86 [433]	486^b^ [2.55]	0.31
**HL3a**	2.92 [425]	470 [2.64]	0.28
**HL3b**	2.79 [445]	464 [2.67]	0.12
**HL4a**	2.92 [425]	480 [2.58]	0.34
**HL4b**	2.78 [445]	466 [2.66]	0.12

a The data were taken from the work by Patra et al.^71^

b To the best of our knowledge, no λ_max_ value for bisdemethoxycurcumin in DCM has been reported in the literature. Therefore, we used the value obtained in chloroform, as reported in the work by Nardo et al.^72^ The other experimental data are from the present study.

##### 3.2.2.3 Phosphorescence and Quantum Yield Measurements

We also estimate fluorescence overall quantum yield (ϕ_ovl_) for the curcumins under investigation (see Section 2 for more details). In all cases, these yields are not higher than 2%, pointing out a low emission efficiency of these molecules (Table 4), probably connected with the presence of nonradiative processes depopulating the emitting single excited state. Among these, we can list: (i) ISC process, feeding the triplet states; (ii) cis trans isomerism and (iii) intramolecular proton transfer

**Table 4:** Experimentally Determined Overall Fluorescence Quantum Yield (Φ_ovl_), Singlet Oxygen Yield (Φ^1^O_2_), and Singlet Oxygen Emission Lifetimes (τ^1^O_2_) for the Curcumins under Investigation Dissolved in DCM^a^

	Φ_ovl_ (%)	Φ^1^O_2_ (%)	τ^1^o_O2_ (μs)
**HL2a**	2	13	93
**HL2b**	1	10	93
**HL3a**	2	11	93
**HL3b**	1	8	93
**HL4a**	2	14	93
**HL4b**	1	10	93

a The standard used for Φ^1^O_2_ determination was Erythrosin B in EtOH (Φ^1^O_2_ = 69%, τ_R_ = 14 μs).

Furthermore, phosphorescence spectra of curcumin **HL2a** (chosen as representative molecule) have been collected at 77 K in EtOH/MeOH and MeCy. In both cases, in particular for the sample dissolved in MeCy, the presence of several peaks in the 400–700 range, is observed (Figure 4).

**Figure 4:**
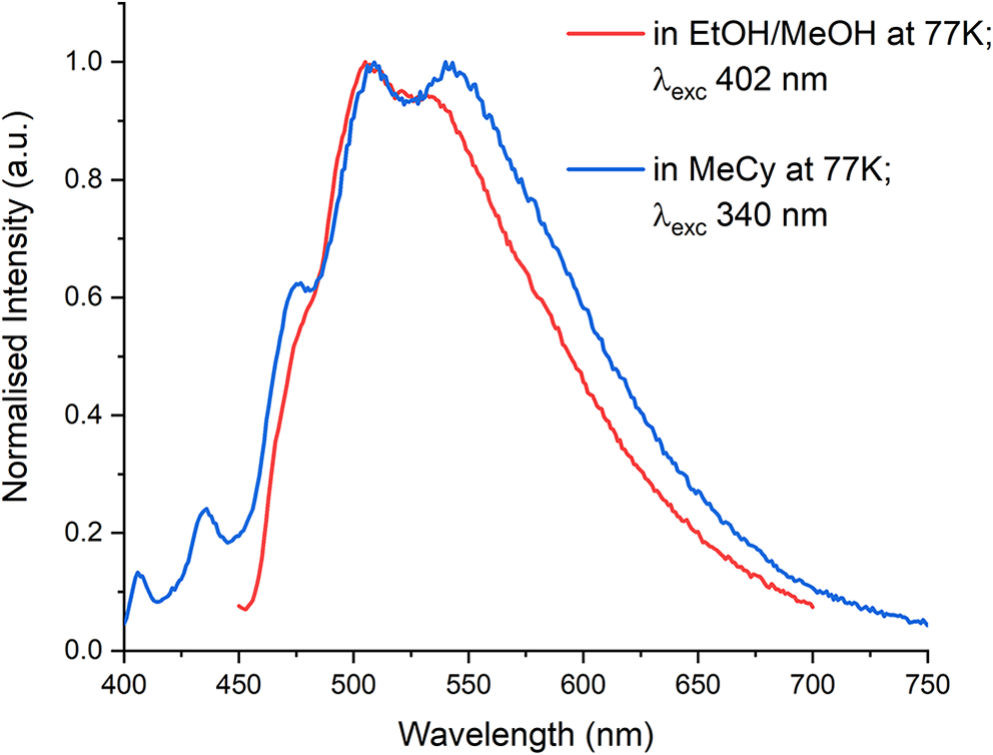
Phosphorescence spectra of curcumin **HL2a** in EtOH/MeOH and in methylcyclohexane.

The calculation of VEE for triplet states in the case of **HL3a** (a less computational demanding analog of **HL2a**) reveals that five triplet states are possible: T_1_ [1.91 eV (649 nm)]; T_2_ [2.28 eV (543 nm)]; T_3_ [2.99 (415 nm)]; T_4_ [3.59 (345 nm)] and T_5_ [3.61 eV (343 nm)]. Considering the usual Stokes shift of the phosphorescence band with respect to VEE for triplet states, the two wavelength ranges for experimental phosphorescence (400–750 nm) and for computed VEE­(T*_n_*) (343–649 nm) are in very good agreement.

To corroborate the partially forbidden nature of the aforementioned emission peaks, we measured the 77 K luminescence decay of **HL2a** at 580 nm in EtOH/MeOH, upon excitation at 400 nm. The estimated averaged lifetime was around 4 μs, compatible with emission from triplet states (phosphorescence).

##### 3.2.2.4 Singlet Oxygen Generation

The suitability of the studied curcumins for PDT applications was assessed. The computed VEEs of the first triplet state (T_1_) for the studied curcumin derivatives (for the dominant enol tautomer) suggest that they can, in principle, sensitize the generation of ^1^O_2_. Specifically, all the computed VEE­(T_1_) values (Tables 2 and S2–S6) exceeds both the computed (1.06 eV)^74^ and measured (0.98 eV)^75^ first excited-state energy of O_2_ in vacuum. In water, the computed value remains similar to that in vacuum, at approximately 1.05 eV.^74^ These findings suggest that the studied curcumin derivatives possess the potential to generate ^1^O_2_, reinforcing their viability as photosensitizers for PDT. Accordingly, we experimentally estimated the yield of singlet oxygen production, upon evaluation of the emission peak of ^1^O_2_ in the NIR spectral region at about 1270 nm (see Section 2 and Figure S14 for details). For the curcumin molecules under investigation the Φ^1^O_2_ lies in the 8–14% range with τ^1^O_2_ equal to 93 μs (Table 4). Figure 5 illustrates the Jablonski diagram for **HL4a**, chosen as representative, in DCM and its role in ^1^O_2_ production, relevant for PDT application. The corresponding Jablonski diagram for other curcumins are presented as Figures S15–S17 in the SI. Jablonski diagram for **HL4a** in MeCY and MeOH are presented in the SI.

**Figure 5:**
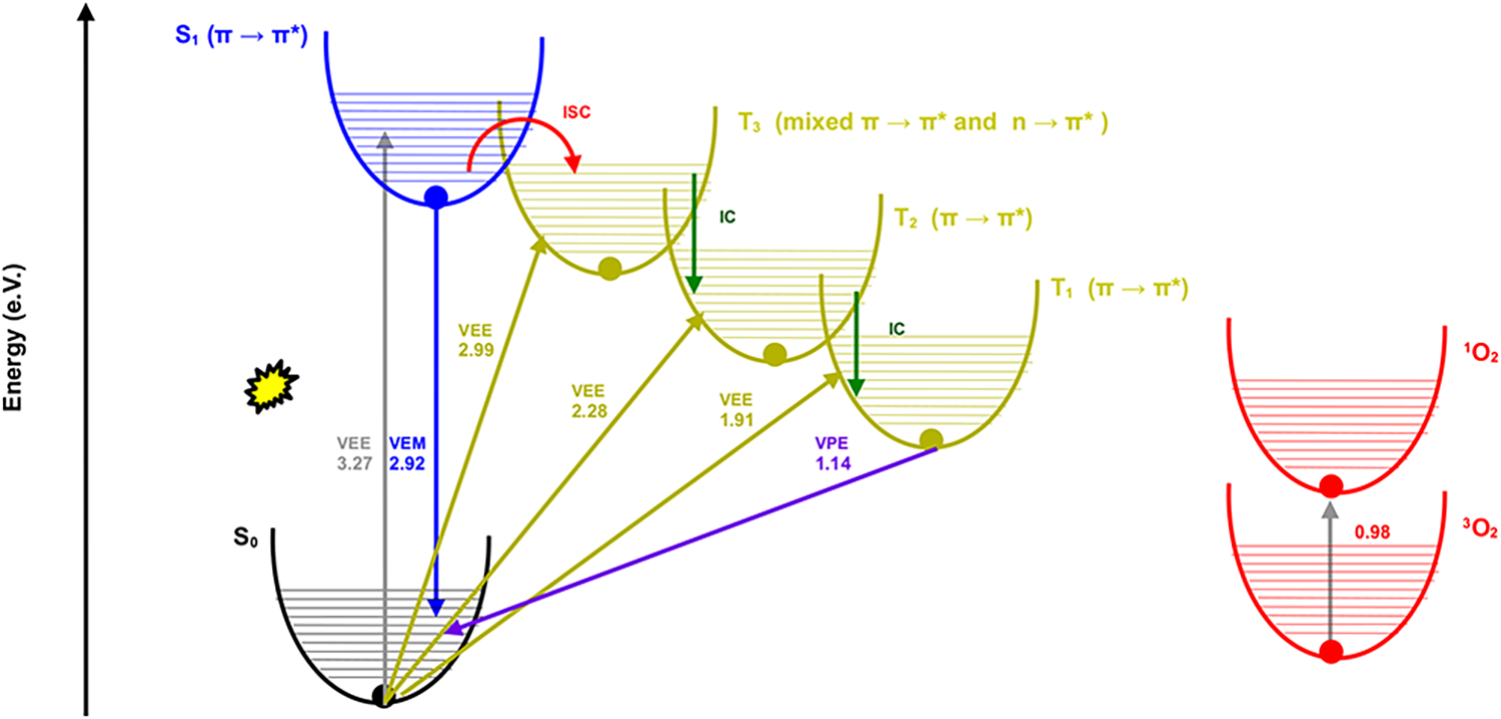
Jablonski diagram for **HL4a** in DCM with emphasis on triplet states and singlet oxygen production. VEE, VEM, ISC, IC, and VPE are reported in eV and stand for Vertical Excitation Energy, Vertical Emission Energy, Inter-System Crossing, Internal Conversion, and Vertical Phosphorescence Energy, respectively. All values, except for the excitation energy of the oxygen molecule, which is measured in gas phase and taken from the work,^73^ are computed in the present study.

The process begins with an initial transition from the ground state (S_0_) to the first singlet excited state (S_1_) of **HL4a**, with a VEE of 3.27 eV (379 nm), characterized by a π → π* transition. The first five triplet excitation energies are 1.91 eV (650 nm), 2.28 eV (543 nm), 2.99 eV (415 nm), 3.59 eV (345 nm), and 3.61 eV (343 nm). For efficient ISC, based on El-Sayed’s rule,^76^ transitions typically occur between states that are close in energy and differ in orbital characters. From S_1_ (π → π* nature) at 3.27 eV, the most probable ISC occurs to T_3_ (2.99 eV), as T_3_ is the nearest lower-energy triplet state, exhibiting mixed character (*n* → π* and π → π*). The next triplet states (T_4_ and T_5_) lie slightly above S_1_, making ISC to these states less likely due to the energy gap inversion. Following ISC from S_1_ to T_3_, internal conversion (IC) sequentially progresses from T_3_ to T_2_ and subsequently from T_2_ to T_1_. T_1_ is in a good energy position to react with ^3^O_2_ to produce ^1^O_2_. To assess the extent to which intersystem ISC is driven by spin–orbit coupling (SOC) and to validate the proposed mechanism, we performed SOC analysis. As SOC cannot be computed with Gaussian TD-DFT, these calculations were carried out using ORCA. Although a fully rigorous comparison between the two software packages would require a benchmark, we consider the comparison sufficient to evaluate the relevance of SOC for curcumins. Table S10 reports the first three computed singlet and triplet excitation energies of curcumin in DCM at the B3LYP/6–31++G­(d,p)//TD-DFT CAM-B3LYP/6–31++G­(d,p) level with both Gaussian (Full TD-DFT and TDA-TD-DFT) and ORCA (TDA-TD-DFT), along with the corresponding energy differences. On average, the differences are 0.03 eV for singlets and 0.01 eV for triplets, indicating excellent agreement and confirming the reliability of the ORCA calculations. Using these geometries and energies, SOC matrix elements (H_X, H_Y, H_Z, and total H­(SOC)) were computed for the first few singlet (S_n_, *n* = 0–3) and triplet (T_n_, *n* = 1–3) states (Table S11). As expected for organic molecules composed exclusively of light atoms (C, H, and O), spin–orbit coupling (SOC)–driven intersystem crossing (ISC) from S_1_ to T_1_–T_3_ is negligible, with SOC matrix elements of 0.02, 0.24, and 0.31, respectively. Nevertheless, these values qualitatively indicate that ISC between S_1_ and T_3_ is more favorable than for T_1_ and T_2_, supporting the proposed mechanism. This behavior can be rationalized by the different electronic characters of the involved states, as SOC is enhanced when singlet and triplet states differ in orbital nature. In the present case, S_1_ has predominantly π → π* character, whereas T_3_ exhibits a mixed π → π* and *n* → π* character, facilitating intersystem crossing. This assignment is further supported by the small energy gap between S_1_ and T_3_, estimated to be 0.01 eV at the TDA level and 0.12 eV using full TD-DFT. Owing to this near degeneracy, the ISC process is likely governed primarily by vibronic coupling rather than by spin–orbit interactions. In contrast, larger SOC values are observed for S_3_ → T_n_ transitions, suggesting that S_3_ could provide an alternative pathway for triplet population; however, the large energy gap between S_3_ and T_1_–T_3_ limits the significance of this channel. Overall, these findings confirm that including SOC does not alter the proposed photophysical mechanism: triplet-excited curcumin can efficiently transfer energy to molecular oxygen, enabling singlet oxygen generation.

This is supported by the vertical phosphorescence energy (VPE) of **HL4a** in DCM, which is 1.14 eV—higher than the excitation energy of the oxygen molecule in the gas phase (0.98 eV). However, the environments in these two cases—curcumin in DCM and oxygen in the gas phase—do not exactly replicate the experimental conditions, where both species were present in DCM. Nonetheless, given the relatively small difference in dielectric constant between DCM (ε = 8.93) and the gas phase (ε = 1), this comparison remains valid and should not be misleading.^77^ On one hand, although water would be the most relevant solvent for biological applications, it is not suitable here due to solubility issues and fluorescence quenching. On the other hand, previous studies have shown that the triplet energies of curcumin are only minimally influenced by the solvent. For example, Shen^42^ reported vertical excitation energies (VEE) of curcumin as 1.95 eV in vacuum, 1.91 eV in benzene, and 1.90 eV in DMSO. Similarly, the computed first excited-state energy of O_2_ is 1.06 eV in vacuum and 1.05 eV in water, while the experimental value in vacuum is 0.98 eV—all in close agreement. In our study as well, the triplet energies show minimal sensitivity to solvent effects, as illustrated in Figure S18, which presents the Jablonski diagrams for **HL3a** in MeCY and MeOH.

## 4 Conclusions

In this work, several derivatives of curcumin have been studied experimentally and computationally to investigate their structural and photophysical properties, as well as their suitability for PDT applications. In particular, our study can be considered one of the few contributions in which the impact of precise chemical modification of the original curcumin molecule on both photophysical and photochemical properties has been considered. Interestingly, we demonstrate that the esterification of the OH groups in 4-position and the presence or absence of the –OCH_3_ substituents in 3-position do not alter significantly either the yield for singlet oxygen production or the photophysical properties, such as fluorescence quantum yield. Using DFT/TD-DFT methods and NTOs, we found that the enol tautomer is more stable than the keto form, and the main contributions to the absorption and emission spectra arise from π to π* transitions of the former. The experimental photophysical data are in good agreement with the computational ones underlining the goodness of our combined approach. The mechanism proposed in this work involves excitation from S_0_ to S_1_, followed by intersystem crossing from S_1_ to T_3_. Subsequent internal conversion from T_3_ to T_2_ and T_1_ levels occurs, whose energy positions (in particular the one of T1) are suitable for energy transfer to molecular oxygen, promoting triplet oxygen to its singlet state, as required for PDT.

Experimentally, low fluorescence quantum yields (1–2%) suggest the presence of nonradiative channels deactivating the emitting single excited state. Interestingly, our calculation finds five possible triplet states, three of which involved in both phosphorescence emission (at 77 K) and singlet oxygen production with a moderate yield (up to 14% in the case of curcumin **HL4a**). One strategy to enhance the singlet oxygen generation of curcumin derivatives involves synthesizing metal complexes, particularly trivalent lanthanide complexes, a direction currently being pursued in our laboratories.

## Supplementary Material



## Notes

Comprehensive experimental and computational data; experimental characterization includes FT-IR spectra for all curcumin derivatives (HL2a–HL4b) and singlet oxygen (^1^O_2_) emission spectra; photophysical studies are supported by plots showing the linear relationship between integrated emission area and optical density for quantum yield determination, alongside an overlay of experimental and computed UV–vis absorption and emission spectra; computational data encompasses relative electronic and Gibbs free energies for keto–enol tautomers in both gas phase and dichloromethane (DCM); detailed Jablonski diagrams illustrate the excited-state processes, specifically intersystem crossing (ISC) and singlet oxygen production mechanisms; tabulated data includes Cartesian coordinates for all optimized structures, vertical excitation energies (VEEs), oscillator strengths (OSs), and natural transition orbitals (NTOs); finally, the SI details the methodological benchmarking of various density functionals (B3LYP, CAM-B3LYP, ωB97XD), TDA approximations, and spin–orbit coupling (SOC) matrix elements (PDF)
