# Supplementary material for: Photophysical and Photochemical Properties of a Curcumins Family: A Combined Computational and Experimental Investigation
Source: ACS Omega. 2026 Feb 4;11(6):10038–49. doi: 10.1021/acsomega.5c10926 (PMC12917661; doi:10.1021/acsomega.5c10926)
Supplement: Supplementary file 1 [file ao5c10926_si_001.pdf]

Supporting Information

for

the article

Photophysical and Photochemical Properties of a Curcumins Family: a Combined Computational and Experimental Investigation

Ali Ghiami-Shomami[1], Silvia Ruggieri[2], Silvia Mizzoni[2], Fabio Piccinelli[2*], Francesca Terenziani[3], Riccardo Pettinari[4], Noemi Pagliaricci[4], Sara Pagliaricci[4], and Andrea Melchior[1]*

[1]Polytechnic Department of Engineering, Chemical Technologies Laboratories, University of Udine, Via Cotonificio 108, 33100 Udine, Italy

[2]Luminescent Materials Laboratory, DB, University of Verona, and INSTM, UdR Verona, Strada Le Grazie 15, 37134 Verona, Italy

[3]Department of Chemistry, Life Sciences and Environmental Sustainability, University of Parma, Parco Area delle Scienze 17/a, 43124 Parma, Italy

[4]School of Pharmacy, University of Camerino, via Madonna delle Carceri, 62032 Camerino, MC, Italy

Table of Figures

Figure S1: FT- IR Spectrum of HL2a. 8
Figure S2: FT- IR Spectrum of HL2b. 14
Figure S3: FT- IR Spectrum of HL3a. 17
Figure S4: FT- IR Spectrum of HL3b. 19
Figure S5: FT- IR Spectrum of HL4a. 21
Figure S6: FT- IR Spectrum of HL4b. 23
Figure S7: Linear relationship between the integrated emission area and the optical density of curcumins HL2a and HL2b in DCM. The same relationship is reported for the reference quinine sulfate (Std; 1N aqueous solution of sulfuric acid). λ_{exc} = 345 nm. 25
Figure S8: Linear relationship between the integrated emission area and the optical density of curcumins HL3a and HL3b in DCM. The same relationship is reported for the reference quinine sulfate (Std; 1N aqueous solution of sulfuric acid). λ_{exc} = 345 nm. 26
Figure S9: Linear relationship between the integrated emission area and the optical density of curcumins HL4a and HL4b in DCM. The same relationship is reported for the reference quinine sulfate (Std; 1N aqueous solution of sulfuric acid). λ_{exc} = 345 nm. 27
Figure S10: Relative electronic energies, zero point vibrational energy corrected electronic energies, and Gibbs free energies (all in kcal/mol) of the tautomers (k: keto and e: enol) of the studied curcumins computed at B3LYP/6-31++G(d,p) level of theory in the gas phase....29
Figure S11: Relative electronic energies, zero point vibrational energy corrected electronic energies, and Gibbs free energies (all in kcal/mol) of the tautomers (k: keto and e: enol) of the studied curcumins computed at B3LYP/6-31++G(d,p) level of theory in DCM....30
Figure S12: Overlay of measured and computed UV-Vis absorption and emission spectra of the curcumins (HL3a and HL3b, HL4a and HL4b) in DCM. 32
Figure S13: Computed absorption and emission spectra for HL1a and HL1b together with measured spectra HL2a and HL2b in DCM. 33
Figure S14: $^1O_2$ emission spectra obtained upon excitation of the different curcumins in the Vis (λ_{exc} = 402 nm) in DCM and of erythrosin B (λ_{exc} = 535nm) in ethanol, collected under the same experimental conditions (Abs ~ 0.3-0.5) and normalized for the corresponding absorbance. 34
Figure S15: Jablonski diagram for HL1a (top) and HL1b (bottom) in DCM with emphasis on triplet states and singlet oxygen production. All values, except for the excitation energy of the oxygen molecule, are computed in the present study. The mechanism for singlet oxygen generation is indicated by the gray arrows. 35
Figure S16: Jablonski diagram for HL3a (up) and HL3b (down) in DCM with emphasis on triplet states and singlet oxygen production. All values, except for the excitation energy of the oxygen molecule, are computed in the present study. The mechanism for singlet oxygen generation is indicated by the gray arrows. 36
Figure S17: Jablonski diagram for HL4b in DCM with emphasis on triplet states and singlet oxygen production. All values, except for the excitation energy of the oxygen molecule, are computed in the present study. The mechanism for singlet oxygen generation is indicated by the gray arrows. 37
Figure S18: Jablonski diagram for HL3a in MeCY (up) and MeOH (down) with emphasis on triplet states and singlet oxygen production. The corresponding diagrams for the other curcumin derivatives

are included in the SI. VEE, VEM, ISC, IC, and VPE stand for Vertical Excitation Energy, Vertical Emission Energy, Inter-System Crossing, Internal Conversion, and Vertical Phosphorescence Energy, respectively. 38

Index of Tables

Table S1: XYZ coordinates (in Å) of the optimized structures of the studied species (proligand and ligands) of the all studied curcumins computed at B3LYP/6-31++G(d,p) level of theory applying dispersion correction GD3BJ in dichlormethane. 8
Table S2: VEEs (in eV), OSs, NTOs, and natures of transitions of HL1a for the first singlet (S1-S3) and triplet (T1-T3) states in DCM (color code: Carbon (yellow) and Oxygen (red)). Hydrogen atoms are removed for the sake of clarity. 14
Table S3: VEEs (in eV), OSs, NTOs, and natures of transitions of HL1b for the first singlet (S1-S3) and triplet (T1-T3) states in DCM (color code: Carbon (yellow) and Oxygen (red) Hydrogen atoms are removed for the sake of clarity. 17
Table S4: VEEs (in eV), OSs, NTOs, and natures of transitions of HL3a for the first singlet (S1-S3) and triplet (T1-T3) states in DCM (color code: Carbon (yellow) and Oxygen (red)). Hydrogen atoms are removed for the sake of clarity. 19
Table S5: VEEs (in eV), OSs, NTOs, and natures of transitions of HL3b for the first singlet (S1-S3) and triplet (T1-T3) states in DCM (color code: Carbon (yellow) and Oxygen (red) Hydrogen atoms are removed for the sake of clarity. 21
Table S6: VEEs (in eV), OSs, NTOs, and natures of transitions of HL4b for the first singlet (S1-S3) and triplet (T1-T3) states in DCM (color code: Carbon (yellow) and Oxygen (red)). Hydrogen atoms are removed for the sake of clarity. 23
Table S7: Computed excitation energies (in eV) and wavelengths (in nm) of curcumin for the lowest singlet and triplet states in DCM using B3LYP/6-31++G(d,p)//TD-DFT(CAM-B3LYP/6-31++G(d,p)) (Approach I) and CAM-B3LYP/6-31++G(d,p)//TD-DFT(CAM-B3LYP/6-31++G(d,p)) (Approach II), together with the corresponding energy differences. 25
Table S8: The computed triplet excitation energies of curcumin in DCM using CAM-B3LYP and ωB97XD density functionals. 26
Table S9: Comparison of linear-response (LR) and corrected linear-response (Corrected LR) effects on the triplet energy calculations of curcumin in dichloromethane. 28
Table S10: First three computed singlet and triplet excitation energies (in eV.) of curcumin in DCM at the B3LYP/6-31++G(d,p)//TD-DFT CAM-B3LYP/6-31++G(d,p) with Gaussian (Full DFT and TDA) and ORCA (TDA), together with the corresponding energy differences. 29
Table S11: Computed SOC matrix elements of curcumin (HX, HY, and HZ) and H (SOC) in cm^{-1}. 30

Experimental section:
Synthesis and characterization of the studied curcumins
The compounds HL2a, HL2b, HL3a, HL3b and HL4a and HL4b have been previously reported; however, comprehensive spectroscopic and analytical characterization was not available in the literature. Therefore, a detailed characterization is provided here.

HL2a. To a solution of curcumin (300 mg, 0.81 mmol) in dry pyridine (30 mL), palmitoyl chloride (0.54 mL, 1.86 mmol) and DMAP (200 mg) at 0°C were added. The reaction mixture was stirred at room temperature overnight. Thin layer chromatography displayed the disappearance of curcumin and the formation of a faster running yellow spot. Chilled water (15 mL) was added and stirred for 10 min, then the mixture was evaporated to dryness under vacuum. The residue was dissolved in dichloromethane (20 mL) and washed with water (2 x 20 mL). The organic phase was dried over Na_2SO_4, filtered and the yellow solution evaporated to dryness to afford a yellow residue. Crystallization by ethanol/water gave HL2a as yellow powder (yield 85%). It is soluble in DMSO, chlorinated solvents, acetone, and n-hexane; it is slightly soluble in alcohols, ethers, CH_3CN and DMF and it is insoluble in H2O. Anal. Calcd. for $C_{53}H_{80}O_8$: C, 75.32; H, 9.54. Found: C, 75.13; H, 9.62. m.p.: 90-92 °C. IR (cm^{-1}): 2917 vs, 2850 vs ν(aliphatic C-H); 1764 s ν(-OC=O); 1703 m and 1624 m ν(C=O), 1598 m, 1513 s, 1470 s ν(C=C).1H-NMR (DMSO-d^6, 293 K): δ 0.84 (t, 6H, C(26-26')H), 1.25 (mbr, 44H, aliphatic chain), 1.38 (m, 4H, C(14-14')H), 1.65 (m, 4H, C(13-13')H), 2.56 (m, 4H, C(12-12')H), 3.84 (s, 6H, OCH3), 6.21 (s, 2H, C(1)H), 6.98 (d, 2H, C(3-3')H, 3J = 16 Hz), 7.14 (d, 4H, C(9-9')H, ^3J = 8.0 Hz), 7.33 (d, 4H, C(10-10')H, ^3J = 8.0 Hz), 7.51 (s, 2H, C(6-6')H), 7.65 (d 2H, C(4-4')H, ^3J = 16 Hz,). ^{1}H-NMR ($CDCl_3$, 293 K): δ 0.90 (t, 6H,C(26-26')H), 1.28 (mbr, 44H, aliphatic chain), 1.45 (m, 4H, C(14-14')H), 1.65 (m, 4H, C(13-13')H), 2.61 (t, 4H, C(12-12')H), 3.90 (s, 6H, OCH_3), 5.88 (s, 2H, C(1)H), 6.59 (d, 2H, C(3-3')H, ^3J = 16 Hz), 7.08 (d, 4H, C(9-9')H, 3J = 8.0 Hz), 7.14 (s, 2H, C(6-6')H), 7.19 (d, 4H, C(10-10')H, ^3J = 8.0 Hz), 7.65 (d 2H, C(4-4')H, ^3J = 16 Hz,). ^{13}C{1H}-NMR (CDCl3): δ 14.09 [s, C(26-26')], 22.68 [C(13-13')], 25.01 [C(14-14')], 29.06, 29.27, 29.35, 29.50, 29.61, 29.65, 29.69 [from C15 to C25], 33.21, 34.05 [s, C(12-12')], 55.92 [s, OCH_3], 101.70 [s, C1] 111.51 [s, C(6-6')], 121.09 [s, C(10-10')], 123.34 [s, (C(9-9')], 124.22 [s, C(3-3')], 133.85 [s, C(5-5')], 140.02 [s, C(4-4')], 141.54 [s, C(8-8')], 151.51 [s, C(7-7')], 171.63 [s, C(11-11')], 183.12 [s, C(2-2')=O]. ESI-MS (-) CH_3CN (m/z [relative intensity, %]): 843 $[L2a]^-$.

HL2b. The ligand HL2b was synthesized as reported for HL2a starting from bisdemethoxycurcumin. HL2b was obtained as yellow powder, yield 74%. It is soluble in chlorinated solvents and DMF; slightly soluble in acetone, ethers and n-hexane and insoluble in H_2O, alcohols, DMSO and CH3CN. Anal. Calcd. For $C_{51}H_{76}O_6$: C, 78.02; H, 9.76. Found: C, 77.74; H, 9.79. m.p. 138-139°C. IR (cm^{-1}): 2916 vs, 2849 vs ν(aliphatic C-H); 1747 s ν(-OC=O), 1701 m and 1647 m ν(C=O), 1598 m, 1508 m, 1463 m ν(C=C).^{1}H-NMR ($CDCl_3$, 293 K): δ 0.91 (t, 6H, C(26-26')H), 1.29 (mbr, 44H, aliphatic chain), 1.44 (m, 4H, C(14-14')H), 1.78 (m, 4H, C(13-13')H), 2.59 (t, 4H, (C(12-12')H), 5.86 (s, 2H, C(1)H), 6.61 (d, 2H, C(3-3')H, ^{3}J = 15.90 Hz), 7.2 (d, 4H, C(9-9')H and C(7-7')H, ^{3}J = 8.5 Hz), 7.60 (d, 4H, C(10-10')H and C(6-6')H, ^{3}J = 8.5 Hz), 7.67 (d, 2H, C(4-4')H, ^{3}J = 15.90 Hz). ^{13}C{1H}-NMR ($CDCl_3$): δ 14.11[s, C(26-26')], 22.69 [s, C(13-13')], 24.80 [s, C(14-14')], 29.10, 29.25, 29.36, 29.45, 29.59, 29.64, 29.66, 29.69 (from C15 to C25), 31.93, 34.43 [s, C(12-12')], 101.84 (s, C1), 122.18 [C(9-91) and C(7-7')], 124.12 [C(3-3')], 129.19 [C(10-10') and C(6-6'), 132.61 [C(5-5')], 139.61 [C(4-4')], 152.13 [C(8-8')], 172.02 [C(11-11')], 183.17 [C(2-2')=O]. ESI-MS (-) CH_3CN (m/z [relative intensity, %]): 783 $[L2b]^-$.

HL3a. To a solution of curcumin (300 mg, 0.81 mmol) in acetone (9 mL), was mixed triethylamine (TEA 0.34 mL, 2.43 mmol) and then, after 15 minutes, heptanoyl chloride (0.38 mL, 2,43 mmol) was added at 0°C under N_2 atmosphere. The reaction mixture was stirred at room temperature. Thin layer chromatography (7:3 hexane/ethylacetate) displayed the disappearance of curcumin and the formation of a faster running yellow spot after 4 hours. Evaporation of the solvent under reduced pressure provided a yellow solid which was then purified by crystallisation with dichloromethane/ ethanol giving HL3a as yellow powder (yield 74%). The ligand is soluble in DMSO, acetone and chlorinated solvents, slightly soluble in alcohols and CH_3CN and insoluble in H_2O and n-hexane. Anal. Calcd for $C_{35}H_{44}O_8$: C, 70.92; H, 7.48. Found: C, 70.76; H, 7.56. m.p.: 105-106°C. IR (cm−1): 2932 m, 2874 w, 2856 w ν(aliphatic C–H); 1766 m ν(–OC=O), 1627m ν(C=O); 1595 m, 1583 m, 1556 m, 1511 s, 1464 m, 1450 m,1410 m ν(C=C); 1380 m, 1300 s, 1255 s, 1233 m, 1204 m, 1162 m, 1132 s, 1118 vs, 1108 vs, 1026 s, 975 s, 940 s, 915 m, 855 s, 824 m, 794 m, 776 m, 723 m, 605 m. ^{1}H-NMR ($CDCl_3$, 293 K): δ 0.94 [t, 6H, C(17-17')H], 1.37 [m, 8H, C(16-16')H and C(15,15')H], 1.46 [m, 4H, C(14-14')H], 1.79 [m, 4H, C(13–13′)H], 2.61 [t, 4H, C(12-12')H], 3.90 [s, 6H,-OCH_3], 5.88 [s, 1H, C(1)H], 6.59 [d, 2H, C(3–3′)H, 3 J = 16 Hz], 7.07 [d, 2H, C(9–9′)H, 3 J = 8.0 Hz], 7.14 [s, 2H, C(6–6′)H], 7.18 [d, 2H, C(10–10′)H, 3 J = 8.0 Hz], 7.64 [d 2H, C(4–4′)H, 3 J = 16 Hz]. ^{13}C{1H}-NMR ($CDCl_3$): δ 14.0 [C(17-

17′)], 22.5 [C(15–15′)], 25.0 [C(13–13′)], 28.7 [C(14-14')], 31.5 [C(16–16′)], 34.1 [C(12–12′)], 55.9 [-OCH3], 101.7 [C(1)], 111.5 [C(6–6′)], 121.1 [C(10–10′)], 123.3 [(C(9–9′)], 124.2 [C(3–3′)], 133.9 30 [C(5–5′)], 140.0 [C(4–4′)], 141.5 [C(8–8′)], 151.5 [C(7–7′)], 171.6 [C(11–11′)], 183.1 [C(2–2′)=O]. ESIMS(-) CH_3OH (m/z [relative intensity, %]): 591 [100] $[L3a]^-$.

HL3b. To a solution of bisdemethoxycurcumin (300 mg, 0.97 mmol) in acetone (9 mL), was mixed triethylamine (TEA 0.41 mL, 2.92 mmol) and then, after 15 minutes, heptanoyl chloride (0.45 mL, 2.92 mmol) was added at 0 °C. under N_2 atmosphere. The reaction mixture was stirred at room temperature. Thin layer chromatography (7:3 hexane/ethylacetate) displayed the disappearance of bisdemethoxycurcumin and the formation of a faster running yellow spot after 4 hours. Evaporation of the solvent under reduced pressure provided a yellow solid which was then purified by crystallisation with dichloromethane/ ethanol giving HL3b (Figure 4) as yellow powder (yield 49%). The ligand is soluble in DMSO, acetone and chlorinated solvents, slightly soluble in alcohols and CH_3CN and insoluble in H_2O and n-hexane. Anal. Calcd for $C_{33}H_{40}O_6$: C, 74.41; H, 7.57. Found: C, 74.41; H, 7.59. m.p.: 171-172°C.IR (cm^{-1}): 2957 w, 2927 s, 2853 w ν(aliphatic C–H); 1756 s ν(–OC=O), 1627 m ν(C=O); 1586 m, 1556 m, 1506 m, 1465 m, 1414 m ν(C=C); 1379 m, 1344 w, 1320 w, 1283 w, 1213 s, 1165 s, 1133 s, 1105 s, 1036 m, 1014 m, 977 s, 955 s, 922 s, 865 m, 842 vs, 796 m, 779 m, 723 s. ^1H-NMR ($CDCl_3$, 293 K): δ 0.94 [t, 6H, C(17-17')*H*], 1.37 [m, 8H, C(16-16')*H* and C(15,15')*H*], 1.45 [m, 4H, C(14-14')*H*], 1.78 [quint, 4H, C(13–13′)*H*], 2.59 [t, 4H, C(12-12')*H*], 5.86 [s, 1H, C(1)*H*], 6.60 [d, 2H, C(3–3′)*H*, ^3J = 16 Hz], 7.15 [d, 4H, C(9–9′)*H* and C(7–7′)*H*, ^3J = 9 Hz], 7.59 [d, 4H, C(10–10′)*H* and C(6–6′)*H*, ^3J = 9 Hz], 7.67 [d, 2H, C(4–4′)*H*, ^3J = 16 Hz]. $^{13}C\{^1H\}$-NMR ($CDCl_3$, 293 K): δ 13.9 [*C*(17-17′)], 22.4 [*C*(16–16′)], 24.8 [*C*(13–13′)], 28.7 [*C*(14-14')], 31.4 [*C*(15–15′)], 34.4 [*C*(12–12′)], 101.8 [*C*(1)], 122.1 [*C*(9–9') and *C*(7–7′)], 124.1 [*C*(3–3′)], 129.2 [*C*(10–10′) and *C*(6–6′)], 132.6 [*C*(5–5′)], 139.5 [*C*(4–4′)], 152.1 [*C*(8–8′)], 172.1 [*C*(11–11′)], 182.7 [*C*(2–2′)=O]. ESI-MS(-) CH_3OH (m/z [relative intensity, %]): 531 [100] $[L3b]^-$.

HL4a. To a solution of curcumin (300 mg, 0.81 mmol) in acetone (9 mL) was mixed triethylamine (TEA 0.34 mL, 2.43 mmol) and then, after 15 minutes, cyclopenanecarbonyl chloride (0.29 mL, 2.43 mmol) was added at 0 °C under N_2 atmosphere. The reaction mixture was stirred at room temperature. Thin layer chromatography (7:3 hexane/ethylacetate) displayed the disappearance of curcumin and the formation of a faster running yellow spot after 3 hours. At this point the yellow precipitate was filtered with acetone and then purified by chromatographic column (7:3 hexane/ ethylacetate). At the end, the

solvent was evaporated under reduced pressure. The final ligand (Figure 1) has been obtained through crystallization in diethylether, precipitating as yellow powder (yield 66%). The ligand is soluble in DMSO, chlorinated solvents, slightly soluble in alcohols, acetone and CH_3CN and insoluble in H_2O and n-hexane. Anal. Calcd for $C_{33}H_{36}O_8$: C, 70.70; H, 6.47. Found: C, 70.02; H, 6.53. m.p.: 149–150°C. IR (cm^{-1}): 2969 wbr, 2871 wbr ν(aliphatic C–H); 1753 m, 1746m ν(–OC=O), 1622 w ν(C=O); 1598 w, 1586 w, 1556 w,1506 m, 1455 w, 1408 w ν(C=C); 1371 w, 1296 m, 1247 m, 1156 w, 1116 s, 1027 m, 982 m, 964 m, 890 w, 866 w, 844 m, 774 w, 732 w, 611 w. ^{1}H-NMR ($CDCl_3$, 293 K): δ 1.68 m, 181 m [8H, C(14-14')H and C(15-15')H], 2.05 [m, 8H, C(13-13')H and C(16-16')H], 3.06 [quint., 2H, C(12-12')H], 3.89 [s, 6H,-OCH3], 5.88 [s, 1H, C(1)H], 6.59 [d, 2H, C(3–3′)H, ^3J = 16 Hz], 7.07 [d, 2H, C(9-9')H, ^3J = 8 Hz], 7.14 [s, 2H, C(6-6')H], 7.18 [d, 2H, C(10-10')H, 3 J = 8 Hz], 7.65 [d, 2H, C(4–4′)H, ^3J = 16 Hz]. ^{13}C{1H}-NMR ($CDCl_3$): δ 25.9 [C(14-14') and C(15-15')], 30.1 [C(13-13') and C(16-16')], 43.7 [C(12– 12′)], 56.0 [-OCH3],101.6 [C(1)], 111.6 [C(6–6′)], 121.1 [C(10–10′)], 123.3 [C(9–9′)], 124.2 [C(3–3′)], 133.8 [C(5–5′)], 140.0 [C(4–4′)], 141.8 [C(8–8′)], 151.6 [C(7–7′)], 174.6 [C(11–11′)], 183.1 [C(2–2′)=O]. ESI-MS(-) CH_3OH (m/z [relative intensity, %]): 559 [100] $[L4a]^-$.

HL4b. To a solution of bisdemethoxycurcumin (300 mg, 0.97 mmol) in acetone (9 mL), was mixed triethylamine (TEA 0.41 mL, 2.92 mmol) and then, after 15 minutes, cyclopenanecarbonyl chloride (0.35 mL, 2.92 mmol) was added at 0 °C under N_2 atmosphere. The reaction mixture was stirred at room temperature. Thin layer chromatography (7:3 hexane/ethylacetate) displayed the disappearance of bisdemethoxycurcumin and the formation of a faster running yellow spot after 4 hours. At this point the yellow precipitate was filtered with acetone and then purified by crystallisation with dichloromethane/ ethanol giving the final ligand as yellow powder (yield 76%). The ligand is soluble in DMSO and chlorinated solvents, slightly soluble in alcohols, acetone and CH_3CN and insoluble in H_2O and n-hexane. Anal. Calcd for $C_{31}H_{32}O_6$: C, 74.38; H, 6.44. Found: C, 74.44; H, 6.41. m.p.: 218-220°C. IR (cm^{-1}): 2950 m, 2870 m ν(aliphatic C–H); 1743 s ν(–OC=O), 1632 m ν(C=O); 1567 m, 1505 m, 1447 m ν(C=C);1415 m, 1363 m, 1305 m, 1209 s, 1164 s, 1127 s, 1013 m, 967 s, 882 s, 860 s, 726 m, 696 m, 608 m. $_1$H-NMR ($CDCl_3$, 293 K): δ 1.69 m, 1.81 m [8H, C(14-14')H and C(15-15')H], 1.99 m, 2.05 [8H, C(13-13')H and C(16-16')H], 3.02 [quint, 2H, C(12-12')H], 5.87 [s, 1H, C(1)H], 6.60 [d, 2H, C(3–3′)H, ^3J = 16 Hz], 7.15 [d, 4H, C(9–9′)H and C(7–7′)H, ^3J = 9 Hz], 7.59 [d, 4H, C(10–10′)H and C(6–6′)H, ^3J = 9 Hz], 7.67 [d, 2H, C(4–4′)H, ^3J = 16 Hz], ^{13}C{^{1}H}-NMR ($CDCl_3$): δ 25.9 [C(14-14') and C(15-15')], 30.1 [C(13-13') and C(16-16')], 43.9 [C(12–12′)], 101.9 [C(1)], 122.2 [C(9–9') and C(7–7′)], 124.1 [C(3–3′)], 129.2 [C(10–10′) and C(6–6′)], 132.5 [C(5–5′)], 139.6 [C(4–4′)], 152.3 [C(8–8′)],

175.0 [C(11–11′)], 183.2 [C(2–2′)=O]. ESI-MS(-) CH_3OH (m/z [relative intensity, %]): 499 [100] $[L4b]^-$.

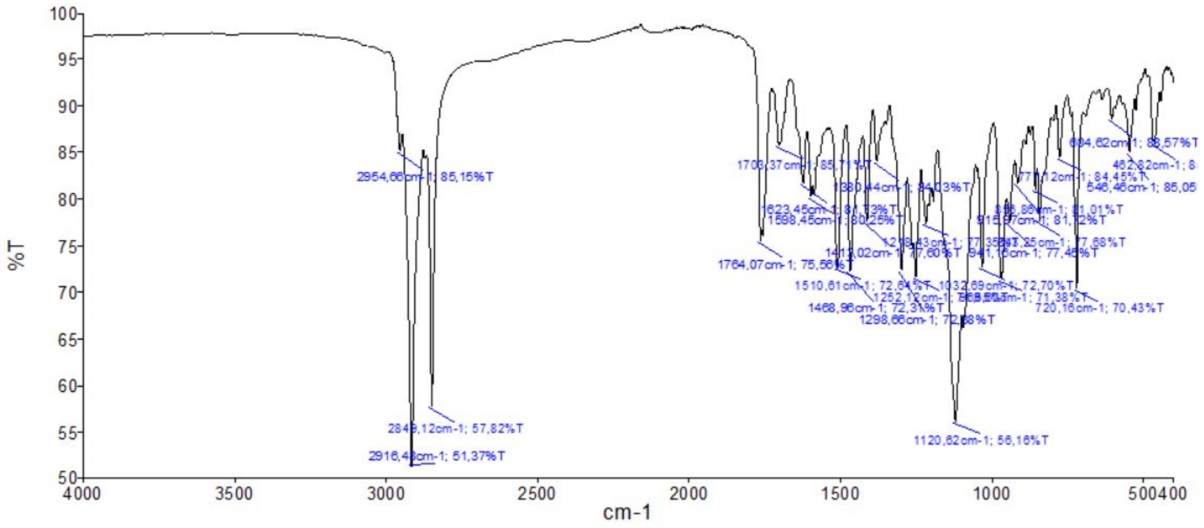

Figure S1: FT- IR Spectrum of HL2a.

Table S1: XYZ coordinates (in Å) of the optimized structures of the studied species (proligand and ligands) of the all studied curcumins computed at B3LYP/6-31++G(d,p) level of theory applying dispersion correction GD3BJ in dichlormethane.

**HL1a_e**				**HL1a_k**			
O	8.842017000000	0.880777000000	-0.000053000000	O	-7.206609000000	0.855289000000	-2.043679000000
O	6.765901000000	2.514023000000	0.000045000000	O	-6.509480000000	1.759831000000	0.345138000000
O	1.234756000000	-2.815064000000	-0.000065000000	O	0.382182000000	-3.206939000000	0.057057000000
O	-1.264794000000	-2.798960000000	-0.000074000000	O	-0.424775000000	0.532142000000	2.366100000000
O	-6.770157000000	2.511975000000	0.000103000000	O	6.134602000000	2.295439000000	-0.600890000000
O	-8.838685000000	0.868774000000	-0.000001000000	O	7.546746000000	0.246138000000	-1.479321000000
C	5.691253000000	3.457813000000	0.000098000000	C	-6.185507000000	2.292547000000	1.633051000000
C	5.184053000000	0.638475000000	0.000016000000	C	-4.498326000000	0.348473000000	0.368580000000
C	6.458444000000	1.181651000000	0.000007000000	C	-5.663433000000	0.837786000000	-0.205393000000
C	7.594681000000	0.337715000000	-0.000045000000	C	-6.066351000000	0.378970000000	-1.477365000000
C	7.432384000000	-1.042880000000	-0.000088000000	C	-5.294506000000	-0.564039000000	-2.155230000000
C	6.147775000000	-1.587902000000	-0.000079000000	C	-4.126243000000	-1.053272000000	-1.578868000000
C	5.007948000000	-0.765949000000	-0.000028000000	C	-3.708466000000	-0.607515000000	-0.311452000000
C	3.694495000000	-1.390440000000	-0.000022000000	C	-2.498593000000	-1.078732000000	0.344030000000
C	2.486842000000	-0.779721000000	0.000025000000	C	-1.593346000000	-1.962220000000	-0.145089000000
C	1.234574000000	-1.539761000000	0.000028000000	C	-0.389149000000	-2.383967000000	0.563303000000
C	0.001730000000	-0.806205000000	0.000033000000	C	-0.068971000000	-1.801324000000	1.945991000000
C	-1.219084000000	-1.464166000000	-0.000012000000	C	0.343163000000	-0.324238000000	1.911570000000
C	-2.478266000000	-0.752586000000	0.000007000000	C	1.629991000000	0.060306000000	1.342020000000
C	-3.680393000000	-1.378516000000	-0.000036000000	C	2.537374000000	-0.812849000000	0.836952000000
C	-4.996046000000	-0.759225000000	-0.000025000000	C	3.826535000000	-0.495270000000	0.246608000000
C	-6.132184000000	-1.586618000000	-0.000079000000	C	4.620296000000	-1.549491000000	-0.237640000000
C	-7.419295000000	-1.047830000000	-0.000071000000	C	5.865780000000	-1.307471000000	-0.816500000000
C	-7.588634000000	0.331918000000	-0.000009000000	C	6.334962000000	-0.002368000000	-0.917880000000
C	-6.456465000000	1.181190000000	0.000046000000	C	5.548691000000	1.072253000000	-0.435432000000
C	-5.179317000000	0.644372000000	0.000039000000	C	4.312076000000	0.829879000000	0.138406000000
C	-5.700255000000	3.461190000000	0.000166000000	C	5.426291000000	3.453967000000	-0.149608000000
H	8.752937000000	1.848509000000	-0.000019000000	H	-7.605401000000	1.498196000000	-1.433505000000

H	6.159450000000	4.441162000000	0.000120000000	H	-6.980895000000	2.997384000000	1.871135000000
H	5.073616000000	3.340306000000	0.896647000000	H	-5.223108000000	2.813782000000	1.604839000000
H	5.073579000000	3.340365000000	-0.896433000000	H	-6.157230000000	1.496845000000	2.384674000000
H	4.320600000000	1.290718000000	0.000056000000	H	-4.179216000000	0.696082000000	1.343974000000
H	8.312394000000	-1.676898000000	-0.000129000000	H	-5.622806000000	-0.904103000000	-3.131510000000
H	6.025555000000	-2.666375000000	-0.000113000000	H	-3.541081000000	-1.786691000000	-2.121321000000
H	3.690094000000	-2.478716000000	-0.000059000000	H	-2.319331000000	-0.647419000000	1.325277000000
H	2.395040000000	0.302374000000	0.000063000000	H	-1.714168000000	-2.417876000000	-1.123685000000
H	-0.287920000000	-3.093417000000	-0.000087000000	H	-0.940960000000	-1.872859000000	2.598797000000
H	0.017400000000	0.276402000000	0.000077000000	H	1.813572000000	1.130979000000	1.348937000000
H	-2.400531000000	0.329943000000	0.000057000000	H	2.297920000000	-1.872703000000	0.848269000000
H	-3.676550000000	-2.465880000000	-0.000085000000	H	3.716824000000	1.655469000000	0.505206000000
H	-4.320144000000	1.302178000000	0.000081000000	H	5.249936000000	3.402562000000	0.929820000000
H	-5.082032000000	3.346944000000	-0.896403000000	H	4.473451000000	3.554748000000	-0.679509000000
H	-5.082061000000	3.346857000000	0.896743000000	H	6.068870000000	4.302692000000	-0.379328000000
H	-6.173463000000	4.442128000000	0.000206000000	H	4.255338000000	-2.568754000000	-0.161361000000
H	-6.005197000000	-2.664544000000	-0.000128000000	H	6.479522000000	-2.119024000000	-1.191804000000
H	-8.296029000000	-1.686360000000	-0.000113000000	H	7.700281000000	1.205858000000	-1.462867000000
H	-8.754488000000	1.836936000000	0.000045000000	H	0.733559000000	-2.408907000000	2.368399000000
**HL1b_e**				**HL1b_k**			
O	-8.874747000000	1.446652000000	0.001489000000	O	7.439758000000	-1.968736000000	0.080570000000
O	-1.234080000000	-2.195727000000	-0.000007000000	O	-0.375003000000	1.523135000000	-2.172854000000
O	1.266064000000	-2.179975000000	-0.000198000000	O	0.373833000000	1.452942000000	2.220621000000
O	8.871805000000	1.437575000000	-0.001339000000	O	-7.456783000000	-1.908380000000	-0.277443000000
C	-5.200225000000	1.252014000000	0.000861000000	C	4.587976000000	0.157557000000	1.007684000000
C	-6.475326000000	1.802260000000	0.001113000000	C	5.804234000000	-0.508953000000	1.116918000000
C	-7.599162000000	0.961565000000	0.001232000000	C	6.258830000000	-1.289439000000	0.047299000000
C	-7.435618000000	-0.428152000000	0.001084000000	C	5.490873000000	-1.398329000000	-1.123214000000
C	-6.153690000000	-0.965448000000	0.000829000000	C	4.280626000000	-0.729725000000	-1.218663000000
C	-5.006820000000	-0.144879000000	0.000718000000	C	3.799008000000	0.065563000000	-0.155619000000
C	-3.693000000000	-0.770849000000	0.000472000000	C	2.539270000000	0.790201000000	-0.202235000000
C	-2.486023000000	-0.160038000000	0.000408000000	C	1.642075000000	0.805810000000	-1.218939000000
C	-1.233534000000	-0.920839000000	0.000148000000	C	0.386364000000	1.549826000000	-1.199371000000
C	-0.000611000000	-0.187218000000	0.000033000000	C	0.000729000000	2.370951000000	0.037738000000
C	1.219843000000	-0.845304000000	-0.000181000000	C	-0.387106000000	1.512028000000	1.248087000000
C	2.479188000000	-0.133085000000	-0.000371000000	C	-1.644106000000	0.769968000000	1.243459000000
C	3.680782000000	-0.758824000000	-0.000560000000	C	-2.540021000000	0.787778000000	0.225654000000
C	4.996517000000	-0.137378000000	-0.000765000000	C	-3.800449000000	0.066341000000	0.152664000000
C	6.140159000000	-0.962782000000	-0.000846000000	C	-4.590378000000	0.200581000000	-1.008273000000
C	7.424287000000	-0.431039000000	-0.001033000000	C	-5.804623000000	-0.460403000000	-1.142760000000
C	7.594123000000	0.957943000000	-0.001149000000	C	-6.260016000000	-1.280156000000	-0.103388000000
C	6.473850000000	1.803183000000	-0.001076000000	C	-5.493296000000	-1.431890000000	1.063190000000
C	5.196257000000	1.258713000000	-0.000890000000	C	-4.281326000000	-0.766201000000	1.184409000000
H	-8.867176000000	2.414608000000	0.001591000000	H	7.889581000000	-1.825644000000	0.925808000000
H	-4.345609000000	1.919972000000	0.000772000000	H	4.237635000000	0.761425000000	1.839559000000
H	-8.311969000000	-1.067183000000	0.001174000000	H	5.859538000000	-2.007397000000	-1.941468000000
H	-6.030967000000	-2.044610000000	0.000718000000	H	3.703041000000	-0.825395000000	-2.131535000000
H	-3.687416000000	-1.859305000000	0.000328000000	H	2.314196000000	1.361322000000	0.694611000000
H	-2.394651000000	0.922033000000	0.000549000000	H	1.809817000000	0.245731000000	-2.134429000000
H	0.289789000000	-2.475179000000	0.000022000000	H	0.835076000000	3.001670000000	0.349930000000
H	-0.016226000000	0.895378000000	0.000087000000	H	-1.813412000000	0.181394000000	2.140663000000
H	2.401814000000	0.949427000000	-0.000348000000	H	-2.313050000000	1.387436000000	-0.651858000000
H	3.676236000000	-1.846350000000	-0.000555000000	H	-3.704862000000	-0.896535000000	2.093663000000
H	4.345590000000	1.931667000000	-0.000848000000	H	-4.238532000000	0.834915000000	-1.816547000000
H	6.013246000000	-2.041465000000	-0.000756000000	H	-6.406345000000	-0.353179000000	-2.038636000000
H	8.297672000000	-1.074111000000	-0.001093000000	H	-7.673456000000	-2.445668000000	0.498123000000
H	8.868363000000	2.405572000000	-0.001419000000	H	-0.832058000000	3.013255000000	-0.254408000000
H	-6.605290000000	2.880976000000	0.001223000000	H	-5.849957000000	-2.068938000000	1.867779000000
H	6.608266000000	2.881305000000	-0.001174000000	H	6.396382000000	-0.425139000000	2.023764000000
**HL3a_e**				**HL3a_k**			
O	8.856576000000	-0.738062000000	0.634835000000	O	-8.090982000000	-0.479395000000	-1.003649000000
O	6.736668000000	0.726533000000	1.375439000000	O	-7.244027000000	-0.686741000000	1.543842000000
O	1.232650000000	-4.099572000000	-0.875741000000	O	-0.328262000000	-4.766936000000	-1.187267000000
O	-1.268744000000	-4.081207000000	-0.873060000000	O	-1.096734000000	-2.524536000000	2.574047000000
O	-6.730431000000	0.742898000000	1.381579000000	O	5.413036000000	0.435655000000	0.765078000000
O	-8.847597000000	-0.727919000000	0.644807000000	O	6.893682000000	-0.997262000000	-0.941183000000
C	5.639108000000	1.548099000000	1.782058000000	C	-6.839173000000	-0.789361000000	2.913641000000
C	5.184983000000	-0.972011000000	0.557530000000	C	-5.244352000000	-1.904512000000	0.839645000000
C	6.460880000000	-0.460588000000	0.777848000000	C	-6.442322000000	-1.232903000000	0.594284000000
C	7.578080000000	-1.215267000000	0.363042000000	C	-6.866602000000	-1.078255000000	-0.738654000000
C	7.423336000000	-2.451030000000	-0.241320000000	C	-6.123856000000	-1.600348000000	-1.791507000000
C	6.141427000000	-2.961869000000	-0.453694000000	C	-4.930032000000	-2.270809000000	-1.541286000000
C	5.010063000000	-2.228747000000	-0.061748000000	C	-4.475834000000	-2.427824000000	-0.220325000000
C	3.690806000000	-2.800988000000	-0.311403000000	C	-3.231964000000	-3.113082000000	0.121667000000
C	2.489192000000	-2.251206000000	-0.032389000000	C	-2.334213000000	-3.640122000000	-0.741893000000
C	1.232317000000	-2.943660000000	-0.343836000000	C	-1.092825000000	-4.303666000000	-0.337315000000
C	0.000723000000	-2.275763000000	-0.033611000000	C	-0.734833000000	-4.418497000000	1.149293000000
C	-1.218623000000	-2.871319000000	-0.314130000000	C	-0.330372000000	-3.082115000000	1.783184000000

C	-2.479510000000	-2.219513000000	-0.021983000000
C	-3.677852000000	-2.781604000000	-0.300912000000
C	-4.997274000000	-2.210979000000	-0.050696000000
C	-6.127541000000	-2.947530000000	-0.440282000000
C	-7.410357000000	-2.439274000000	-0.228275000000
C	-7.568045000000	-1.202631000000	0.373572000000
C	-6.452315000000	-0.444683000000	0.786162000000
C	-5.175203000000	-0.953386000000	0.566278000000
C	-5.634764000000	1.568053000000	1.786076000000
H	6.086323000000	2.441697000000	2.215547000000
H	5.026931000000	1.039577000000	2.534619000000
H	5.019732000000	1.824319000000	0.922243000000
H	4.319039000000	-0.403373000000	0.868289000000
H	8.306991000000	-3.002771000000	-0.542724000000
H	6.017564000000	-3.929421000000	-0.928462000000
H	3.683711000000	-3.783219000000	-0.779300000000
H	2.400486000000	-1.271958000000	0.428275000000
H	-0.295007000000	-4.354631000000	-0.996988000000
H	0.017232000000	-1.292567000000	0.419074000000
H	-2.401231000000	-1.238531000000	0.435402000000
H	-3.674855000000	-3.764600000000	-0.765047000000
H	-4.311460000000	-0.381019000000	0.876199000000
H	-5.016506000000	1.844217000000	0.925436000000
H	-5.020984000000	1.062589000000	2.539388000000
H	-6.084069000000	2.461341000000	2.218022000000
H	-6.002118000000	-3.915795000000	-0.913191000000
H	-8.292701000000	-2.993936000000	-0.528158000000
C	9.327884000000	0.301828000000	-0.127936000000
O	8.730055000000	0.732051000000	-1.089004000000
C	10.649899000000	0.785736000000	0.405437000000
C	11.320870000000	1.837151000000	-0.476684000000
H	11.296146000000	-0.089162000000	0.545164000000
H	10.462751000000	1.179787000000	1.413074000000
C	12.654097000000	2.314499000000	0.106046000000
H	11.483371000000	1.421025000000	-1.478272000000
H	10.644840000000	2.690830000000	-0.602444000000
C	13.346060000000	3.366858000000	-0.765206000000
H	12.485436000000	2.728525000000	1.110239000000
H	13.324868000000	1.453559000000	0.236492000000
H	13.514336000000	2.952530000000	-1.769500000000
H	12.674676000000	4.227451000000	-0.896458000000
C	14.680596000000	3.851439000000	-0.189494000000
H	15.349990000000	2.990355000000	-0.057984000000
H	14.510976000000	4.264888000000	0.814074000000
C	15.365328000000	4.903020000000	-1.067613000000
H	16.315487000000	5.231789000000	-0.633157000000
H	15.574199000000	4.504317000000	-2.067402000000
H	14.728920000000	5.787446000000	-1.189891000000
C	-9.322644000000	0.307498000000	-0.121714000000
O	-8.726230000000	0.736655000000	-1.084153000000
C	-10.646651000000	0.788193000000	0.409617000000
C	-11.322126000000	1.832823000000	-0.477126000000
H	-10.461168000000	1.187467000000	1.415506000000
H	-11.289180000000	-0.088806000000	0.553206000000
C	-12.658016000000	2.306035000000	0.102881000000
H	-10.650155000000	2.689238000000	-0.605986000000
H	-11.482049000000	1.411839000000	-1.477095000000
H	-13.324511000000	1.442299000000	0.236733000000
H	-12.491980000000	2.725271000000	1.105347000000
C	-13.354843000000	3.351042000000	-0.773326000000
C	-14.692267000000	3.831137000000	-0.200561000000
H	-13.520317000000	2.931506000000	-1.775924000000
H	-12.687852000000	4.214534000000	-0.907879000000
C	-15.381999000000	4.875168000000	-1.083756000000
H	-15.357187000000	2.967120000000	-0.065630000000
H	-14.525457000000	4.249926000000	0.801262000000
H	-16.334146000000	5.200840000000	-0.651327000000
H	-14.750199000000	5.762390000000	-1.209600000000
H	-15.588155000000	4.470918000000	-2.081880000000

C	0.955623000000	-2.472052000000	1.444361000000
C	1.858504000000	-3.013512000000	0.593848000000
C	3.148224000000	-2.449406000000	0.209156000000
C	3.940666000000	-3.167279000000	-0.700598000000
C	5.181155000000	-2.668603000000	-1.102421000000
C	5.629465000000	-1.460364000000	-0.597020000000
C	4.859067000000	-0.721627000000	0.326520000000
C	3.620511000000	-1.220491000000	0.718565000000
C	4.656680000000	1.249710000000	1.665574000000
H	-7.615897000000	-0.284296000000	3.485912000000
H	-5.877154000000	-0.291550000000	3.073467000000
H	-6.774060000000	-1.837347000000	3.223184000000
H	-4.889219000000	-2.032527000000	1.854723000000
H	-6.493896000000	-1.472014000000	-2.802923000000
H	-4.362331000000	-2.669662000000	-2.373215000000
H	-3.027664000000	-3.175385000000	1.186837000000
H	-2.480307000000	-3.594941000000	-1.817106000000
H	-1.587051000000	-4.796214000000	1.717062000000
H	1.134250000000	-1.521164000000	1.937881000000
H	1.624675000000	-3.962823000000	0.120078000000
H	3.017775000000	-0.663989000000	1.423052000000
H	4.457476000000	0.717197000000	2.601667000000
H	3.713358000000	1.564169000000	1.206904000000
H	5.278607000000	2.121524000000	1.864233000000
H	3.583861000000	-4.111939000000	-1.097014000000
H	5.804087000000	-3.207396000000	-1.807746000000
H	0.082231000000	-5.138021000000	1.225294000000
C	6.975100000000	0.103047000000	-1.759468000000
O	6.008292000000	0.578647000000	-2.313966000000
C	8.385351000000	0.617278000000	-1.823350000000
C	8.706910000000	1.495447000000	-0.595507000000
H	8.487329000000	1.200193000000	-2.741944000000
H	9.078227000000	-0.228948000000	-1.862017000000
C	10.130254000000	2.056522000000	-0.647356000000
H	7.986180000000	2.320682000000	-0.545908000000
H	8.573946000000	0.902076000000	0.316103000000
H	10.846229000000	1.224894000000	-0.704007000000
H	10.258135000000	2.639998000000	-1.569550000000
C	10.466852000000	2.935722000000	0.560836000000
C	11.889338000000	3.503706000000	0.520848000000
H	10.336208000000	2.351208000000	1.482675000000
H	9.747621000000	3.765186000000	0.617503000000
C	12.217161000000	4.381950000000	1.732002000000
H	12.606915000000	2.673970000000	0.463505000000
H	12.018500000000	4.086403000000	-0.401343000000
H	13.238269000000	4.774389000000	1.678204000000
H	11.532732000000	5.236224000000	1.794541000000
H	12.125140000000	3.814188000000	2.665456000000
C	-8.261597000000	0.883782000000	-0.906796000000
O	-9.386106000000	1.309313000000	-1.046280000000
C	-7.028962000000	1.721473000000	-0.669089000000
C	-7.297862000000	3.224123000000	-0.738488000000
H	-6.264328000000	1.424321000000	-1.397116000000
H	-6.627550000000	1.450883000000	0.314476000000
C	-6.025230000000	4.037554000000	-0.476153000000
H	-7.701336000000	3.478959000000	-1.725885000000
H	-8.073426000000	3.480404000000	-0.010485000000
H	-5.646883000000	3.805031000000	0.529632000000
H	-5.246116000000	3.715319000000	-1.179939000000
C	-6.216144000000	5.554999000000	-0.606156000000
C	-7.162655000000	6.168919000000	0.432832000000
H	-6.585969000000	5.788678000000	-1.614916000000
H	-5.235357000000	6.041725000000	-0.518950000000
C	-7.248801000000	7.694395000000	0.324312000000
H	-8.167016000000	5.742713000000	0.321263000000
H	-6.819071000000	5.890111000000	1.438779000000
H	-7.929975000000	8.112157000000	1.073380000000
H	-6.264886000000	8.155597000000	0.470792000000
H	-7.612839000000	7.997262000000	-0.664627000000

HL3b_e

O	8.812890000000	0.010586000000	-0.944754000000
O	1.231839000000	3.363147000000	0.823579000000
O	-1.271504000000	3.345641000000	0.819126000000
O	-8.807858000000	-0.000327000000	-0.950205000000
C	5.175936000000	0.303258000000	-0.770512000000
C	6.451231000000	-0.191751000000	-1.017509000000
C	7.570965000000	0.543907000000	-0.599325000000
C	7.426213000000	1.764016000000	0.048143000000
C	6.146112000000	2.253874000000	0.297682000000
C	5.005706000000	1.531865000000	-0.105885000000
C	3.689629000000	2.095348000000	0.181649000000
C	2.486544000000	1.560882000000	-0.118037000000
C	1.230590000000	2.236816000000	0.232204000000
C	-0.001150000000	1.585702000000	-0.111400000000
C	-1.220307000000	2.166277000000	0.198400000000

HL3b_k

O	-8.246461000000	0.319455000000	0.202487000000
O	-0.542388000000	4.519081000000	1.553446000000
O	-1.121455000000	3.005148000000	-2.589918000000
O	6.648187000000	0.722790000000	1.019202000000
C	-5.353198000000	2.092392000000	-1.170919000000
C	-6.552211000000	1.385960000000	-1.092368000000
C	-7.022904000000	0.986107000000	0.163847000000
C	-6.337063000000	1.323185000000	1.329268000000
C	-5.141440000000	2.025839000000	1.247432000000
C	-4.630669000000	2.413068000000	-0.008873000000
C	-3.375872000000	3.145130000000	-0.168202000000
C	-2.519689000000	3.501516000000	0.815795000000
C	-1.262437000000	4.221462000000	0.597420000000
C	-0.833176000000	4.600726000000	-0.824370000000
C	-0.398436000000	3.398322000000	-1.670801000000

C	-2.480281000000	1.530033000000	-0.130186000000	C	0.865859000000	2.719138000000	-1.382182000000
C	-3.680008000000	2.076866000000	0.170399000000	C	1.723358000000	3.082944000000	-0.401044000000
C	-4.996332000000	1.514780000000	-0.116558000000	C	2.986468000000	2.435975000000	-0.058873000000
C	-6.135466000000	2.241130000000	0.283727000000	C	3.718428000000	2.939698000000	1.030824000000
C	-7.416551000000	1.753508000000	0.035777000000	C	4.926701000000	2.351562000000	1.402610000000
C	-7.564566000000	0.531246000000	-0.606935000000	C	5.410620000000	1.263880000000	0.690841000000
C	-6.446465000000	-0.208520000000	-1.021992000000	C	4.703263000000	0.748263000000	-0.409609000000
C	-5.169871000000	0.284054000000	-0.776681000000	C	3.496239000000	1.334274000000	-0.773997000000
H	4.326608000000	-0.281869000000	-1.103430000000	H	-4.985601000000	2.390017000000	-2.147490000000
H	8.305886000000	2.316608000000	0.356201000000	H	-6.746488000000	1.028000000000	2.289403000000
H	6.025103000000	3.203934000000	0.807327000000	H	-4.614069000000	2.277657000000	2.159421000000
H	3.687671000000	3.053673000000	0.696719000000	H	-3.127206000000	3.398427000000	-1.194948000000
H	2.396093000000	0.606870000000	-0.628537000000	H	-2.713784000000	3.262011000000	1.857118000000
H	-0.298512000000	3.612694000000	0.958033000000	H	-1.657367000000	5.081670000000	-1.354110000000
H	0.015322000000	0.627433000000	-0.614644000000	H	1.065934000000	1.870668000000	-2.030029000000
H	-2.400373000000	0.573981000000	-0.637307000000	H	1.469141000000	3.931943000000	0.227370000000
H	-3.681777000000	3.036445000000	0.681128000000	H	2.956446000000	0.930912000000	-1.624526000000
H	-4.322994000000	-0.305576000000	-1.107847000000	H	3.336125000000	3.787963000000	1.588548000000
H	-6.012674000000	3.192861000000	0.789828000000	H	5.500289000000	2.725667000000	2.243357000000
H	-8.294734000000	2.309698000000	0.341604000000	H	-0.013470000000	5.315095000000	-0.729942000000
C	9.711421000000	-0.318742000000	0.052875000000	C	6.671847000000	-0.511434000000	1.622434000000
O	9.464030000000	-0.176833000000	1.226837000000	O	5.665007000000	-1.082270000000	1.980970000000
C	10.979369000000	-0.857801000000	-0.552459000000	C	8.080746000000	-1.022754000000	1.732404000000
C	12.035566000000	-1.240775000000	0.482815000000	C	8.591115000000	-1.559857000000	0.379117000000
H	11.360178000000	-0.098404000000	-1.246999000000	H	8.087922000000	-1.816232000000	2.483398000000
H	10.706507000000	-1.718317000000	-1.176824000000	H	8.730486000000	-0.209830000000	2.072520000000
C	13.309749000000	-1.788024000000	-0.166777000000	C	10.017819000000	-2.105883000000	0.480088000000
H	12.279144000000	-0.363668000000	1.093796000000	H	7.916975000000	-2.350866000000	0.028596000000
H	11.618573000000	-1.988795000000	1.167202000000	H	8.553081000000	-0.756170000000	-0.364744000000
C	14.381875000000	-2.175616000000	0.855814000000	H	10.685306000000	-1.310988000000	0.840896000000
H	13.059022000000	-2.664991000000	-0.780079000000	H	10.049696000000	-2.904303000000	1.234250000000
H	13.720107000000	-1.036642000000	-0.856032000000	C	10.544417000000	-2.645128000000	-0.853261000000
H	14.632181000000	-1.298184000000	1.468969000000	C	11.972692000000	-3.192776000000	-0.764682000000
H	13.970225000000	-2.925584000000	1.546167000000	H	10.509333000000	-1.845871000000	-1.607197000000
C	15.661092000000	-2.726260000000	0.217328000000	H	9.874360000000	-3.438395000000	-1.214087000000
H	16.070869000000	-1.976292000000	-0.472925000000	C	12.491644000000	-3.729923000000	-2.101776000000
H	15.409472000000	-3.602811000000	-0.395174000000	H	12.640836000000	-2.399239000000	-0.403298000000
C	16.726953000000	-3.110427000000	1.247896000000	H	12.006374000000	-3.990475000000	-0.010220000000
H	17.629582000000	-3.500313000000	0.765361000000	H	13.513197000000	-4.114161000000	-2.010268000000
H	17.018258000000	-2.244024000000	1.853387000000	H	11.858487000000	-4.545880000000	-2.469821000000
H	16.352594000000	-3.881643000000	1.931424000000	H	12.496626000000	-2.943548000000	-2.865805000000
C	-9.706550000000	-0.324268000000	0.049053000000	C	-8.314273000000	-1.048721000000	0.421518000000
O	-9.458231000000	-0.178651000000	1.222378000000	O	-9.417720000000	-1.531622000000	0.510073000000
C	-10.976001000000	-0.862749000000	-0.553640000000	C	-7.008536000000	-1.799576000000	0.505502000000
C	-12.032964000000	-1.238429000000	0.483532000000	C	-7.189652000000	-3.317225000000	0.520099000000
H	-10.705460000000	-1.726755000000	-1.174199000000	H	-6.480976000000	-1.465411000000	1.407348000000
H	-11.355010000000	-0.105499000000	-1.251495000000	H	-6.374609000000	-1.487417000000	-0.333389000000
C	-13.309117000000	-1.784218000000	-0.163411000000	C	-5.842015000000	-4.044049000000	0.595277000000
H	-11.618013000000	-1.985062000000	1.170673000000	H	-7.811931000000	-3.601946000000	1.376290000000
H	-12.273587000000	-0.358189000000	1.091169000000	H	-7.737982000000	-3.619552000000	-0.377725000000
H	-13.717419000000	-1.034211000000	-0.855381000000	H	-5.239238000000	-3.784274000000	-0.286504000000
H	-13.061404000000	-2.664319000000	-0.773437000000	H	-5.287225000000	-3.673597000000	1.467661000000
C	-14.382018000000	-2.164486000000	0.861117000000	C	-5.962136000000	-5.571087000000	0.693854000000
C	-15.663370000000	-2.713225000000	0.225277000000	C	-6.582308000000	-6.242144000000	-0.537970000000
H	-14.629124000000	-1.283972000000	1.471144000000	H	-6.553032000000	-5.831722000000	1.583685000000
H	-13.972518000000	-2.913258000000	1.554041000000	H	-4.960840000000	-5.992018000000	0.856619000000
C	-16.729991000000	-3.090055000000	1.257764000000	C	-6.589942000000	-7.770459000000	-0.435838000000
H	-16.070997000000	-1.964435000000	-0.467523000000	H	-7.609853000000	-5.885847000000	-0.680153000000
H	-15.414968000000	-3.592847000000	-0.384126000000	H	-6.022552000000	-5.935591000000	-1.432581000000
H	-17.634153000000	-3.478697000000	0.777102000000	H	-7.038201000000	-8.229653000000	-1.323413000000
H	-16.357867000000	-3.859998000000	1.943941000000	H	-5.571363000000	-8.162952000000	-0.332796000000
H	-17.018113000000	-2.220468000000	1.860208000000	H	-7.163019000000	-8.102324000000	0.438067000000
O	6.591597000000	-1.384406000000	-1.662655000000	O	5.259581000000	-0.307631000000	-1.065687000000
H	7.536406000000	-1.559681000000	-1.796814000000	H	4.677504000000	-0.603701000000	-1.779883000000
O	-6.589408000000	-1.403184000000	-1.662700000000	O	-7.225419000000	1.086720000000	-2.237001000000
H	-7.534604000000	-1.576781000000	-1.796366000000	H	-8.085518000000	0.698022000000	-2.011568000000
**HL4a_e**				**HL4a_k**			
O	8.836647000000	-0.106879000000	-0.641809000000	O	7.627181000000	0.432873000000	1.524362000000
O	6.703424000000	-1.679218000000	-1.054753000000	O	7.131415000000	-0.573103000000	-0.908956000000
O	1.236616000000	3.486698000000	0.388479000000	O	-0.240714000000	-3.628666000000	1.969703000000
O	-1.266160000000	3.482604000000	0.378381000000	O	1.077659000000	-2.642487000000	-2.164806000000
O	-6.733050000000	-1.629232000000	-1.092359000000	O	-5.494973000000	0.710977000000	-2.209203000000
O	-8.850927000000	-0.034265000000	-0.688315000000	O	-7.205157000000	-0.087996000000	-0.305323000000
C	5.598168000000	-2.556634000000	-1.285410000000	C	6.889613000000	-1.041300000000	-2.239316000000
C	5.167515000000	0.143928000000	-0.527892000000	C	5.000656000000	-1.510265000000	-0.156833000000
C	6.439290000000	-0.402467000000	-0.677766000000	C	6.181282000000	-0.793940000000	0.034177000000
C	7.563535000000	0.412550000000	-0.430629000000	C	6.429715000000	-0.224922000000	1.297711000000
C	7.419378000000	1.737140000000	-0.054905000000	C	5.533565000000	-0.391467000000	2.346914000000
C	6.141585000000	2.281035000000	0.088985000000	C	4.358892000000	-1.113236000000	2.153021000000
C	5.003430000000	1.492230000000	-0.142619000000	C	4.077474000000	-1.677627000000	0.896564000000
C	3.688535000000	2.103932000000	0.022446000000	C	2.863221000000	-2.438800000000	0.616063000000

C	2.482760000000	1.519931000000	-0.147680000000	C	1.844536000000	-2.682548000000	1.472202000000
C	1.230145000000	2.259803000000	0.051284000000	C	0.641383000000	-3.442257000000	1.127524000000
C	-0.004880000000	1.556527000000	-0.146855000000	C	0.470107000000	-4.005369000000	-0.288509000000
C	-1.221398000000	2.197235000000	0.025247000000	C	0.199358000000	-2.928434000000	-1.345959000000
C	-2.484597000000	1.513973000000	-0.167444000000	C	-1.092809000000	-2.242682000000	-1.376905000000
C	-3.681865000000	2.121350000000	-0.002231000000	C	-2.110534000000	-2.491372000000	-0.520144000000
C	-5.001865000000	1.523311000000	-0.173472000000	C	-3.412630000000	-1.833247000000	-0.490393000000
C	-6.132644000000	2.324539000000	0.053056000000	C	-4.323498000000	-2.213341000000	0.508046000000
C	-7.415585000000	1.794666000000	-0.096058000000	C	-5.580071000000	-1.610217000000	0.581912000000
C	-7.573020000000	0.471611000000	-0.471838000000	C	-5.928120000000	-0.635583000000	-0.337746000000
C	-6.456712000000	-0.355569000000	-0.714206000000	C	-5.036644000000	-0.239495000000	-1.357253000000
C	-5.179562000000	0.176758000000	-0.559304000000	C	-3.782237000000	-0.839669000000	-1.422345000000
C	-5.636914000000	-2.519727000000	-1.316280000000	C	-4.617508000000	1.173720000000	-3.239841000000
H	6.036897000000	-3.515865000000	-1.557166000000	H	7.753513000000	-0.724576000000	-2.821733000000
H	4.972508000000	-2.191039000000	-2.106759000000	H	5.979857000000	-0.590928000000	-2.649887000000
H	4.994099000000	-2.669899000000	-0.378977000000	H	6.808934000000	-2.132842000000	-2.260238000000
H	4.296327000000	-0.469947000000	-0.711875000000	H	4.780610000000	-1.951343000000	-1.121259000000
H	8.308534000000	2.332100000000	0.122932000000	H	5.767535000000	0.053944000000	3.307645000000
H	6.026199000000	3.318612000000	0.384127000000	H	3.668647000000	-1.231401000000	2.979549000000
H	3.689143000000	3.151356000000	0.316759000000	H	2.793892000000	-2.823278000000	-0.397704000000
H	2.387377000000	0.478066000000	-0.438193000000	H	1.855688000000	-2.313441000000	2.493500000000
H	-0.291405000000	3.767144000000	0.460747000000	H	1.372806000000	-4.535395000000	-0.597575000000
H	0.006866000000	0.512761000000	-0.433662000000	H	-1.173259000000	-1.491191000000	-2.156915000000
H	-2.409104000000	0.470579000000	-0.455788000000	H	-1.971960000000	-3.245769000000	0.249273000000
H	-3.677671000000	3.168362000000	0.290083000000	H	-3.087264000000	-0.545455000000	-2.196831000000
H	-4.315476000000	-0.447874000000	-0.740322000000	H	-4.346931000000	0.358244000000	-3.919047000000
H	-5.039140000000	-2.639184000000	-0.406467000000	H	-3.713078000000	1.617962000000	-2.811163000000
H	-5.002651000000	-2.162424000000	-2.134677000000	H	-5.178462000000	1.933061000000	-3.783001000000
H	-6.085436000000	-3.474010000000	-1.589433000000	H	-4.045749000000	-2.975256000000	1.228637000000
H	-6.007606000000	3.360913000000	0.348480000000	H	-6.294906000000	-1.887841000000	1.348683000000
H	-8.298654000000	2.399755000000	0.077935000000	H	-0.357153000000	-4.716349000000	-0.250627000000
O	8.760503000000	-1.224895000000	1.329805000000	O	-6.413068000000	1.823651000000	0.622436000000
O	-8.790172000000	-1.155118000000	1.281705000000	O	9.104323000000	1.962327000000	0.926169000000
C	9.347120000000	-0.963388000000	0.302277000000	C	-7.344620000000	1.192499000000	0.173738000000
C	-9.373393000000	-0.885363000000	0.253947000000	C	7.948234000000	1.604442000000	0.870309000000
C	10.716900000000	-1.432955000000	-0.110171000000	C	-8.788298000000	1.614643000000	0.111358000000
C	11.767916000000	-0.299182000000	0.145586000000	C	-9.596851000000	0.919747000000	1.260033000000
C	11.222704000000	-2.668206000000	0.683431000000	C	-9.004095000000	3.140413000000	0.302163000000
H	10.675955000000	-1.645969000000	-1.181685000000	H	-9.180146000000	1.280838000000	-0.853245000000
C	13.020837000000	-1.049328000000	0.621649000000	C	-10.595270000000	1.992910000000	1.719188000000
H	11.415508000000	0.363748000000	0.944629000000	H	-8.926219000000	0.668393000000	2.090181000000
H	11.928226000000	0.313237000000	-0.744774000000	H	-10.065433000000	-0.007492000000	0.922260000000
C	12.443481000000	-2.177881000000	1.487726000000	C	-9.779902000000	3.290454000000	1.626143000000
H	11.525465000000	-3.445988000000	-0.024598000000	H	-9.602898000000	3.521826000000	-0.530794000000
H	10.440110000000	-3.086122000000	1.320293000000	H	-8.056811000000	3.683837000000	0.305915000000
H	13.555336000000	-1.472265000000	-0.238602000000	H	-11.447060000000	2.034522000000	1.028315000000
H	13.717056000000	-0.399016000000	1.160559000000	H	-10.987815000000	1.795741000000	2.721716000000
H	13.161170000000	-2.978116000000	1.692276000000	H	-10.397239000000	4.193508000000	1.651737000000
H	12.118545000000	-1.769224000000	2.452559000000	H	-9.079108000000	3.343745000000	2.468552000000
C	-10.751699000000	-1.336864000000	-0.153567000000	C	6.844557000000	2.390190000000	0.205364000000
C	-11.152148000000	-2.700870000000	0.462856000000	C	6.721599000000	3.810603000000	0.823251000000
C	-11.826019000000	-0.306748000000	0.358966000000	C	7.109455000000	2.623816000000	-1.320324000000
H	-10.774340000000	-1.368947000000	-1.244802000000	H	5.893824000000	1.872074000000	0.332266000000
C	-12.603276000000	-2.517313000000	0.939557000000	C	6.211570000000	4.667694000000	-0.342863000000
H	-10.506050000000	-2.909925000000	1.320880000000	H	7.711316000000	4.161692000000	1.133527000000
H	-11.031307000000	-3.518578000000	-0.253080000000	H	6.066311000000	3.818085000000	1.699004000000
C	-12.625012000000	-1.064434000000	1.433403000000	C	7.038518000000	4.153464000000	-1.530367000000
H	-12.476114000000	-0.034939000000	-0.478006000000	H	6.334753000000	2.110776000000	-1.896277000000
H	-11.377517000000	0.617915000000	0.733469000000	H	8.071360000000	2.210234000000	-1.630775000000
H	-13.299651000000	-2.635410000000	0.099434000000	H	5.143784000000	4.477955000000	-0.511753000000
H	-12.886302000000	-3.241487000000	1.709691000000	H	6.335096000000	5.739956000000	-0.161817000000
H	-13.634551000000	-0.661423000000	1.560990000000	H	6.610579000000	4.418947000000	-2.501786000000
H	-12.113664000000	-0.995989000000	2.401410000000	H	8.045750000000	4.583660000000	-1.484689000000
**HL4b_e**				**HL4b_k**			
O	8.828215000000	-0.774184000000	-0.565166000000	O	-7.867715000000	-0.366513000000	0.467130000000
O	1.229627000000	2.890256000000	0.193869000000	O	0.063804000000	3.168347000000	2.322682000000
O	-1.271683000000	2.873027000000	0.185109000000	O	-1.046077000000	3.254269000000	-1.977209000000
O	-8.821057000000	-0.788859000000	-0.606572000000	O	7.091001000000	0.086777000000	-0.428594000000
C	5.188096000000	-0.482890000000	-0.446772000000	C	-5.116958000000	1.852158000000	-0.485318000000

C	6.467731000000	-1.017284000000	-0.547012000000
C	7.580900000000	-0.179894000000	-0.377133000000
C	7.425745000000	1.175631000000	-0.118025000000
C	6.142030000000	1.705960000000	-0.014025000000
C	5.007630000000	0.886147000000	-0.175882000000
C	3.687787000000	1.499355000000	-0.059062000000
C	2.487108000000	0.892844000000	-0.174929000000
C	1.229573000000	1.638986000000	-0.036962000000
C	-0.001654000000	0.914534000000	-0.172213000000
C	-1.221004000000	1.562285000000	-0.056019000000
C	-2.481911000000	0.860316000000	-0.190298000000
C	-3.679752000000	1.478789000000	-0.083107000000
C	-4.999131000000	0.866473000000	-0.204407000000
C	-6.133117000000	1.691053000000	-0.062616000000
C	-7.417107000000	1.162905000000	-0.171664000000
C	-7.574026000000	-0.195400000000	-0.415065000000
C	-6.461637000000	-1.037582000000	-0.564330000000
C	-5.181364000000	-0.505460000000	-0.459785000000
H	4.343465000000	-1.148199000000	-0.583365000000
H	8.301371000000	1.802147000000	0.005155000000
H	6.012952000000	2.763321000000	0.191747000000
H	3.680097000000	2.568030000000	0.144687000000
H	2.400077000000	-0.170717000000	-0.375312000000
H	-0.298126000000	3.168060000000	0.242809000000
H	0.015256000000	-0.150007000000	-0.368028000000
H	-2.404509000000	-0.204281000000	-0.385747000000
H	-3.676975000000	2.547783000000	0.113961000000
H	-4.338475000000	-1.176049000000	-0.580164000000
H	-6.003456000000	2.750577000000	0.131396000000
H	-8.291999000000	1.793256000000	-0.063859000000
O	9.477932000000	-0.341399000000	1.570347000000
O	-9.493944000000	-0.310918000000	1.511339000000
C	9.742382000000	-0.774745000000	0.472921000000
C	-9.749324000000	-0.761561000000	0.418563000000
C	11.059289000000	-1.322259000000	-0.003725000000
C	11.830356000000	-0.206159000000	-0.793762000000
C	12.004541000000	-1.759074000000	1.147635000000
H	10.844515000000	-2.155994000000	-0.677090000000
C	13.292960000000	-0.370165000000	-0.351658000000
H	11.468593000000	0.784149000000	-0.493423000000
H	11.679986000000	-0.298318000000	-1.871721000000
C	13.172302000000	-0.754343000000	1.129577000000
H	12.374525000000	-2.767395000000	0.937127000000
H	11.487067000000	-1.789102000000	2.108935000000
H	13.766109000000	-1.186073000000	-0.912574000000
H	13.881840000000	0.536705000000	-0.520590000000
H	14.093538000000	-1.173207000000	1.545444000000
H	12.914031000000	0.133236000000	1.720282000000
C	-11.068126000000	-1.300263000000	-0.066930000000
C	-11.967526000000	-1.831908000000	1.078258000000
C	-11.885339000000	-0.139917000000	-0.747968000000
H	-10.853239000000	-2.073670000000	-0.807517000000
C	-13.361227000000	-1.234098000000	0.812987000000
H	-11.584184000000	-1.465194000000	2.035062000000
H	-11.967482000000	-2.924595000000	1.116012000000
C	-13.044558000000	0.145602000000	0.220367000000
H	-12.270484000000	-0.502478000000	-1.705678000000
H	-11.272362000000	0.741304000000	-0.958789000000
H	-13.902648000000	-1.839477000000	0.074937000000
H	-13.974810000000	-1.187154000000	1.717739000000
H	-13.898221000000	0.611872000000	-0.281129000000
H	-12.713234000000	0.823924000000	1.016217000000
O	6.618194000000	-2.346395000000	-0.810020000000
H	7.564498000000	-2.547196000000	-0.884929000000
O	-6.613284000000	-2.369488000000	-0.811619000000
H	-7.559488000000	-2.568836000000	-0.891657000000

C	-6.318745000000	1.148633000000	-0.527880000000
C	-6.636291000000	0.281060000000	0.524547000000
C	-5.794590000000	0.152281000000	1.627667000000
C	-4.597048000000	0.856152000000	1.667684000000
C	-4.240124000000	1.710735000000	0.604423000000
C	-2.993611000000	2.472982000000	0.576695000000
C	-2.016084000000	2.457089000000	1.510860000000
C	-0.774711000000	3.230025000000	1.420402000000
C	-0.509805000000	4.113285000000	0.195644000000
C	-0.205774000000	3.311655000000	-1.075718000000
C	1.073006000000	2.612067000000	-1.210983000000
C	2.053826000000	2.625518000000	-0.279363000000
C	3.339898000000	1.938464000000	-0.350394000000
C	4.229858000000	2.081329000000	0.728809000000
C	5.470054000000	1.444889000000	0.710260000000
C	5.827918000000	0.665543000000	-0.379902000000
C	4.957860000000	0.509839000000	-1.473018000000
C	3.721003000000	1.145220000000	-1.450587000000
H	-4.869019000000	2.512661000000	-1.309657000000
H	-6.086359000000	-0.500788000000	2.442409000000
H	-3.947217000000	0.742989000000	2.526925000000
H	-2.863121000000	3.093736000000	-0.305217000000
H	-2.090800000000	1.847378000000	2.406432000000
H	-1.380360000000	4.735128000000	-0.020145000000
H	1.175529000000	2.061024000000	-2.141350000000
H	1.896323000000	3.187933000000	0.636657000000
H	3.054194000000	1.020705000000	-2.297573000000
H	3.946247000000	2.688113000000	1.582154000000
H	6.166653000000	1.544058000000	1.535411000000
H	0.330658000000	4.762693000000	0.446596000000
O	6.234405000000	-1.932559000000	0.146317000000
O	-9.082310000000	-2.188638000000	0.169554000000
C	7.191837000000	-1.258852000000	-0.164947000000
C	-7.978575000000	-1.700807000000	0.097272000000
C	8.628156000000	-1.698933000000	-0.257665000000
C	9.408151000000	-1.240715000000	1.022438000000
C	8.808364000000	-3.239242000000	-0.336774000000
H	9.060271000000	-1.207955000000	-1.133708000000
C	10.367768000000	-2.404074000000	1.313925000000
H	8.713009000000	-1.123773000000	1.862236000000
H	9.906988000000	-0.280726000000	0.870847000000
C	9.530970000000	-3.642710000000	0.963995000000
H	9.431334000000	-3.479775000000	-1.203858000000
H	7.852124000000	-3.751387000000	-0.463331000000
H	11.242821000000	-2.343983000000	0.654241000000
H	10.727705000000	-2.398801000000	2.347573000000
H	10.129037000000	-4.551620000000	0.848079000000
H	8.798433000000	-3.826682000000	1.759673000000
C	-6.728328000000	-2.421006000000	-0.343501000000
C	-6.065041000000	-3.155420000000	0.877404000000
C	-7.036526000000	-3.535860000000	-1.385251000000
H	-6.026728000000	-1.694124000000	-0.756247000000
C	-5.720994000000	-4.559790000000	0.351831000000
H	-6.780549000000	-3.236475000000	1.702710000000
H	-5.196560000000	-2.606116000000	1.246163000000
C	-6.856548000000	-4.864815000000	-0.632952000000
H	-6.302504000000	-3.463194000000	-2.194231000000
H	-8.029653000000	-3.424977000000	-1.825284000000
H	-4.762019000000	-4.536031000000	-0.180568000000
H	-5.635237000000	-5.292072000000	1.160407000000
H	-6.634882000000	-5.699917000000	-1.304286000000
H	-7.771615000000	-5.111635000000	-0.081341000000
O	5.391245000000	-0.264224000000	-2.506720000000
H	4.708066000000	-0.324736000000	-3.189412000000
O	-7.146057000000	1.304298000000	-1.597604000000
H	-7.982623000000	0.844095000000	-1.424371000000

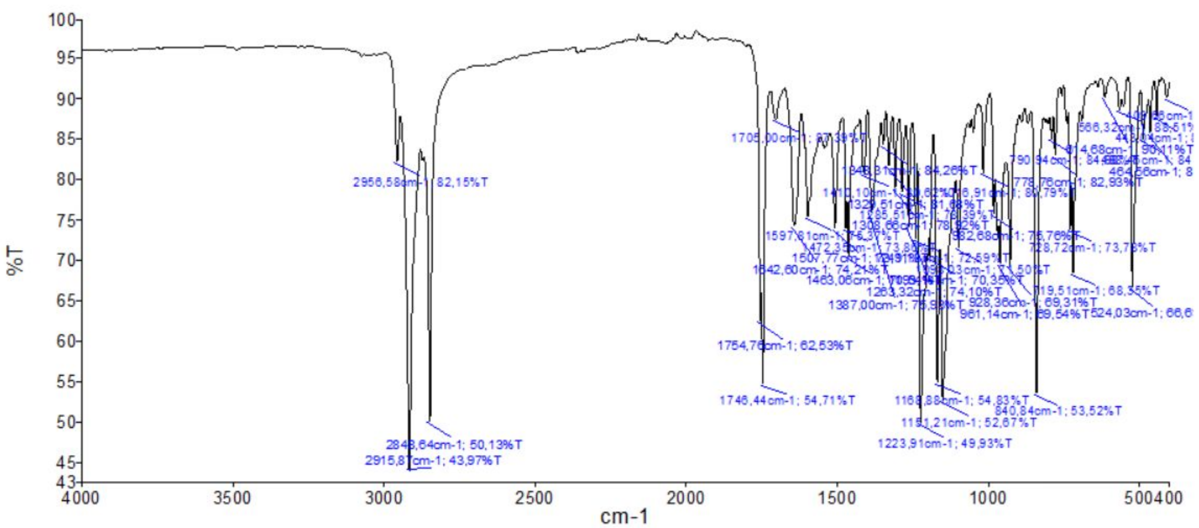

Figure S2: FT- IR Spectrum of HL2b.

Table S2: VEEs (in eV), OSs, NTOs, and natures of transitions of **HL1a** for the first singlet (S1-S3) and triplet (T1-T3) states in DCM (color code: Carbon (yellow) and Oxygen (red)). Hydrogen atoms are removed for the sake of clarity.

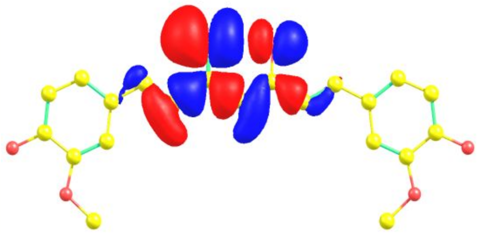

State Information	Virtual	Occupied
**S3**<br>VEE = 3.97<br>OS = 0.00<br>Nature: n → π*<br>Coefficient: ≈ 1		

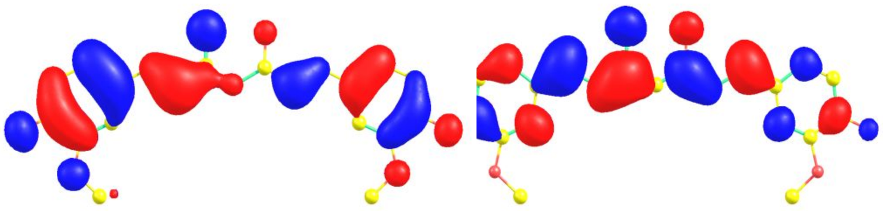

S2
VEE = 3.82
OS = 0.11
Nature: π → π*
Coefficient: 0.88

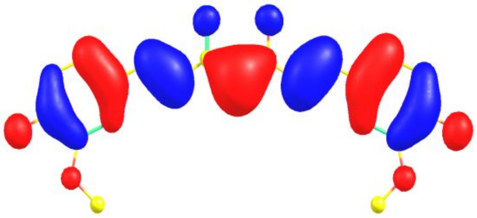

S1
VEE = 3.12
OS = 2.06
Nature: π → π*
Coefficient: 0.93

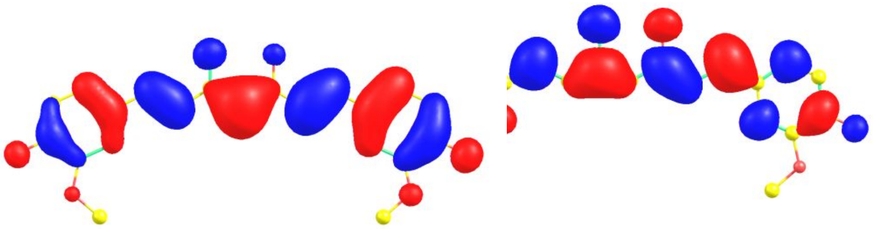

S1 (optimized)

Nature: π → π*
Coefficient: 0.94

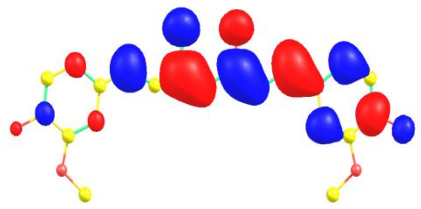

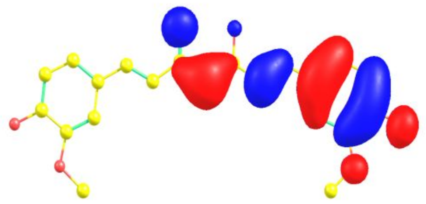

T3
VEE = 2.98
OS = 0.00
Nature: mixed
Coefficient: 0.65

Nature: $\pi \rightarrow \pi^*$
Coefficient: 0.30

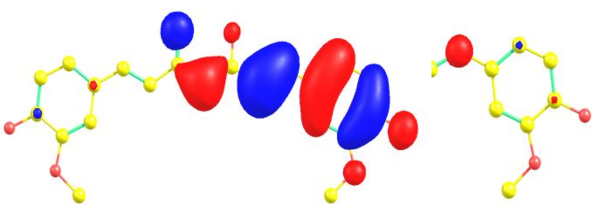

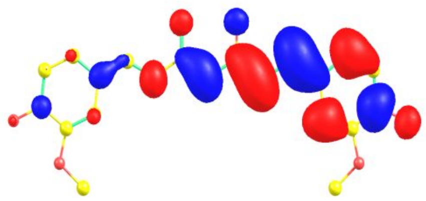

T2
VEE = 2.21
OS = 0.00
Nature: π → π*
Coefficient: 0.83

Nature: π → π*
Coefficient: 0.14

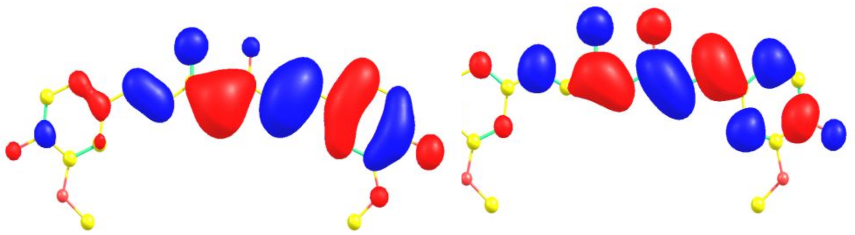

T1
VEE = 1.86
OS = 0.00
Nature: π → π*
Coefficient: 0.90

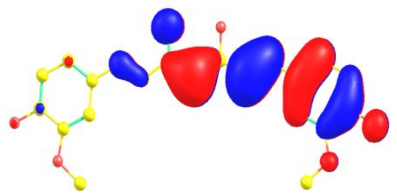

T1 (Optimized)
Nature: $\pi \rightarrow \pi^*$
Coefficient: 0.95

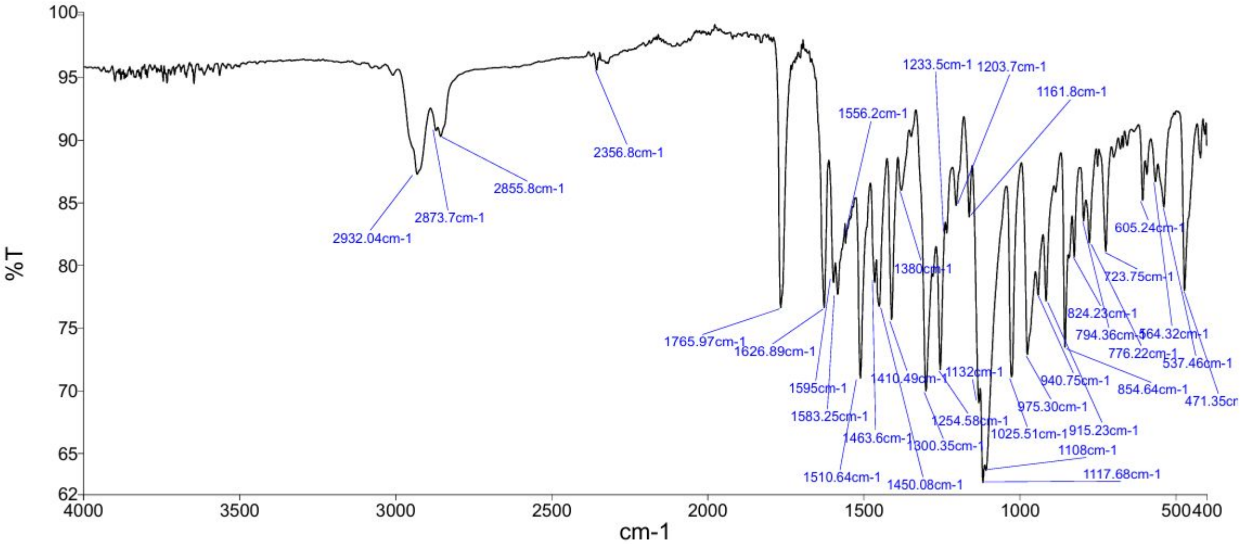

Figure S3: FT- IR Spectrum of HL3a.

Table S3: VEEs (in eV), OSs, NTOs, and natures of transitions of **HL1b** for the first singlet (S1-S3) and triplet (T1-T3) states in DCM (color code: Carbon (yellow) and Oxygen (red) Hydrogen atoms are removed for the sake of clarity.

State Information	Virtual	Occupied
**S3**<br>VEE = 3.97<br>OS = 0.00<br>Nature: n → π*<br>Coefficient: ≈ 1	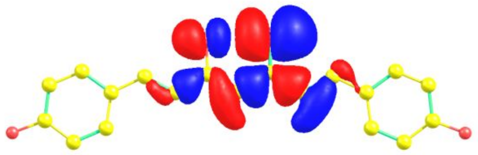	
**S2**<br>VEE =3.94<br>OS = 0.13<br>Nature: π → π*<br>Coefficient: 0.89	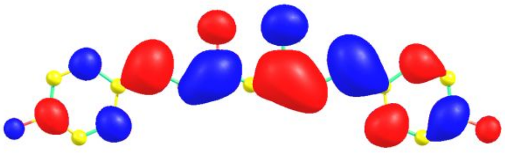	
**S1**<br>VEE = 3.18<br>OS = 2.04<br>Nature: π → π*<br>Coefficient: 0.93	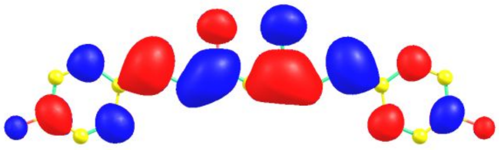	
**S1 (Optimized)**<br>Nature: π → π*<br>Coefficient: 0.95	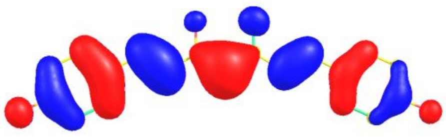	

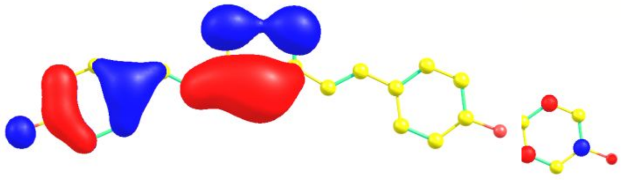

T3
VEE = 3.04
OS = 0.00
Nature: mixed
Coefficient: 0.63

Coefficient: 0.30

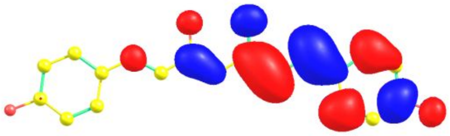

T2
VEE = 2.27
OS = 0.00
Nature: π → π*
Coefficient: 0.83

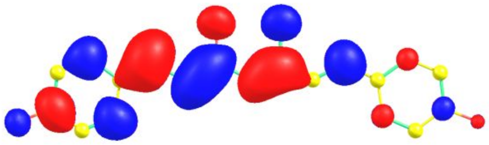

T1
VEE = 1.90
OS = 0.00
Nature: π → π*
Coefficient: 0.91

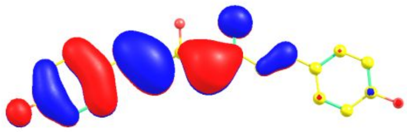

T1 (Optimized)

Nature: $\pi \rightarrow \pi^*$
Coefficient: 0.95

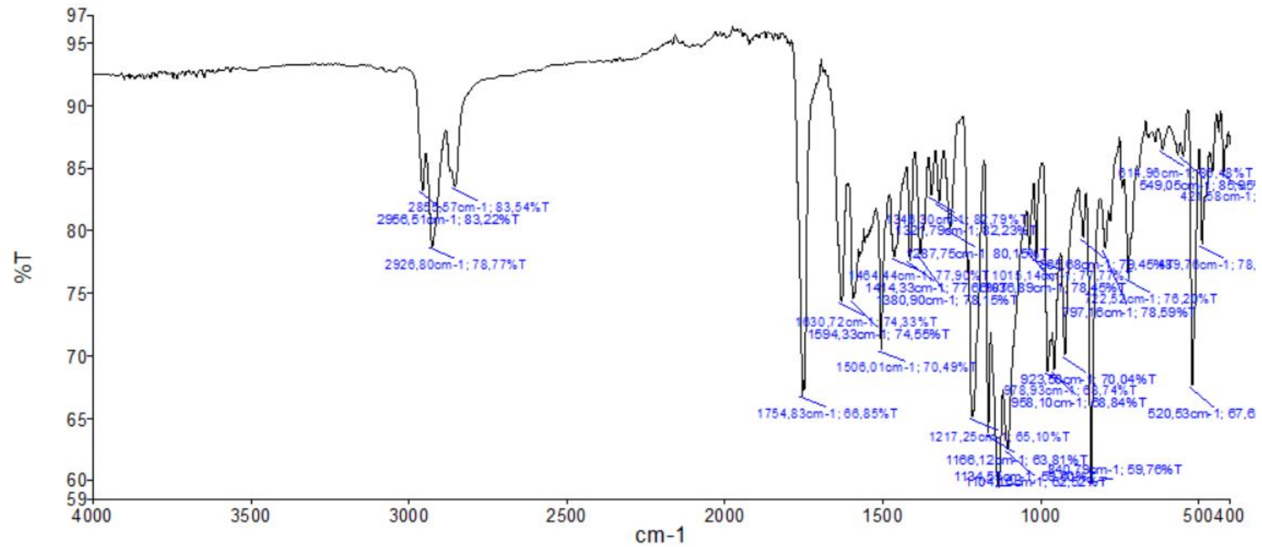

Figure S4: FT- IR Spectrum of HL3b.

Table S4: VEEs (in eV), OSs, NTOs, and natures of transitions of **HL3a** for the first singlet (S1-S3) and triplet (T1-T3) states in DCM (color code: Carbon (yellow) and Oxygen (red)). Hydrogen atoms are removed for the sake of clarity.

State Information	Virtual	Occupied

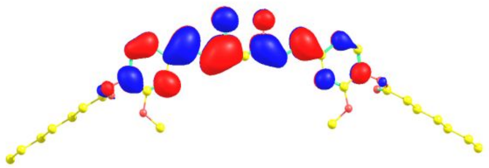

S3
VEE = 4.01
OS = 0.09
Nature: π → π*
Coefficient: 0.86

Coefficient: 0.12

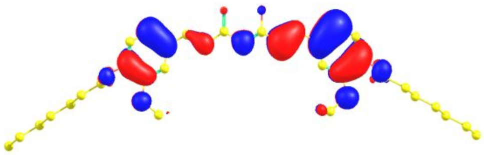

S2
VEE = 3.91
OS = 0.0
Nature: n → π*
Coefficient: ≈ 1

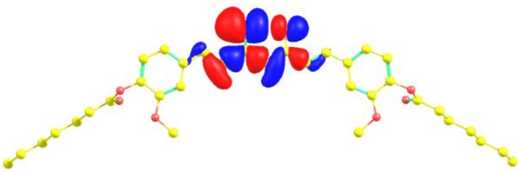

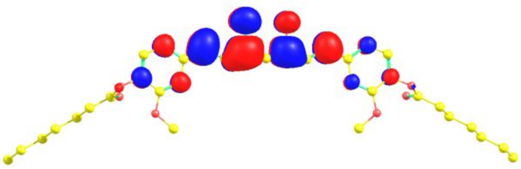

S1
VEE = 3.27
OS = 2.1
Nature: π → π*
Coefficient: 0.94

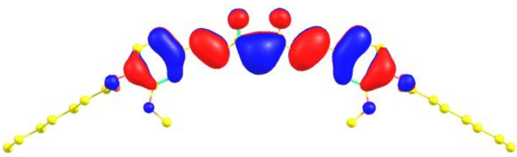

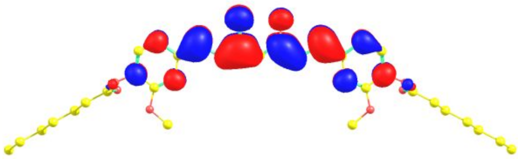

T3
VEE = 2.99
OS = 0.0
Nature: mixed
Coefficient: 0.63

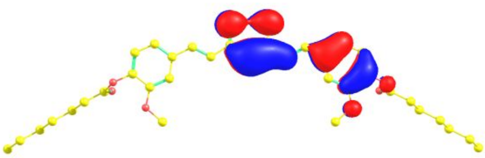

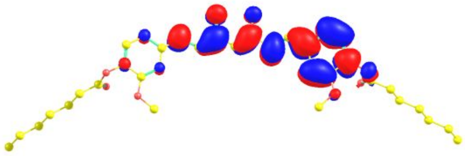

Coefficient: 0.30

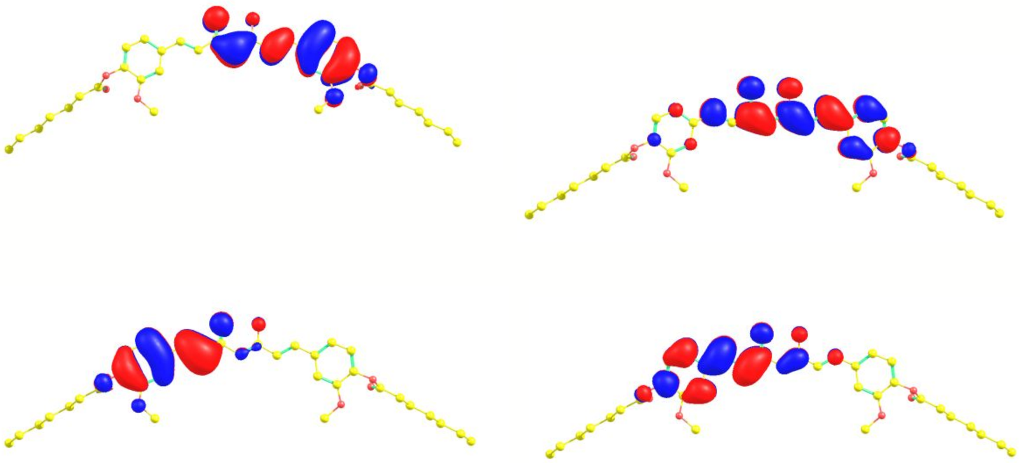

T2
VEE = 2.28
OS = 0.0
Nature: π → π*
Coefficient: 0.85

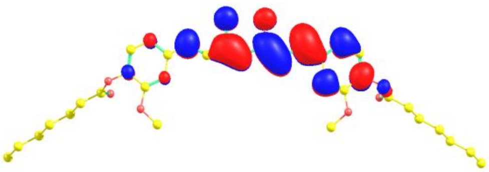

T1
VEE = 1.91
OS = 0.0
Nature: π → π*
Coefficient: 0.91

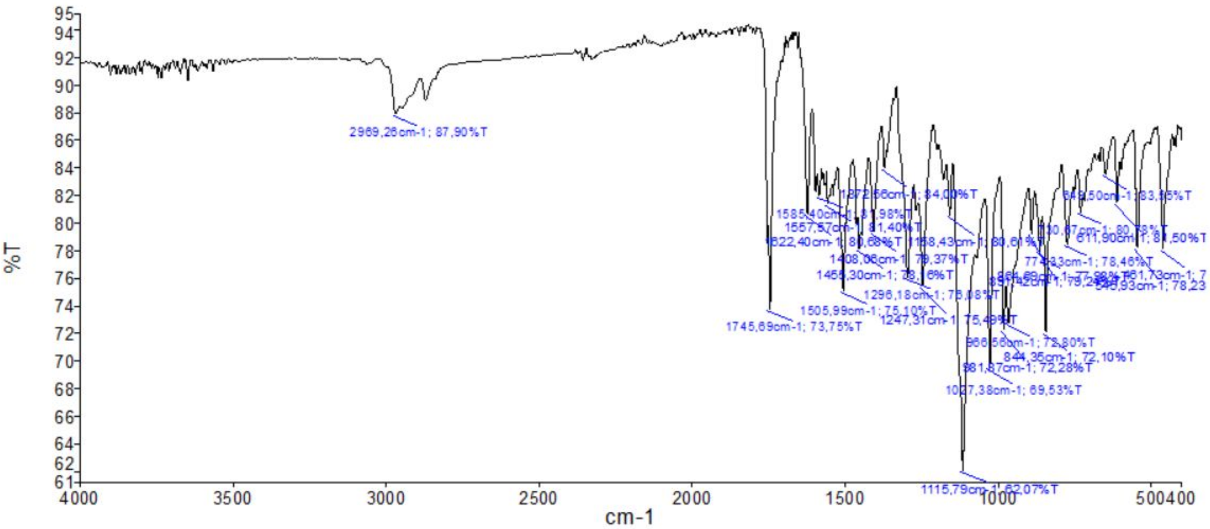

Figure S5: FT- IR Spectrum of HL4a.

Table S5: VEEs (in eV), OSs, NTOs, and natures of transitions of **HL3b** for the first singlet (S1-S3) and triplet (T1-T3) states in DCM (color code: Carbon (yellow) and Oxygen (red) Hydrogen atoms are removed for the sake of clarity.

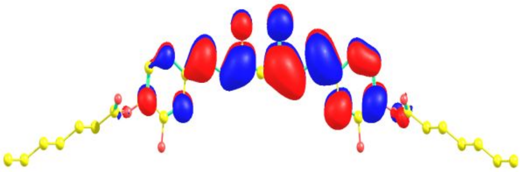

**State Information**	**Virtual**	**Occupied**
**S3**<br>VEE = 4.04<br>OS = 0.09<br>Nature: $\pi \rightarrow \pi^*$<br>Coefficient: 0.86		

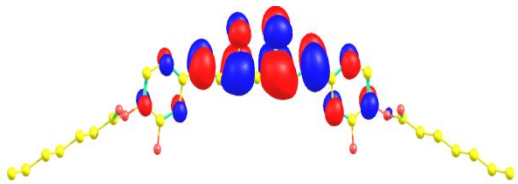

S2
VEE = 3.90
OS = 0.0
Nature: n → π*
Coefficient: ≈ 1

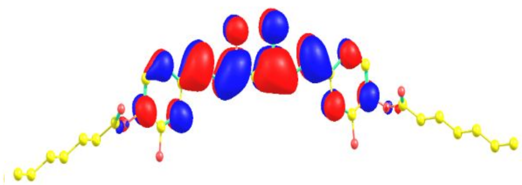

S1
VEE = 3.28
OS = 2.13
Nature: π → π*
Coefficient: 0.94

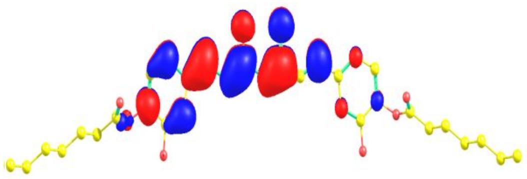

T3
VEE =3.01
OS = 0.0
Nature: mixed
Coefficient: 0.63

Coefficient: 0.30

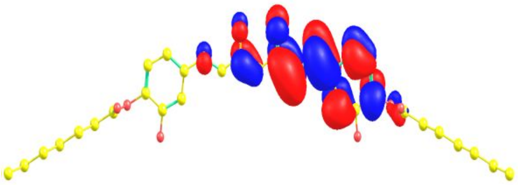

T2
VEE = 2.30
OS = 0.0
Nature: π → π*
Coefficient: 0.85

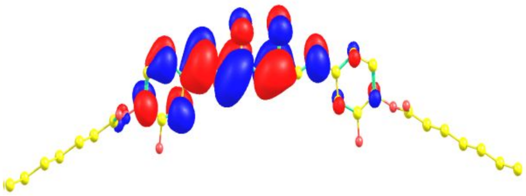

T1
VEE = 1.91
OS = 0.0
Nature: π → π*
Coefficient: 0.92

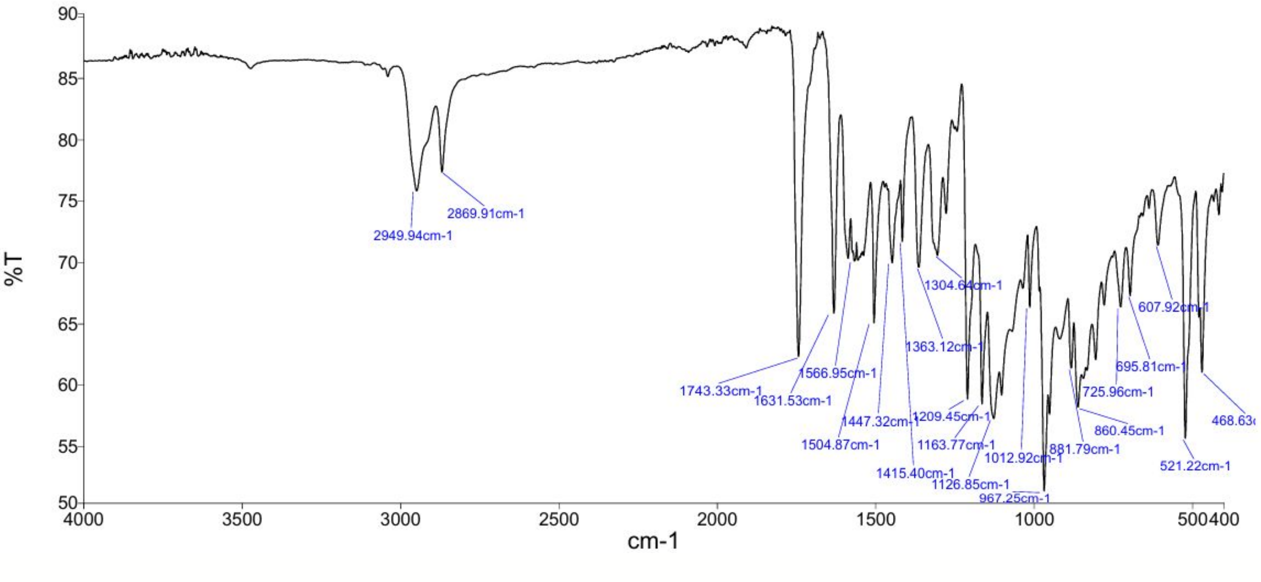

Figure S6: FT- IR Spectrum of HL4b.

Table S6: VEEs (in eV), OSs, NTOs, and natures of transitions of **HL4b** for the first singlet (S1-S3) and triplet (T1-T3) states in DCM (color code: Carbon (yellow) and Oxygen (red)). Hydrogen atoms are removed for the sake of clarity.

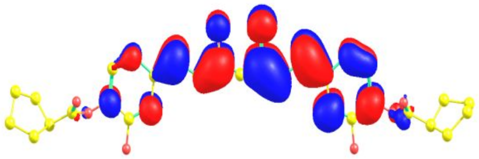

State Information	Virtual	Occupied

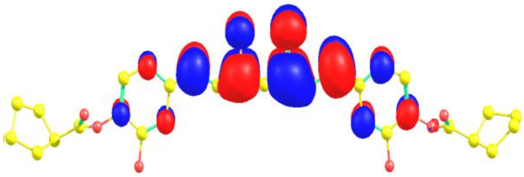

S3
VEE = 4.32
OS = 0.03
Nature: π → π*
Coefficient: 0.86

S2
VEE = 3.90
OS = 0.0
Nature: n → π*
Coefficient: ≈ 1

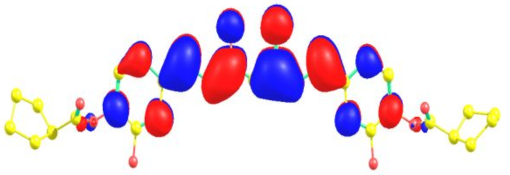

S1
VEE = 3.28
OS = 2.16
Nature: π → π*
Coefficient: 0.93

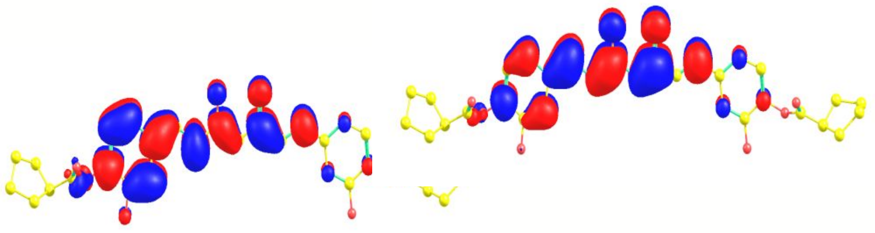

T3
VEE = 3.05
OS = 0.0
Nature:
Coefficient: 0.62

Coefficient: 0.30

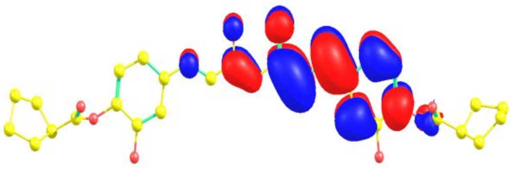

T2
VEE = 2.30
OS = 0.0
Nature:
Coefficient: 0.85

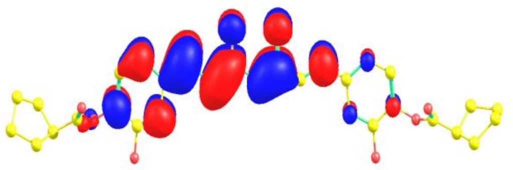

T1
VEE = 1.91
OS = 0.0
Nature:
Coefficient: 0.92

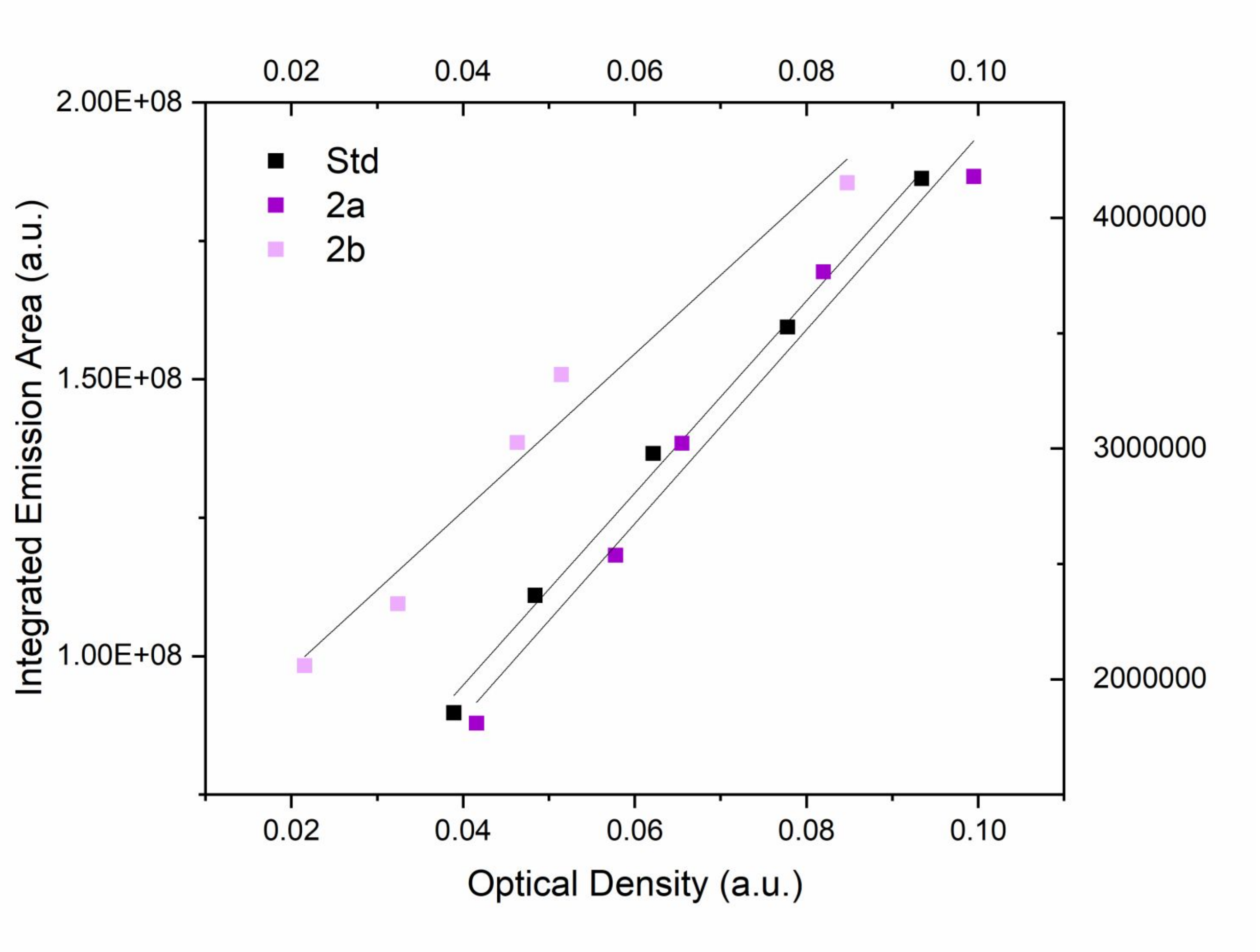

Figure S7: Linear relationship between the integrated emission area and the optical density of curcumins **HL2a** and **HL2b** in DCM. The same relationship is reported for the reference quinine sulfate (Std; 1N aqueous solution of sulfuric acid). λ_{exc} = 345 nm.

Table S7*:* Computed excitation energies (in eV) and wavelengths (in nm) of curcumin for the lowest singlet and triplet states in DCM using B3LYP/6-31++G(d,p)//TD-DFT(CAM-B3LYP/6-31++G(d,p)) (Approach I) and CAM-B3LYP/6-31++G(d,p)//TD-DFT(CAM-B3LYP/6-31++G(d,p)) (Approach II), together with the corresponding energy differences.

Quantities (Units)	State	Approach I	Approach II	Difference
VEE(Sn) (eV) [λ (nm)]	S1	3.12 [397]	3.23 [384]	0.11 [13]
	S2	3.82 [324]	3.90 [318]	0.08 [6]
	S3	3.97 [312]	4.01 [309]	0.04 [3]
VEE(Tn) (eV) [λ (nm)]	T1	1.86 [666]	1.98 [625]	0.12 [41]
	T2	2.21 [562]	2.31 [537]	0.10 [25]
	T3	2.98 [416]	3.05 [406]	0.07 [10]

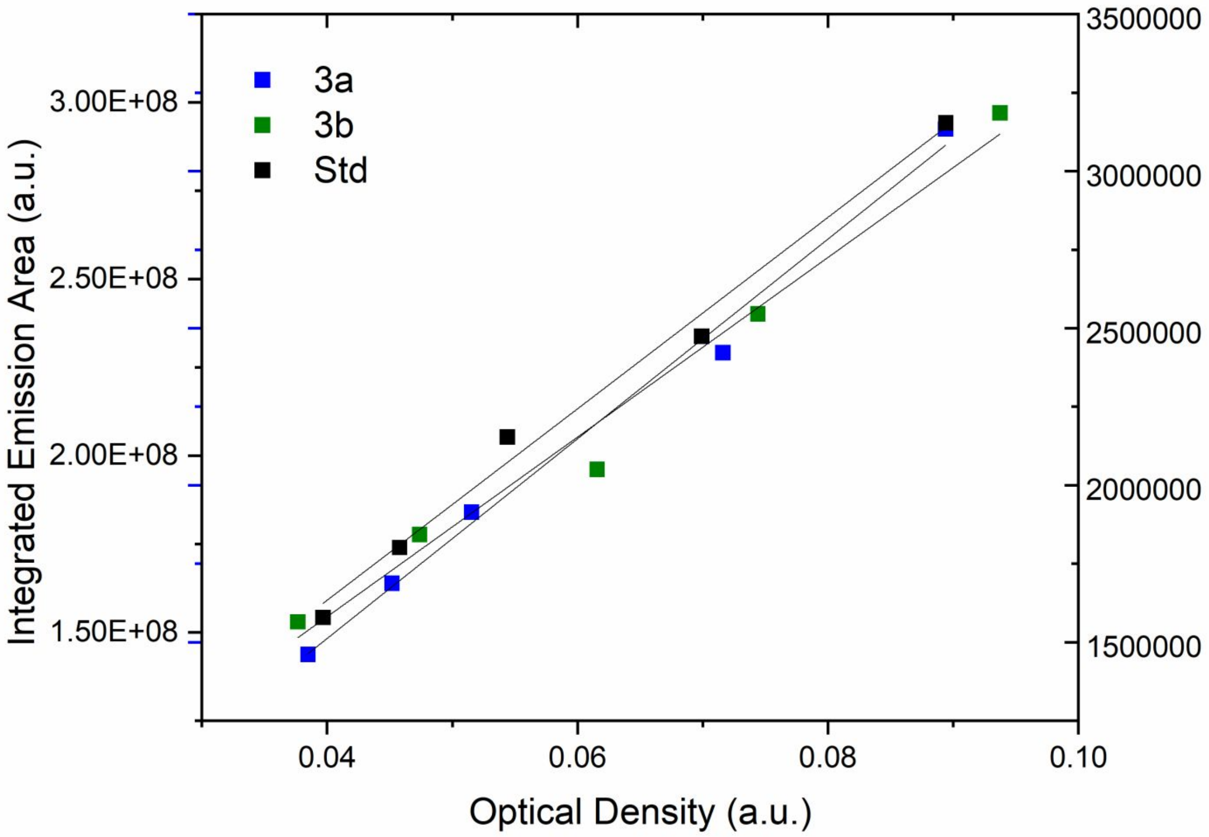

Figure S8: Linear relationship between the integrated emission area and the optical density of curcumins **HL3a** and **HL3b** in DCM. The same relationship is reported for the reference quinine sulfate (Std; 1N aqueous solution of sulfuric acid). λ_{exc} = 345 nm.

Table S8: The computed triplet excitation energies of curcumin in DCM using CAM-B3LYP and ωB97XD density functionals.

**Quantities (Units)**	**State**	**CAM-B3LYP + GD3BJ**	**ωB97XD**	**Difference**
VEE(Sn) (eV) [λ (nm)]	S1	3.12 [397][a]	3.19 [389][a]	0.07 [8]
	S2	3.82 [324][a]	3.92 [317][a]	0.10 [7]
	S3	3.97 [312][a]	3.93 [315][a]	0.04 [3]
VEE(Tn) (eV) [λ (nm)]	T1	1.86 [666][a]	1.96 [633][a]	0.10 [33]
	T2	2.21 [562][a]	2.32 [535][a]	0.11 [27]
	T3	2.98 [416][a]	3.10 [406][a]	0.12 [10]

VPE(T1) (eV) [λ (nm)]	T1→ S0	1.21 [1026][b]	1.28 [971][b]	0.07 [55]
			1.30 [952][c]	0.11 [74]

a. Calculations were performed on the ground-state (S0) geometry optimized at the B3LYP/6-31++G(d,p) level of theory.

b. Calculations were performed on the first triplet (T_1) geometry optimized at the CAM-B3LYP/6-31++G(d,p) level of theory.

c. Calculations were performed on the first triplet (T_1) geometry optimized at the ωB97XD/6-31++G(d,p) level of theory.

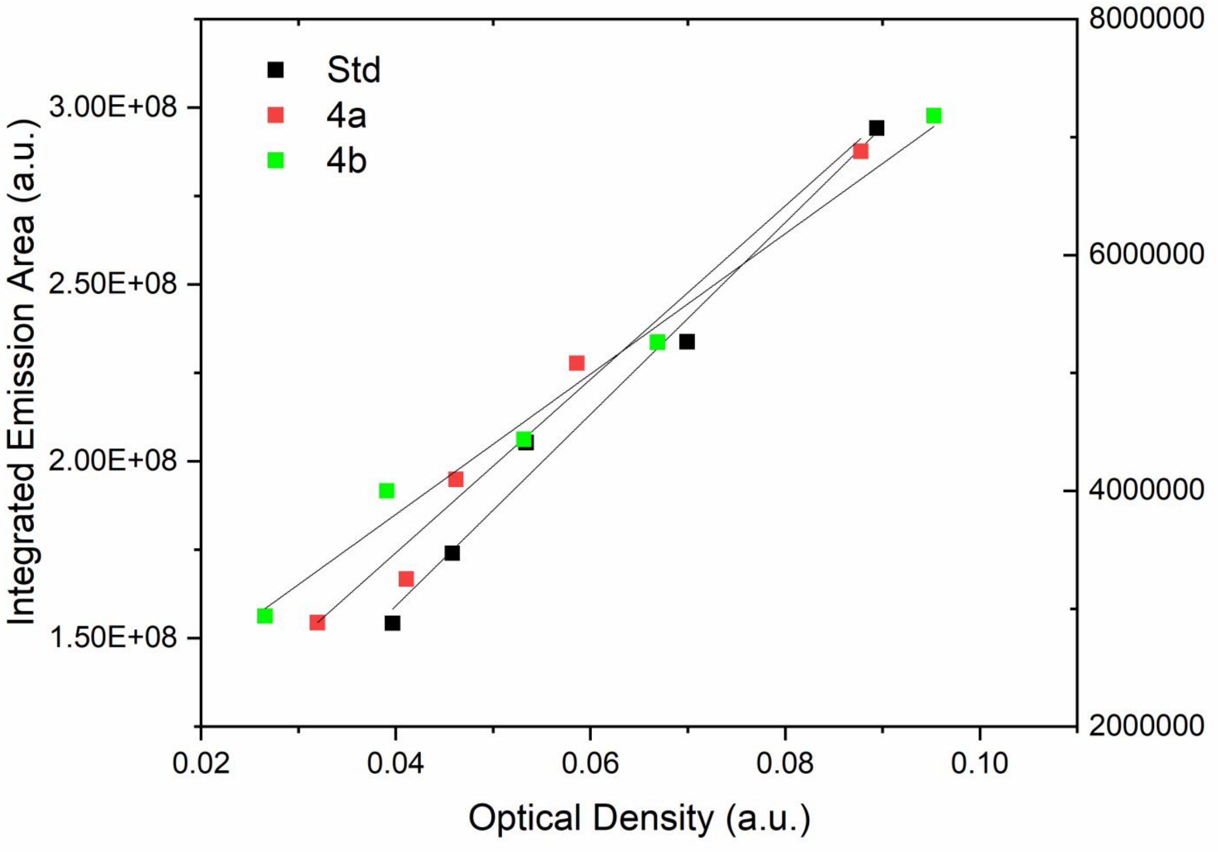

Figure S9: Linear relationship between the integrated emission area and the optical density of curcumins **HL4a** and **HL4b** in DCM. The same relationship is reported for the reference quinine sulfate (Std; 1N aqueous solution of sulfuric acid). λ_{exc} = 345 nm.

Table S9: Comparison of linear-response (LR) and corrected linear-response (Corrected LR) effects on the triplet energy calculations of curcumin in dichloromethane.

Quantities (Units)	State	LR (Default)	Corrected LR	Difference
VEE(Sn) (eV) [λ (nm)]	S1	3.12 [397][a]	3.12 [396][a]	0 [0]
	S2	3.82 [324][a]	3.82 [324][a]	0 [0]
	S3	3.97 [312][a]	3.97 [321][a]	0 [0]
VEE(T1) (eV) [λ (nm)]	T1	1.86 [666]	1.86 [666]	0 [0]
	T2	2.21 [562]	2.21 [562]	0 [0]
	T3	2.98 [416]	2.98 [416]	0 [0]
VPE(T1) (eV) [λ (nm)]	T1→ S0	1.21 [1026]	1.21 [1026]	0 [0]

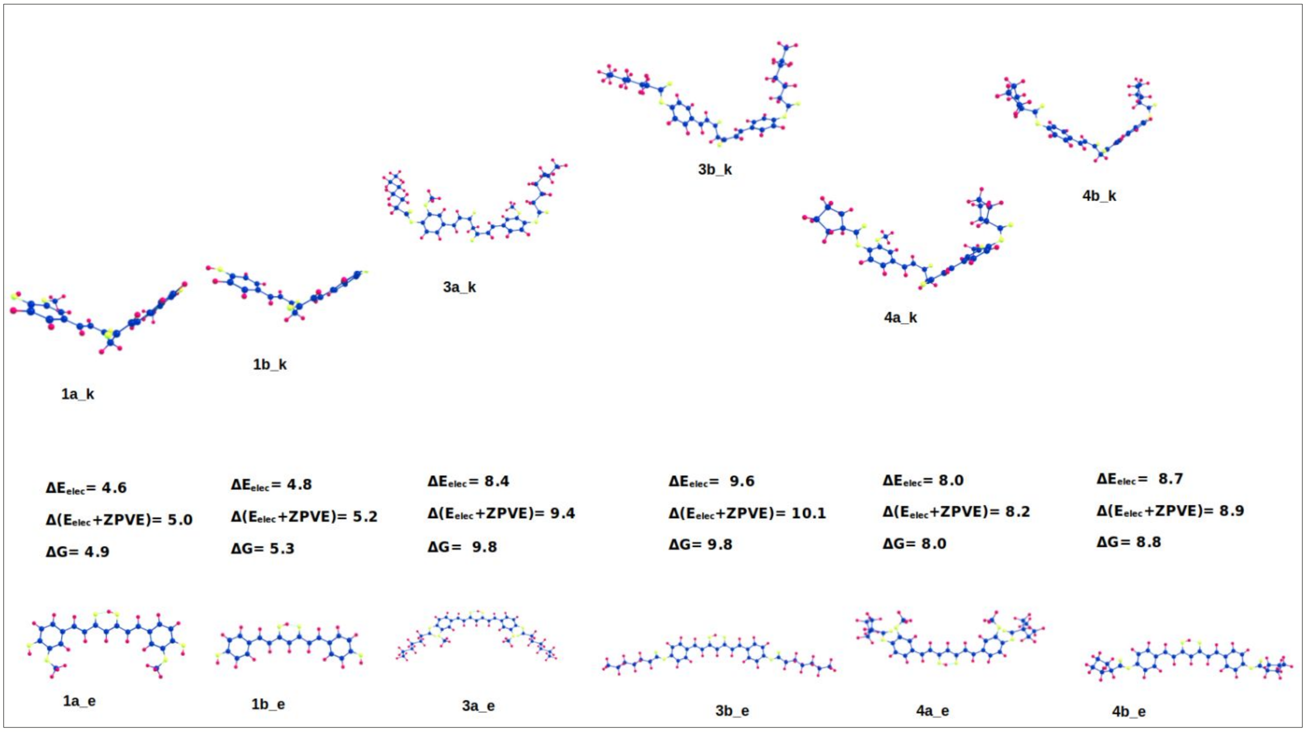

Figure S10: Relative electronic energies, zero point vibrational energy corrected electronic energies, and Gibbs free energies (all in kcal/mol) of the tautomers (k: keto and e: enol) of the studied curcumins computed at B3LYP/6-31++G(d,p) level of theory in the gas phase.

Table S10: First three computed singlet and triplet excitation energies (in eV.) of curcumin in DCM at the B3LYP/6-31++G(d,p)//TD-DFT CAM-B3LYP/6-31++G(d,p) with Gaussian (Full DFT and TDA) and ORCA (TDA), together with the corresponding energy differences.

**Quantities (Unit)**	**State**	**Gaussian (Full DFT)**	**Gaussian (TDA)**	**ORCA 6 (TDA)**	**Difference**
	S1	3.12	3.23	3.20	0.03
VEE(Sn) (eV)	S2	3.82	3.99	3.95	0.04
	S3	3.97	3.99	4.00	0.01
	T1	1.86	2.20	2.20	0.00
VEE(Tn) (eV)	T2	2.21	2.55	2.56	0.01
	T3	2.98	3.18	3.19	0.01

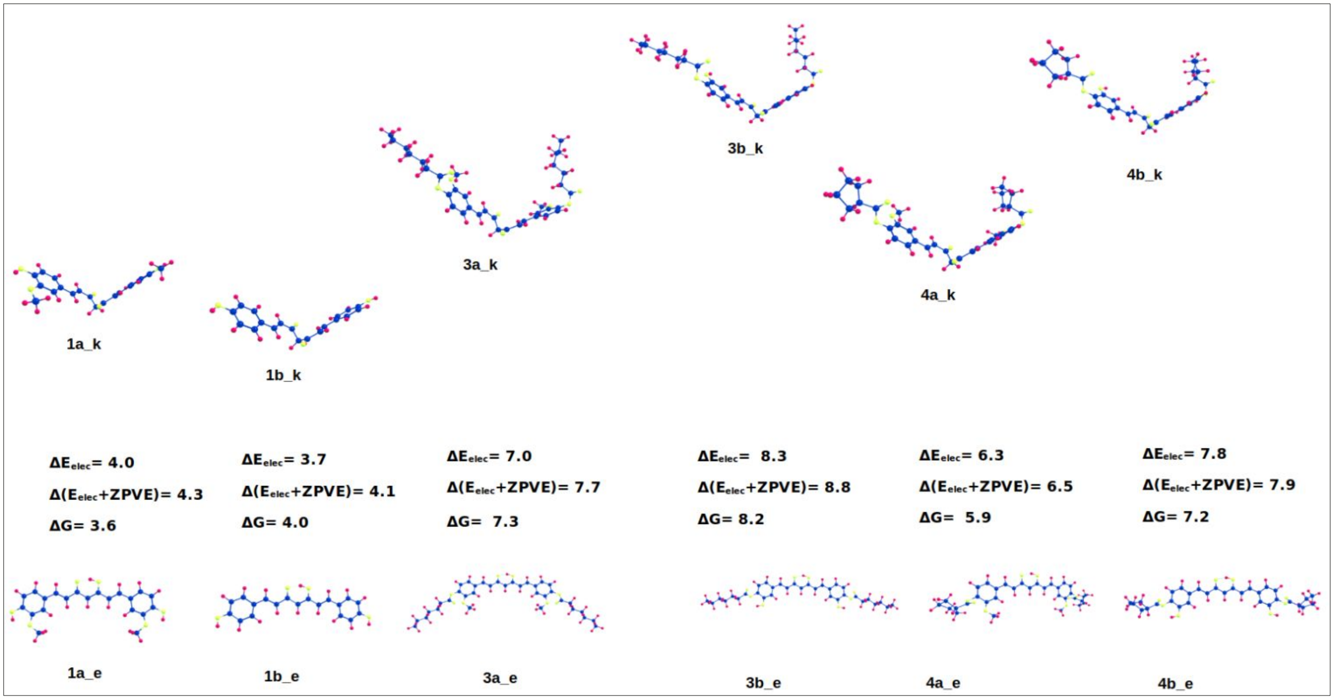

Figure S11: Relative electronic energies, zero point vibrational energy corrected electronic energies, and Gibbs free energies (all in kcal/mol) of the tautomers (k: keto and e: enol) of the studied curcumins computed at B3LYP/6-31++G(d,p) level of theory in DCM

Table S11: Computed SOC matrix elements of curcumin (HX, HY, and HZ) and H (SOC) in cm^{-1}.

States	H_X (cm^{-1})	H_Y (cm^{-1})	H_Z (cm^{-1})	H (SOC) (cm^{-1})
S0 , T1	0.06	0.47	0.2	0.51
S0 , T2	0.02	0.18	0.15	0.24
S0 , T3	0.04	0.2	0.38	0.43
S1 , T1	0.01	0.01	0.01	0.02
S1 , T2	0.16	0.18	0.01	0.24
S1 , T3	0.14	0.28	0.01	0.31
S2 , T1	0.17	0.12	0.03	0.21
S2, T2	0.04	0.03	0.02	0.05

S2, T3	0.08	0.07	0.02	0.11
S3 , T1	0.50	10.83	0.12	10.84
S3 , T2	1.12	11.57	0.10	11.62
S3 , T3	0.38	23.34	0.07	23.34

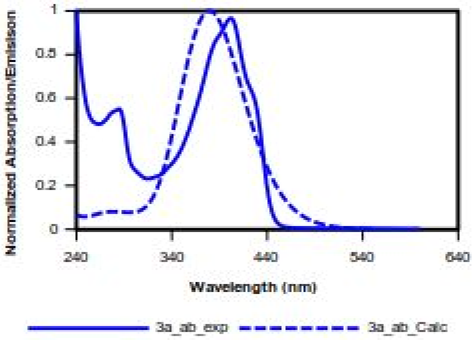
Normalized Absorption/Emisison
Wavelength (nm)
3a_ab_exp
3a_ab_Calc

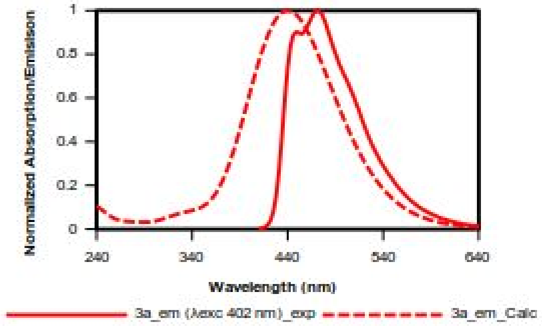
Normalized Absorption/Emisison
Wavelength (nm)
3a_em (λexc 402 nm)_exp
3a_em_Calc

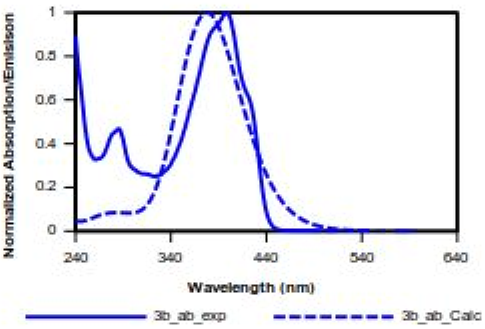
Normalized Absorption/Emisison
Wavelength (nm)
3b_ab_exp
3b_ab_Calc

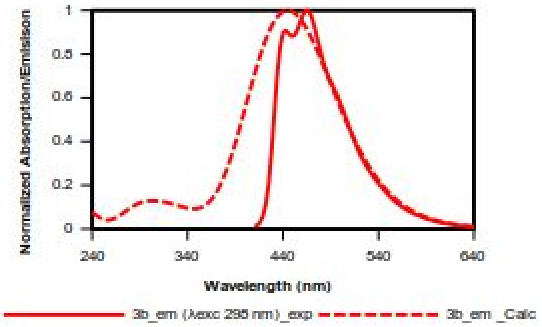
Normalized Absorption/Emisison
Wavelength (nm)
3b_em _Calc

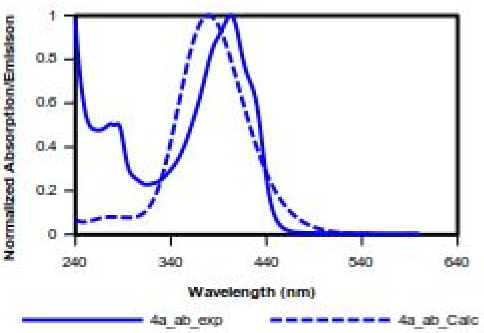
Normalized Absorption/Emisison
Wavelength (nm)
4a_ab_exp
4a_ab_Calc

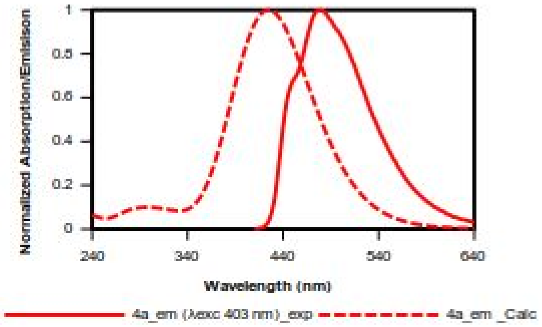
Normalized Absorption/Emisison
Wavelength (nm)
4a_em (λexc 403 nm)_exp
4a_em _Calc

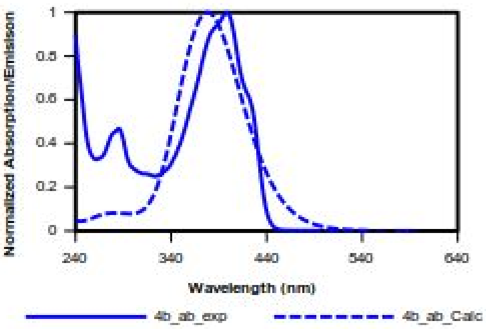
Normalized Absorption/Emisison
Wavelength (nm)
4b_ab_exp
4b_ab_Calc

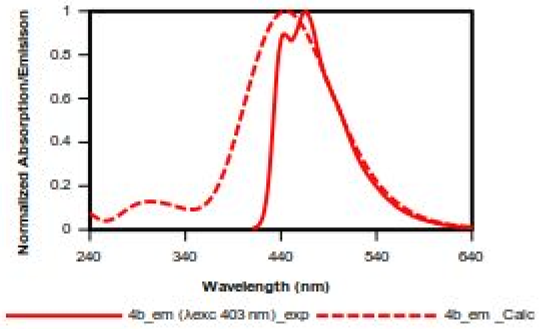
Normalized Absorption/Emisison
Wavelength (nm)
4b_em (λexc 403 nm)_exp
4b_em _Calc

Figure S12: Overlay of measured and computed UV-Vis absorption and emission spectra of the curcumins (**HL3a** and **HL3b**, **HL4a** and **HL4b**) in DCM.

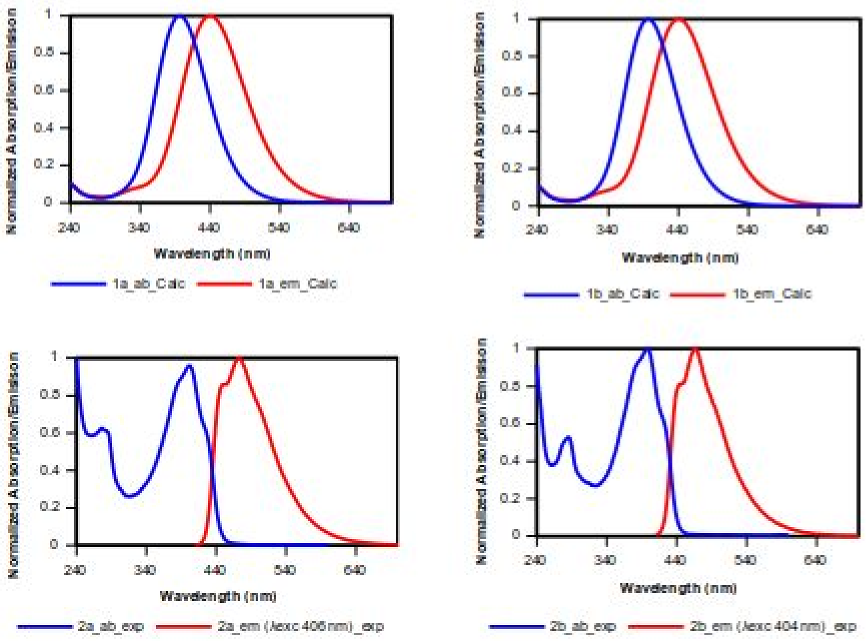

Figure S13: Computed absorption and emission spectra for **HL1a** and **HL1b** together with measured spectra **HL2a** and **HL2b** in DCM.

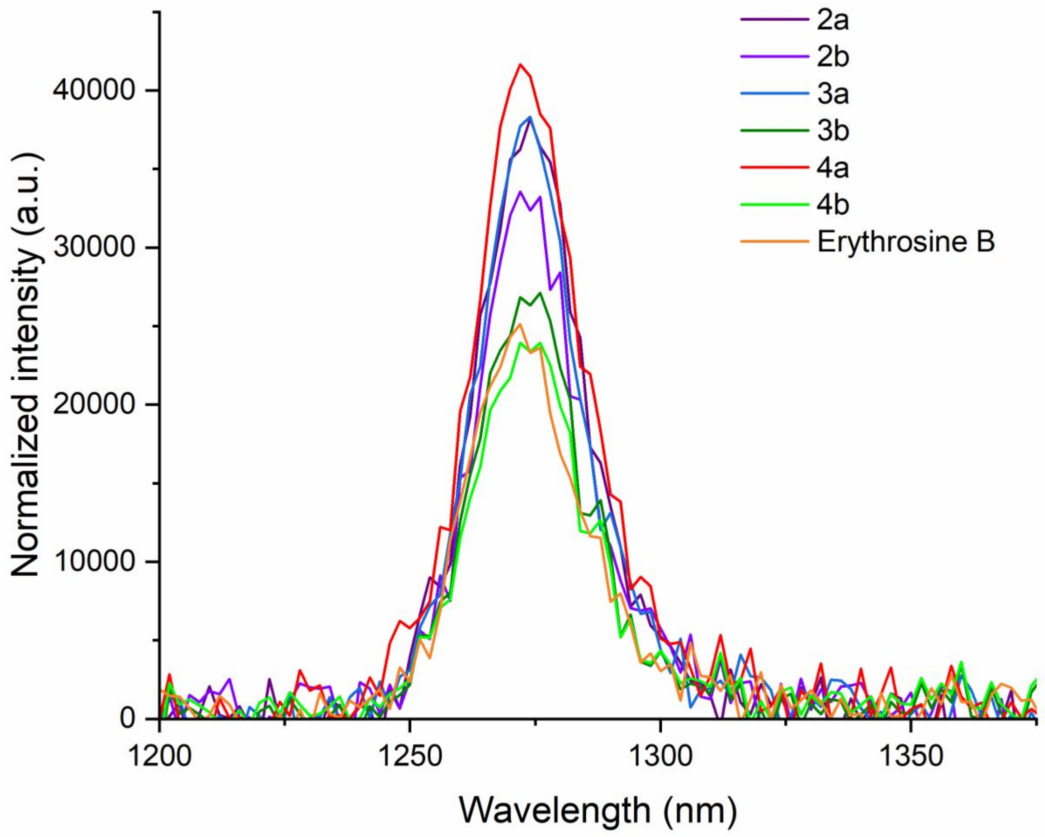

Figure S14: $^{1}O_2$ emission spectra obtained upon excitation of the different curcumins in the Vis (λ_{exc} = 402 nm) in DCM and of erythrosin B (λ_{exc} = 535nm) in ethanol, collected under the same experimental conditions (Abs ~ 0.3-0.5) and normalized for the corresponding absorbance.

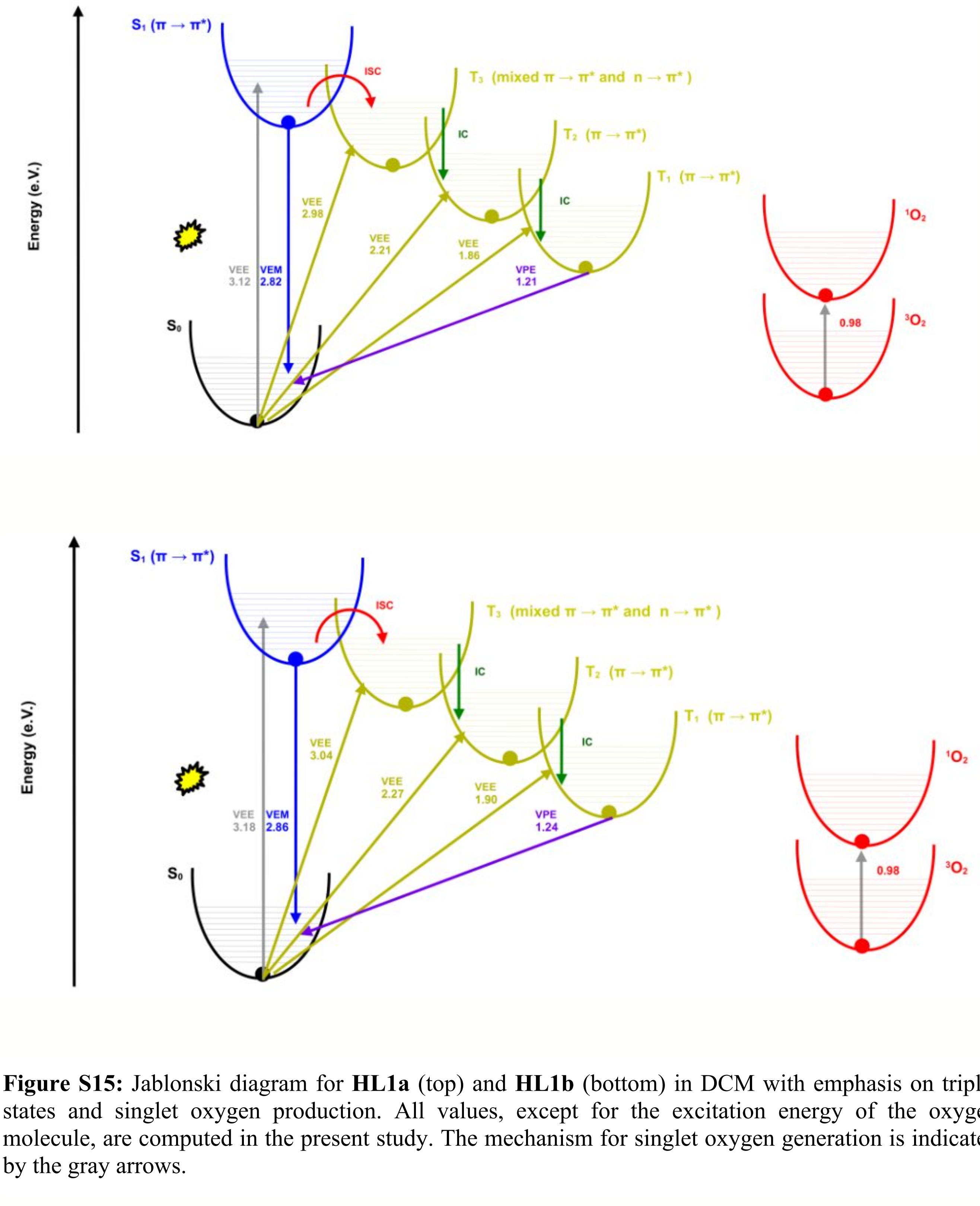

Figure S15: Jablonski diagram for **HL1a** (top) and **HL1b** (bottom) in DCM with emphasis on triplet states and singlet oxygen production. All values, except for the excitation energy of the oxygen molecule, are computed in the present study. The mechanism for singlet oxygen generation is indicated by the gray arrows.

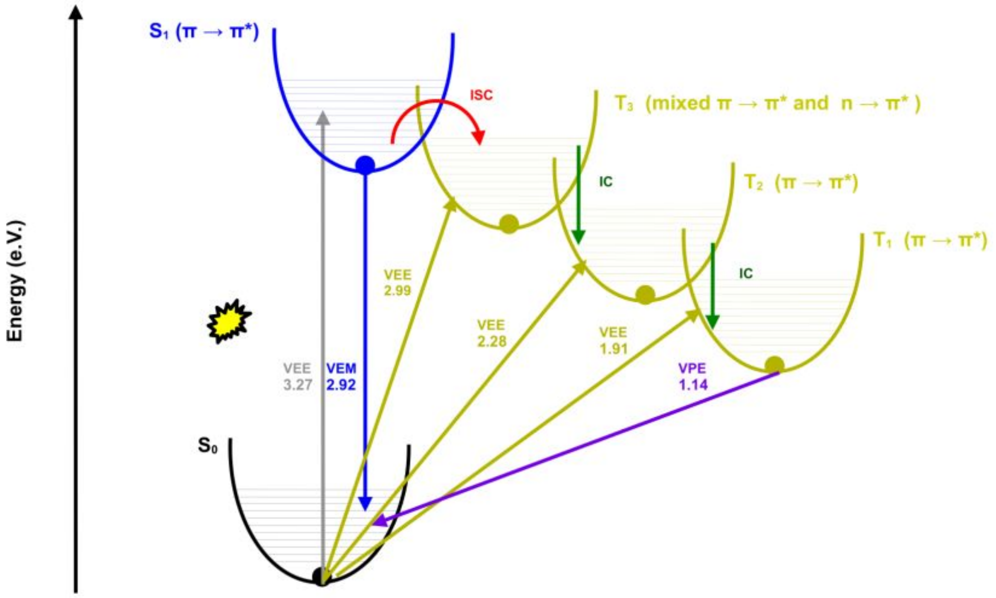
S₁ (π → π*)
ISC
T₃ (mixed π → π* and n → π*)
IC
T₂ (π → π*)
T₁ (π → π*)
IC
Energy (e.V.)
VEE 2.99
VEE 2.28
VEE 1.91
VEE 3.27
VEM 2.92
VPE 1.14
S₀
¹O₂
³O₂
0.98

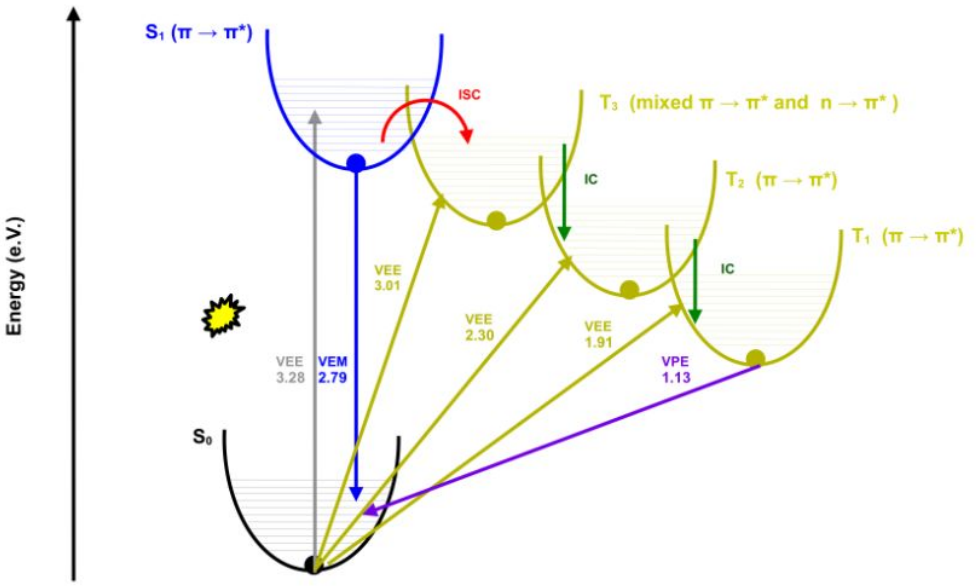
S₁ (π → π*)
ISC
T₃ (mixed π → π* and n → π*)
IC
T₂ (π → π*)
T₁ (π → π*)
IC
Energy (e.V.)
VEE 3.01
VEE 2.30
VEE 1.91
VEE 3.28
VEM 2.79
VPE 1.13
S₀
¹O₂
³O₂
0.98

Figure S16: Jablonski diagram for HL3a (up) and HL3b (down) in DCM with emphasis on triplet states and singlet oxygen production. All values, except for the excitation energy of the oxygen molecule, are computed in the present study. The mechanism for singlet oxygen generation is indicated by the gray arrows.

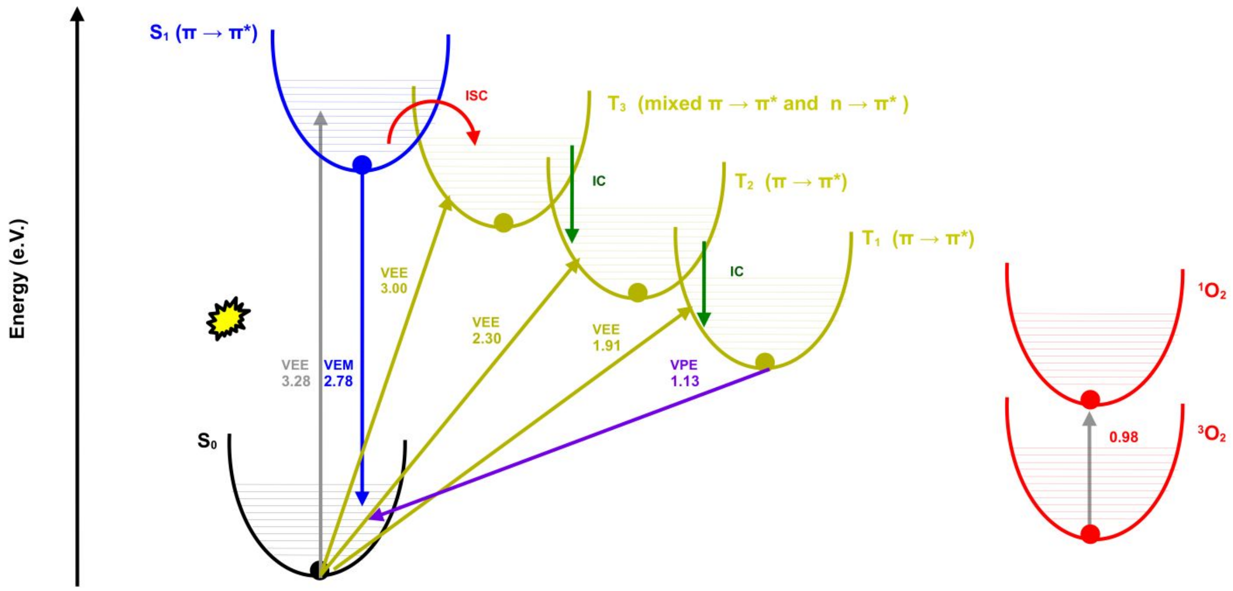

Figure S17: Jablonski diagram for **HL4b** in DCM with emphasis on triplet states and singlet oxygen production. All values, except for the excitation energy of the oxygen molecule, are computed in the present study. The mechanism for singlet oxygen generation is indicated by the gray arrows.

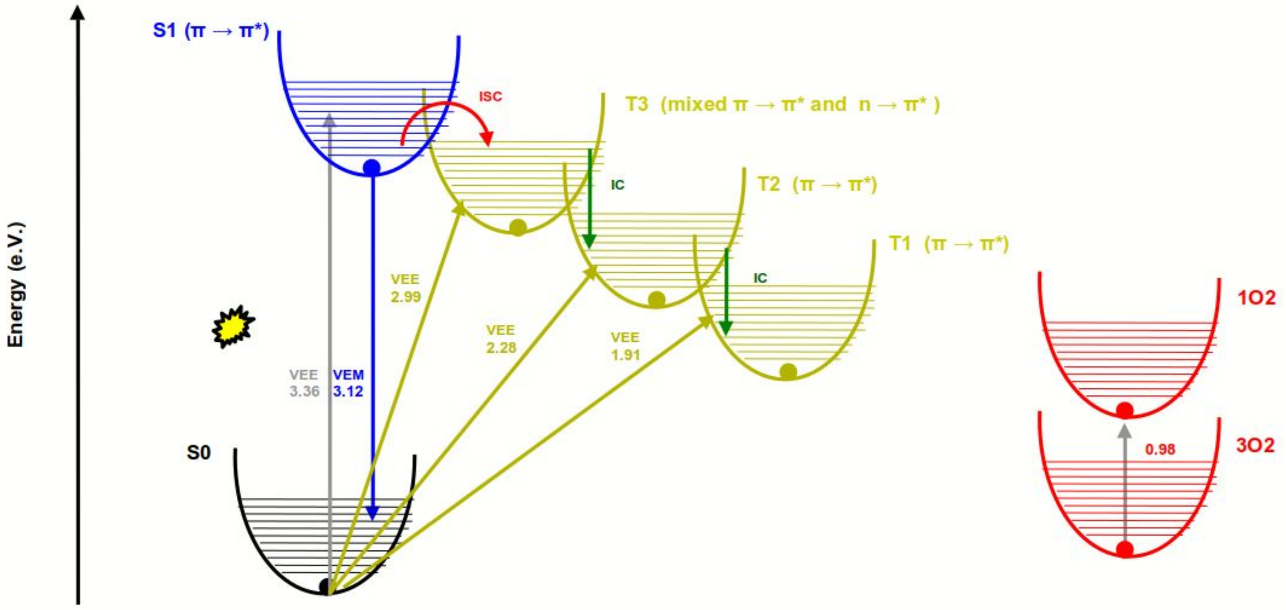

Energy (e.V.)
S1 (π → π*)
ISC
T3 (mixed π → π* and n → π*)
IC
T2 (π → π*)
T1 (π → π*)
IC
1O2
VEE
2.99
VEE
2.28
VEE
1.91
VEE
3.36
VEM
3.12
S0
0.98
3O2

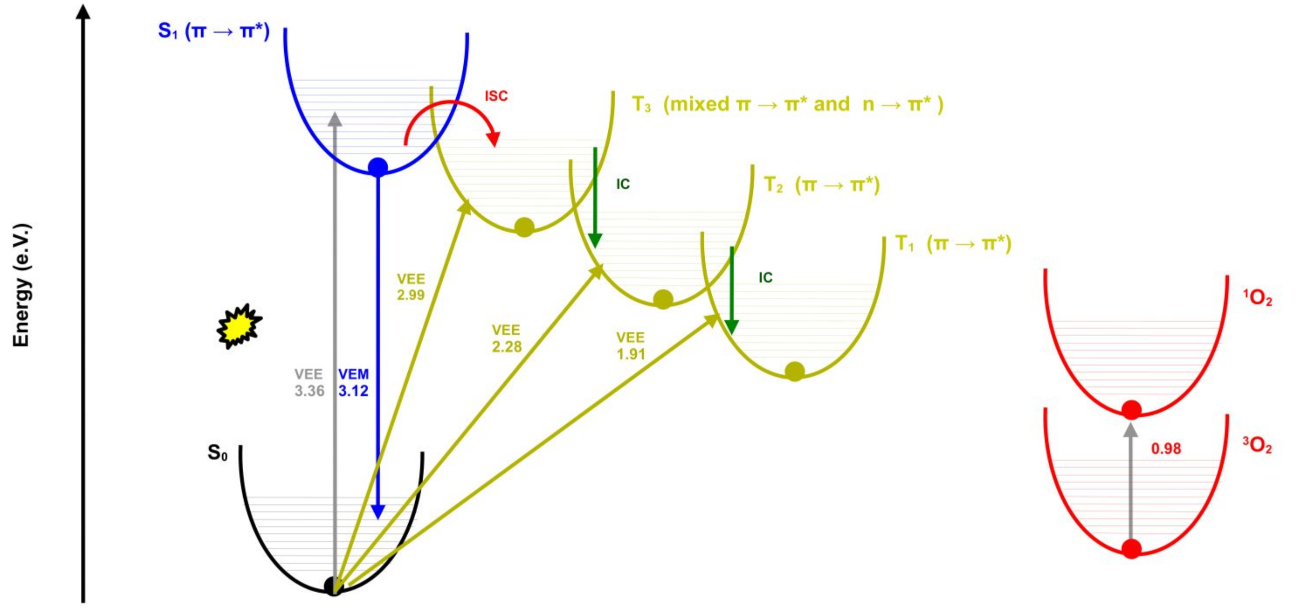

Figure S18: Jablonski diagram for **HL3a** in MeCY (up) and MeOH (down) with emphasis on triplet states and singlet oxygen production. The corresponding diagrams for the other curcumin derivatives are included in the SI. VEE, VEM, ISC, IC, and VPE stand for Vertical Excitation Energy, Vertical Emission Energy, Inter-System Crossing, Internal Conversion, and Vertical Phosphorescence Energy, respectively.
